# Protonation of Dppbz- and Binap-Ligated Rhodathiaboranes Yielding Hydron, Hydride, and Hydrogen Exchange

**DOI:** 10.1021/acs.inorgchem.4c02652

**Published:** 2024-10-08

**Authors:** Javier Vidondo, Laura Urdániz, Pablo J. Sanz Miguel, Ricardo Rodríguez, Ramón Macías

**Affiliations:** Departamento de Química Inorgánica, Instituto de Síntesis Química y Catálisis Homogénea (ISQCH), 16765 Universidad de Zaragoza-CSIC, Zaragoza 50009, Spain

## Abstract

The reaction of the 11-vertex rhodathiaborane [8,8,8-(H)­(PPh_3_)_2_-3-(NC_5_H_5_)-*nido*-7,8-RhSB_9_H_10_] (**1**) with 1,2-bis­(diphenylphosphine)­benzene (dppbz) and (*S*)-(−)-2,2′-bis­(diphenylphosphino)-1,1′-binaphthyl (binap) affords [1,1-(η^2^-dppbz)-3-(NC_5_H_5_)-*closo*-1,2-RhSB_9_H_8_] (**2**) and [1,1-(η^2^-binap)-3-(NC_5_H_5_)-*closo*-1,2-RhSB_9_H_8_] (**3**). These 11-vertex *closo*-rhodathiaborane chelates result from PPh_3_ ligand substitution at the rhodium center and a *nido*-to-*closo* structural cluster transformation driven by H_2_ loss. Treating compounds **2** and **3** with triflic acid (TfOH) leads to the formation of cationic clusters **4** and **5**. The hydrons bind to the polyhedral clusters, acquiring hydride character and providing chemical nonrigidity that manifests through metal vertex-to-thiaborane *pseudo*-rotations and concomitant hydron tautomerisms. The resulting cations react with hydrogen to form mixtures of hydrons, hydrogen, and hydridorhodathiaboranes in equilibrium. The reaction products are the result of heterolytic cleavage of the H–H bond, with full participation of the clusters and the addition of hydrogen atoms to the cages. In these reactions, there is a conversion of electrons and hydrons into hydrogen, and hydrogen into hydride ligands, demonstrating that these boron-based metal compounds act as electron reservoirs, capable of promoting multielectron processes.

## Graphical abstract

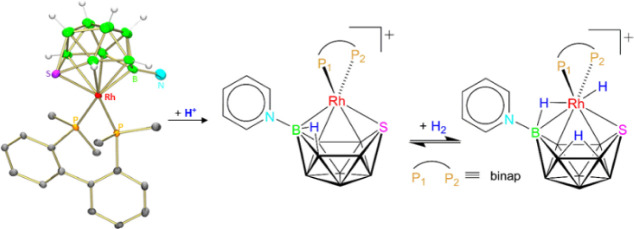

## Introduction

Metallaboranes and, more broadly, metallaheteroboranes (including the ubiquitous metallacarboranes) exhibit polyhedral structures supported by highly delocalized three-dimensional bonding.^1–11^ In these compounds, the borane/heteroborane fragment can be regarded as face-bound ligands, complementing the consideration of polyhedral boron-containing compounds as pure clusters.^12^ Both perspectives are valuable and illustrate the occasional uncertainty in classifying these metal–boron-based compounds. Thus, the conventional electron-counting rules devised by Wade, along with the structural classification introduced by Williams, provide convenient tools for the description and rationalization of a wide range of metallaboranes and metallaheteroboranes.^13,14^

In many cases, the metal-bound borane/heteroborane fragments in deltahedral metallaboranes and metallaheteroboranes can be related to classical ligands. This relationship is evident, for example, when the [C_2_B_9_H_11_]^2–^ carborane dianion is identified as a six-electron, η^5^-ligand, analogous to the [η^5^-C_5_H_5_]^−^ ligand.^1,2,15^

Both the cluster approach and the metal–ligand bound complex approach are consistent with the hybrid character of metallaboranes and metallaheteroboranes, which is reflected in their reaction chemistry.^16–18^ The ligand approach highlights the central concept in chemistry of altering the properties of a metal complex through ligand modification. Consequently, the design of new ligands is fundamental to virtually all areas of chemistry, including metal coordination, organometallic chemistry, materials science, and catalysis. Additionally, the concepts of metal–ligand collaboration and noninnocent ligands are well recognized in metal coordination chemistry,^19–22^ and have opened new ways for the activation of small molecules with applications in catalysis.^23–28^

However, it is not widely appreciated that metallaboranes and metallaheteroboranes combine the redox and coordinative flexibility of transition metal centers with the redox flexibility of boron clusters in their classical *closo*-*nido*-*arachno* structural transformations. Thus, these compounds possess characteristics that enable collaborative metal–borane/heteroborane processes, which can be useful for various applications. For instance, there is significant interest in developing new methods for converting hydrons and electrons into H_2_.^29^ In this context, the synthesis and study of transition metal complexes featuring reactive ligands capable of engaging in collaborative metal–ligand processes have provided new mechanisms and a better understanding of the elementary chemical reaction steps involved in H–H bond formation and cleavage.^30,31^

Our contributions to this reaction chemistry include research on rhodathiaboranes capable of activating hydrogen, with the participation of the entire cluster, through mechanisms characteristic of metal–ligand collaboration.^32,33^ Moreover, we have been able to tune and modify the reactivity of the cluster via modular chemistry by simply altering ligands at either the rhodium center, substituents on boron vertices, or both.^34^ In investigating new metallathiaboranes capable of activating small molecules, we discovered that protonation of some 11-vertex rhodathiaboranes increases their reactivity toward hydrogen, resulting in products of heterolytic H–H splitting and addition to the cluster, with structural transformations involving cooperative metal–thiaborane fragment mechanisms.

In this paper, we continue our research on the synthesis and study of rhodathiaboranes, focusing on developing new mechanisms of hydrogen activation that can be used in catalytic processes, such as asymmetric hydrogenations and/or the oxidation of hydrogen to hydrons and electrons. Building on our previous results, we modified the 11-vertex rhodathiaborane [8,8-(PPh_3_)_2_-*nido*-8,7-RhSB_9_H_10_] (**1**) by substituting the PPh_3_ ligands at the metal center with the chelating phosphines 1,2-bis­(diphenylphosphine)­benzene (dppbz) and with (*S*)-(−)-2,2′-bis­(diphenylphosphino)-1,1′-binaphthyl (binap), obtaining new compounds **2** and **3**. These polyhedral chelates have been characterized using spectroscopic and X-ray diffraction methods. In this study, their reaction chemistry with triflic acid and hydrogen has been researched, demonstrating that metallathiaboranes are well-suited for the systematic late-stage modular tuning of cluster electronics and sterics.

## Results and Discussion

### Synthesis and Characterization of 11-Vertex Neutral Dppbz- and Binap-Ligated Rhodathiaborane Chelates

The reactions of [8,8,8-(H)­(PPh_3_)_2_-3-(NC_5_H_5_)-*nido*-7,8-RhSB_9_H_10_] (**1**) with 1,2-bis­(diphenylphosphine)­benzene (dppbz) and (*S*)-(−)-2,2′-bis­(diphenylphosphino)-1,1′-binaphthyl (binap) in dichloromethane at reflux temperature lead to the substitution of the two monodentate triphenylphosphine ligands, affording the 11-vertex *closo*-rhodathiaboranes, [1,1-(η^2^-dppbz)-3-(NC_5_H_5_)-*closo*-1,2-RhSB_9_H_8_] (**2**) and [1,1-(η^2^-binap)-3-(NC_5_H_5_)-*closo*-1,2-RhSB_9_H_8_] (**3**) (Scheme 1).

**Scheme 1:**
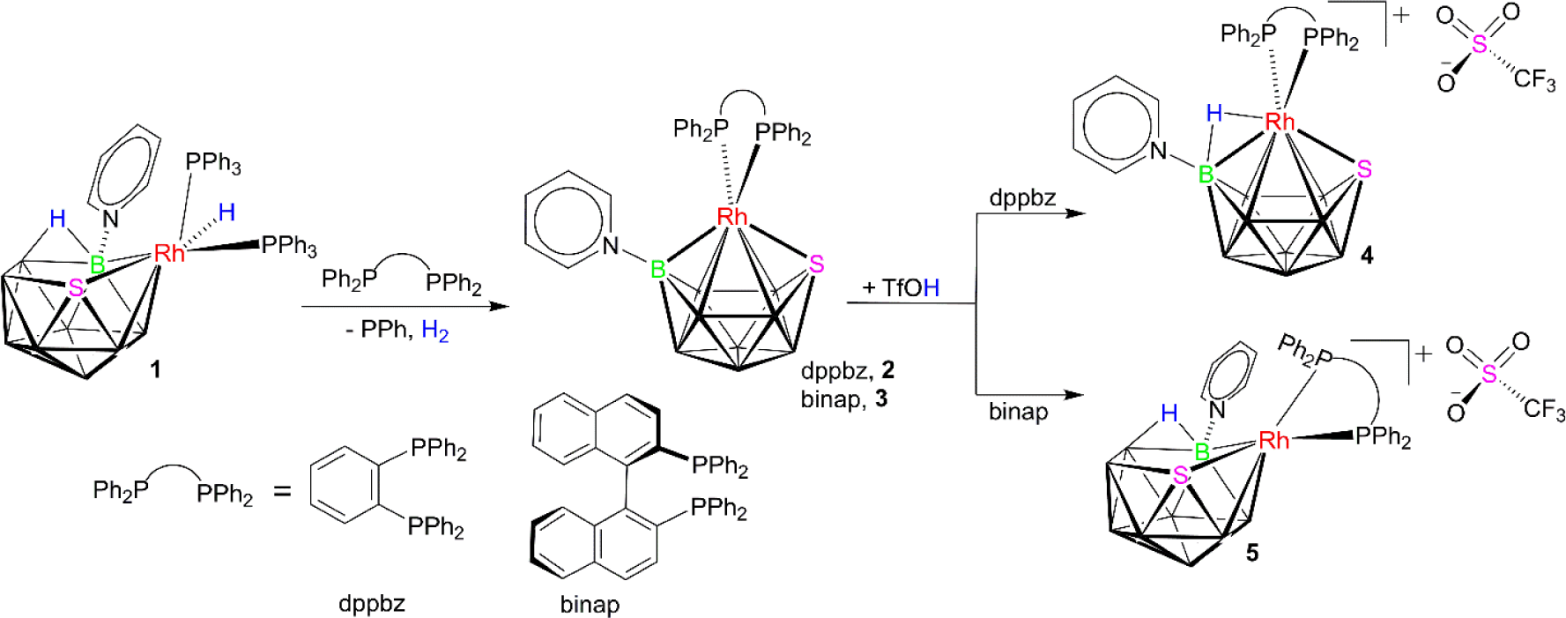
Synthesis of Compounds **2** and **3**, Simple Protonation to Form **4**, and Protonation and Rearrangement to Give **5**

This reactivity mirrors the previously documented reaction involving **1** and bis­(diphenylphosphino)­ethane (dppe),^35^ providing a convenient synthetic procedure for the preparation of 11-vertex pyridine-ligated *closo*-rhodathiaborane chelates. Under identical conditions, the reaction with dppe reaches completion within 16 hours. In contrast, the conversion with dppbz and binap requires a prolonged stirring period of 10 days under an argon atmosphere at reflux temperature in dichloromethane. This variation in reaction kinetics can be attributed to the greater steric hindrance and lower conformational flexibility of the chelating ligands dppbz and binap, which require more substantial rearrangements during the reactions compared to dppe. Additionally, dppe is a more basic and nucleophilic phosphine, which can be regarded as an electronic effect, typically resulting in faster reaction rates.

Consistent with these electronic and structural effects, it is noteworthy that the substitution reactions of the PPh_3_ ligands in hydridorhodathiaborane **1** with monodentate phosphines such as PMePh_2_, PMe_2_Ph, and PMe_3_ occur at room temperature, within minutes.^31^ The resulting hydridorhodathiaboranes remain stable at room temperature; however, it is necessary to heat the dichloromethane solutions of the monodentate phosphine-substituted clusters to 50 °C to facilitate dehydrogenation and the consequent *nido*-to-*closo* structural transformation.

From the aforementioned results, it can be inferred that the bidentate ligands dppbz and binap do not undergo a reaction with **1** at room temperature. Consequently, the formation of 11-vertex *nido*-hydridorhodathiaborane chelates, analogous to **1** (and to other monophosphine counterparts), is not achievable using the procedure reported herein. This limitation arises as the slow PPh_3_ substitution process occurs upon heating, which subsequently promotes H_2_ loss.

The crystal and molecular structures of **2** and **3** were determined by X-ray diffraction analysis. Selected interatomic distances and angles are provided in the captions of Figures 1 and 2 to illustrate the structure of the rhodathiaboranes. Both compounds have a molecular structure based on an octadecahedron with *C*_2v_ symmetry, which is expected for 11-vertex boron-based clusters that feature 11 + 1 (i.e., *n* + 1, where “*n*” is the number of vertices) skeletal electron pairs.^13,14^

**Figure 1:**
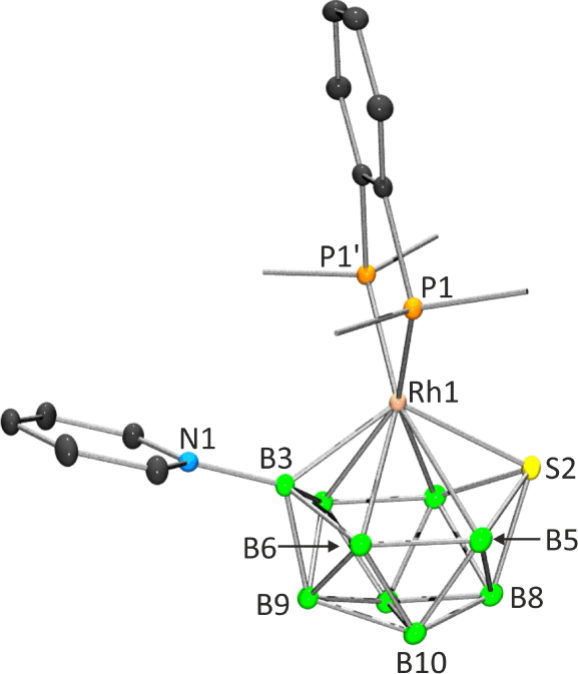
Single-crystal X-ray structures of compound **2**. Ellipsoids are shown at the 50% probability level. In the left view, only the *ipso*-carbon atoms on the phenyl rings of the dppbz ligand are included to aid clarity. Selected bond lengths (Å) and bond angles (deg): Rh1–P1/P2 2.2483(4), Rh1–S2 2.3824(6), Rh1–B3 2.101(3), Rh1–B4/B5 2.443(2), Rh1–B6/B7 2.3793(19), N1–B3 1.545(3), S2–B4/B5·1.947(2), S2–B8·1.981(3), B4/5–B8 1.915(3) (longest), B3–B6/7·1.713(3) (shortest); P1–Rh1–P2 85.85(2), P1–Rh1–S2 112.756(16), P1–Rh1–B3 110.10(5), S2–Rh1–B3 120.13(8), Rh1–B3–N1 131.42(17).

**Figure 2:**
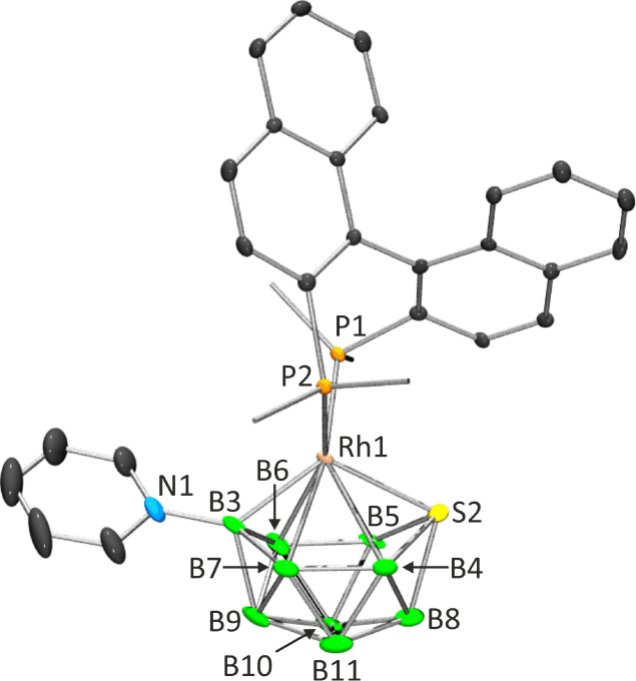
Single-crystal X-ray structures of compound **3**. Ellipsoids are shown at the 50% probability level. In the left view, only the *ipso*-carbon atoms on the phenyl rings of the binap ligand are included to aid clarity. Selected bond lengths (Å) and bond angles (deg): Rh1–P1 2.2862(14), Rh1–P2 2.3053(15), Rh1–S2 2.3882(15), Rh1–B3 2.111(7), Rh1–B4 2.462(7), Rh1–B5 2.458(7), Rh1–B6 2.344(7), Rh1–B7 2.421(8), N1–B3 1.552(9), S2–B4 1.939(8), S2–B5 1.931(7), S2–B8 1.974(8), B5–B8·1.914(12) (longest), B3–B6· 1.717(11) (shortest); P1–Rh1–P2 90.91(5), P1–Rh1–S2 100.40(5), P2–Rh1–S2 109.68(5), P1–Rh1–B3 122.3(2), P2–Rh1–B3 110.3(2), S2–Rh1–B3 119.4­(2), Rh1–B3–N1 134.0(5).

#### X-Ray Diffraction Analysis

The dppbz derivative, **2**, crystallizes in the orthorhombic system, specifically within the *Pnma* space group. In the unit cell, four molecules occupy special positions in the *a* plane. This plane precisely bisects the 11-vertex octadecahedral cluster, delineated by the {Rh1S2B8B9B3N1C14} vertices, into two crystallographically equivalent halves. Conversely, the binap-ligated counterpart, **3**, adopts a crystalline form within the triclinic system under the *P*1 space group. In this arrangement, one formula unit contains two rhodathiaborane clusters, four molecules of ClCH_2_–CH_2_Cl, and a molecule of CH_3_(CH_2_)_4_CH_3_. 1,2-Dichloroethane and hexane served as the solvent system used for crystallization, cocrystallizing with the boron-based cluster. Analyzing the Rh–P distances in **3** reveals notable distinctions; they measure 2.2862(14) and 2.3053(15) Å, significantly longer than the Rh–P distance of 2.2483(4) Å observed in the dppbz derivative, **2**. This discrepancy reflects the differing steric and electronic characteristics inherent to both chelating ligands.

It is noteworthy that the crystallographically characterized binap-ligated rhodium­(I) complexes (36 hits in the CCDC) showcase Rh–P distances spanning from 2.177(62) to 2.45(7) Å, with a mean value of 2.266(60) Å.^36^ This observation underlines that the Rh–P distances in **3** surpass the mean value. Across all complexes, we find that one of the Rh–P distances is significantly longer than the other. This asymmetry thus emerges as a distinctive structural characteristic inherent to binap-Rh­(I) chelates.

In contrast to the 36 Rh­(I)-binap complexes documented in the CCDC, only eight dppbz-ligated rhodium complexes have been structurally characterized. Among them, [RhCl­(IPr)­(dppbz)] and [Rh­(dppbz)_2_]^+^ represent the sole Rh­(I) complexes, while the remaining five are Rh­(III) complexes. Analyzing the Rh–P distances in these dppbz derivatives reveals a range between 2.179(67) and 2.362(67) Å, with the largest distance observed in the bis-dppbz Rh­(I) cation. The mean distance of 2.292(53) Å exceeds the Rh–P length of 2.2483(4) Å, observed in the dppbz derivative, **2**.

In compound **2**, the dppbz ligand coordinates to the rhodium atom, forming a planar five-membered ring chelate that is perpendicular to the S2Rh1B3 plane, as depicted in Figure 1. Additionally, the chiral binap phosphine in **3** forms a seven-membered ring, which deviates from the ideal λ-skew conformation, as illustrated in Figure 2. The *exo*-polyhedral P1Rh1P2 plane exhibits a slight twist with respect to the S2Rh1B3 cluster plane, forming an angle of 82°. This conformation resembles that found in the binap-ligated rhodium­(I) complex, [Rh­(*R*-binap)­(COD)]^+^.^37^

As for the P1–Rh1–P2 bite angles, they measure 85.85(2)° for dppbz-ligated **2** and 90.91(5)° for binap-ligated **3**. These values fall within the range found for other crystallographically characterized dppbz and binap complexes, such as [Rh­(dppbz)_2_]^+^,^38^ [RhCl­(dppbz)­(IPr)],^39^ [Rh­(binap)­(NH_3_)_2_]^+^, and [Rh­(binap)­(COD)]^+^.^37,40^

This analysis reveals, therefore, that the chelates have different rhodium-to-cage conformations of the Rh­(P–P) unit relative to the SB9 ligand, where P–P = dppbz or binap. Moreover, the Rh1–B4 and Rh1–B5 distances (2.443(2) in **2**; 2.462(7) and 2.458(7) in **3**) are longer than the Rh1–B6 and Rh1–B7 lengths (2.3793(19) in **2**; 2.344(7) and 2.421(8) in **3**), with the Rh1–B3 distance being significantly shorter at 2.101(3) and 2.111(7) in **2** and **3**, respectively. This distance pattern reveals a slip of the metal fragment toward the B6B3B7 side, which is more evident in the binap-ligated cluster that shows a significant decrease in the Rh1–B6 length.

This cluster asymmetry, in the solid state, suggests that the steric bulk of the dppbz and binap frameworks has a substantial effect on the geometry of the interaction between the bidentate ligands and the {Rh­(SB_9_H_8_)} moiety. However, we cannot ignore the influence of the sulfur vertex on the Rh-to-thiaborane bonding, providing two formal pairs of electrons to the cluster framework.

#### NMR Characterization

Table 1 presents the ^11^B and ^1^H NMR spectroscopic data for [1,1-(PPh_3_)_2_-3-(NC_5_H_5_)-*closo*-1,2-RhSB_9_H_8_] (**(PPh_3_)_2_Rh-TB**) and compounds **2** and **3**. Thus, the ^11^B–{^1^H} NMR spectra of these 11-vertex *closo*-rhodathiaboranes exhibit six peaks in a 1:1:2:1:2:2 relative intensity ratio within the range of δ­(^11^B) +55 to −33 ppm, indicative of *C*_s_ symmetry in solution. This spectroscopic pattern is consistent with the Rh-to-{SB_9_H_8_} conformation found in the crystal structure of the dppbz derivative, characterized by a plane of symmetry, as discussed above and illustrated in Figure 1.

**Table 1:** ^11^B and ^1^H NMR Data for [1,1-(PPh_3_)_2_-3-(NC_5_H_5_)-*closo*-1,2-RhSB_9_H_8_] (**(PPh_3_)_2_Rh-TB**), [1,1-(dppbz)-3-(NC_5_H_5_)-*closo*-1,2-RhSB_9_H_8_] (**2**), and [1,1-(binap)-3-(NC_5_H_5_)-*closo*-1,2-RhSB_9_H_8_] (**3**) in CD_2_Cl_2_

	(**(PPh_3_)_2_Rh-TB**)^[^ ^a^ ^]^		**2** ^[^ ^a^ ^]^		**3** ^[^ ^a^ ^]^	
	^11^B	^1^H	^11^B	^1^H	^11^B	^1^H
B3	+54.6	–	+53.9	–	+54.0	–
B9	+27.3	+4.9	+26.9	+4.03	+25.9	+3.97
B4	–0.5 (2B)	+1.27	+0.1 (2B)	+1.88	–0.2	+2.08
B5					–1.3	+1.10
B8	–15.2	+2.16	–12.6	+2.52	–15.5	+2.20
B7	–24.2 (2B)	–0.22	–23.7 (2B)	+0.12	–22.6	–0.86
B6					–27.5	+0.14
B10	–30.4 (2B)	–0.27	–32.1 (2B)	–0.14	–29.7	–0.12
B11					–30.7	–0.24

a Assignments based on ^1^H–{^11^B} selective experiments and comparison with reported 11-vertex pyridine-ligated *closo*-rhodathiaboranes.^34,35^

The introduction of the chiral (*R*)-(+)-binap ligand disrupts the *C*_s_ symmetry of the 11-vertex octadecahedral cluster, resulting in a ^11^B NMR spectrum that features nine peaks. Notably, the pyridine-substituted boron resonates at the highest frequency, typically between δ­(^11^B) +55 and +53 ppm, positioned at approximately 27 ppm lowfield from the second highest deshielded resonance around +26 ppm.

These spectral patterns closely resemble those of previously reported rhodathiaborane pyridine adducts, facilitating their identification through ^11^B NMR spectroscopy.^34,35^ Consistent with the ^11^B NMR data, the ^1^H–{^11^B} NMR spectra of compound **2** exhibit five proton resonances between δ­(^11^B) +4.03 and −0.14 ppm, confidently assignable to the boron-bound terminal hydrogen atoms. The expected 1:2:1:2:2 relative intensity pattern further reveals the presence of a plane of symmetry in the cluster. Similarly, the asymmetry of compound **3** is confirmed by the presence of eight broad peaks in the ^1^H–{^11^B} resonances within the range of δ­(^1^H) +3.97 and −0.24 ppm.

Notably, the proton resonance at the highest frequency corresponds to the boron vertex at the 9-position in the cluster, which is adjacent to the pyridine substituent and thus susceptible to deshielding effects due to the aromatic current of the pyridine ring. Alternatively, this significant lowfield shift of the B9-bound terminal hydrogen atoms in **2** and **3** may be attributed to the antipodal effect of the sulfur atom.^41^ The ^1^H NMR spectra also indicate the presence of the rhodium-bound dppbz and binap ligands in compounds **2** and **3**, respectively, as well as the B3-bound pyridine substituent. Interestingly, the peak of the *ortho*-pyridine protons appears at δ­(^1^H) +9.30 ppm for **3**, which is the highest frequency resonance in the spectrum, whereas for **2**, the *ortho*-pyridine protons resonate at +7.57 ppm. This represents a 1.73 ppm downfield shift compared to the binap-ligated counterpart, reflecting the different steric and electronic characteristics between these chelating phosphines.

^31^P NMR spectra support the molecular structures of both rhodathiaboranes, with the presence of a doublet at δ­(^31^P) +68.4 ppm (151 Hz) for **2**, and an apparent triplet of doublets for **3**, which reveals two phosphorus resonances with second-order effects.

The asymmetry observed in the ^11^B, ^1^H, and ^31^P–{^1^H} spectra of compound **3** is consistent with the prochiral nature of the {η^6^-SB_9_H_8_(NC_5_H_5_)} fragment, which exhibits a mirror plane. The interaction with the chiral Rh­(binap) group induces asymmetry in the thiaborane moiety, causing B6/B7, B4/B5, and B8/B10 to remain nonequivalent throughout a complete rotation relative to the metal vertex. Consequently, we are unable to ascertain the presence of free rotation of the pyridine-ligated η^6^-thiaborane ligands in compounds **2** and **3**. In metal complexes such as [Rh­(η^5^-C_5_Me_5_)­(dppbz)] and [Rh­(binap­((η^6^-C_6_H_6_)]^+^, however, it is generally accepted that the arene rings undergo free rotation.^42,43^

### Reactions with Triflic Acid

As illustrated in Scheme 1, the treatment of the 11-vertex neutral *closo*-rhodathiaboranes, **2** and **3**, with triflic acid (TfOH) afforded the corresponding polyhedral cationic derivatives represented by the formulas [(dppbz)­(NC_5_H_5_)­RhSB_9_H_9_]­[TfO] (**4**) and [(binap)­(NC_5_H_5_)­RhSB_9_H_9_]­[TfO] (**5**). These reactions were conducted on a small scale in NMR tubes. Subsequently, the polyhedral cations were thoroughly characterized using multinuclear NMR spectroscopy, mass spectrometry, and elemental analysis.

The ^11^B NMR spectrum of the dppbz rhodathiaborane **4** closely resembles that of the parent cluster **2**, with the only notable difference being a slight deshielding of the signals in the protonated cluster. The most significant shifts toward higher frequencies are observed at δ­(^11^B) –23.7 and –32.1 ppm, corresponding to the B6,7 and B10,11 resonances, respectively (see Figure S1). These data clearly indicate that the 11-vertex *closo*-octadecahedral structure, as described for **2**, is retained in solution over the time scale of the NMR experiment. At room temperature, the ^1^H NMR spectrum of **4** also displays a high-frequency shift in the proton resonances. The most pronounced deshieldings occur at 0.72 and 0.93 ppm, corresponding to the terminal hydrogen atoms bonded to the B6,7 and B10,11 vertices, respectively. This observation mirrors the effect found in the ^11^B NMR spectrum. In addition, the ^1^H–{^11^B} spectrum of compound **4** shows a distinct peak at δ_H_ −6.31 ppm, exhibiting the pattern of an overlapped triplet of doublets. This signal undergoes considerable broadening in the conventional ^1^H NMR spectrum, indicating coupling with ^11^B nuclei (see Figures S2–S4). This observation, coupled with the multiplicity of the peak, provides evidence for protonation occurring along the Rh1–B3 edge, as depicted in Scheme 1.

The reactivity of the dppbz derivative **2** with triflic acid mirrors that observed in previously reported bis-phosphine *isonido*-rhodathiaboranes, represented by the general formula [1,1-(PR_3_)_2_-3-(NC_5_H_5_)-1,2-RhSB_9_H_8_], where PR_3_ = PPh_3_; PMe_2_Ph; PMe_3_; PPh_3_, PMe_3_; PPh_3_, PMe_2_Ph.^41^ Therefore, the appearance of low-frequency multiplets in the ^1^H NMR spectra within the range of δ_H_ −5.00 to −7.00 ppm upon protonation of the neutral clusters serves as a diagnostic marker for the formation of polyhedral cations that maintain an 11-vertex *isonido*/*closo*-octadecahedral structure.

The ^31^P–{^1^H} NMR spectrum of compound **4** features a doublet, which indicates the retention of the symmetry plane observed in the parent rhodathiaborane **2**. This finding corroborates the hypothesis that, within the time frame of the NMR experiment, the proton is situated along the Rh1–B3 edge, as discussed above and illustrated in Scheme 1. The resonance of the cationic species suffers a low-frequency shift and a reduction of 46 Hz in the ^1^ *J*_P,Rh_ coupling constant compared to its neutral counterpart. This decrease in the coupling constant supports the formal oxidation of the metal center, consistent with the formation of a less electron-rich rhodium center (depicted in Figure 3), which reduces the back-donation to the π-acidic phosphine chelate. This spectroscopic behavior resembles that found for previously reported Cp*-ligated complex [Rh­(η^5^-C_5_Me_5_)­(dppbz)] and [1,1-(PR_3_)_2_-3-(NC_5_H_5_)-1,2-RhSB_9_H_8_] rhodathiaboranes.^42,44^ Unlike the protonated dppbz rhodathiaborane **4**, the binap system **5** exhibits a distinctly altered ^11^B NMR spectrum compared to its neutral precursor **3**. Notably, the ^11^B spectrum experiences an overall shielding effect, primarily characterized by a 28 ppm shift towards lower frequencies of the pyridine-substituted B3 (refer to Figure S5). This observation suggests a significant structural modification of the parent eleven-vertex *closo*-octadecahedral cluster framework upon the reaction with triflic acid.

**Figure 3:**
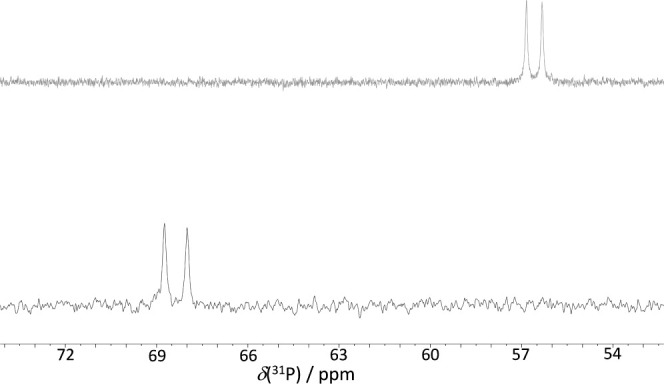
^31^P–{^1^H} NMR (202 MHz) of **4** (top, CD_2_Cl_2_, ^1^ *J*_P,Rh_ = 105 Hz) and **2** (bottom, CD_2_Cl_2_, ^1^ *J*_P,Rh_ = 151 Hz).

At room temperature, the ^31^P–{^1^H} spectrum reveals two well-defined doublet of doublets peaks at δ­(^31^P) +35.6 and +15.7 ppm (Figure S6). The peak at a lower frequency is significantly broader, accompanied by a ^1^ *J*_P,Rh_ coupling constant of 120 Hz, which is 22 Hz smaller than that of the highest frequency peak. The substantial chemical shift disparity and the peak width observed indicate a distinct environment for the phosphorus atoms within the protonated structure of system **5**. The broadness of the lower frequency peak suggests the presence of ^2^ *J*_P,B_ coupling, consistent with a *trans* disposition to either a boron vertex or a B–B edge. Conversely, the higher frequency doublet of doublets likely originates from phosphorus nuclei positioned *trans* to the sulfur vertex.

It is worth noting that simple protonation of **3** along Rh1–B3, akin to the dppbz derivative **4**, would result in a *closo*-structure featuring two chemically distinct phosphorus atoms. However, these phosphorus atoms would likely occupy similar environments, leading to two closely spaced ^31^P resonances in the ^31^P–{^1^H} NMR spectrum, resembling those observed for the parent cluster **3**. Thus, the ^31^P–{^1^H} NMR spectrum corroborates the ^11^B NMR data, indicating a structural alteration in the octadecahedral 11-vertex binap-ligated rhodathiaborane upon reaction with triflic acid.

In contrast to the aforementioned observations, the ^1^H–{^11^B} spectrum displays a 1:1:2:1:1:1:1:1 relative intensity pattern for the hydrogen atoms bound to boron, spanning from δ­(^1^H) +4.5 and −0.19 ppm, reminiscent of parent compound **3**. However, within this spectral range, the signals experience an approximate 1 ppm shift toward higher frequencies and notable peak broadening in the triflic-reaction system **5** compared to the neutral parent compound **3** (see Figure S7). In contrast, the peak assigned to the *ortho*-pyridine hydrogen atom suffers a low-frequency shift of 0.62 ppm.

Furthermore, the ^1^H–{^11^B} spectrum of **5** presents multiple signals with varying relative intensity ratios within the δ­(^1^H) −1.5 to −11.5 ppm range (refer to Figure S8). Remarkably, some of these signals, despite their low intensity, exhibit broadening in the conventional ^1^H NMR spectrum, while others remain unaffected, indicating the formation of new minor species that feature B–H–B, Rh–H–B, and Rh–H boding interactions. However, notably absent is a major single resonance in the negative region that could provide evidence for protonation along the Rh1–B3 edge.

#### Variable Temperature (VT) NMR Studies

As the temperature decreases within the triflic acid reaction system **5**, the two doublets of doublets observed in the ^31^P–{^1^H} NMR spectrum experience significant broadening at 223 K, only to return to their initial room temperature configuration at 183 K. This transition occurs without notable alterations in either the chemical shifts or the coupling constants, beyond the anticipated effects induced by temperature changes (Figure 4).

**Figure 4:**
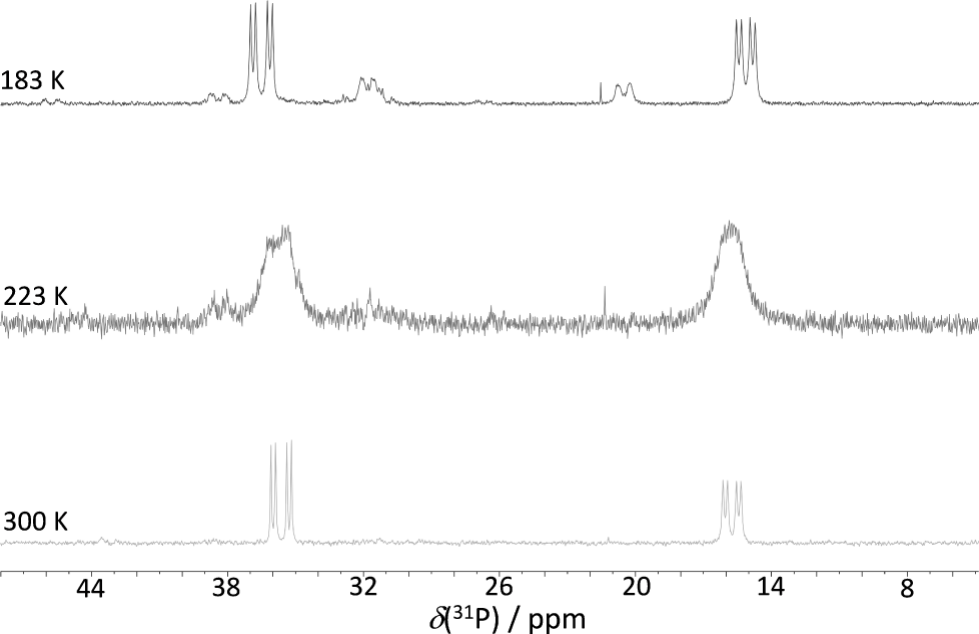
^31^P–{^1^H} NMR (202 MHz) of **5** at different temperatures.

At lower temperatures, the spectrum reveals the emergence of two broad doublets at δ­(^31^P) +31.8 and +20.5 ppm, in a 1:0.25 relative intensity ratio. Additionally, two sets of low-intensity broad doublets are detected at +38.5 and +31.4 ppm, with the latter overlapping with the peak at +31.8 ppm. Furthermore, signals are present at +45.8 and +26.7 ppm, with intensities two orders of magnitude smaller than the primary peaks.

Concomitant to the changes observed in the VT ^31^P–{^1^H} NMR spectra, the proton resonances that contribute to a broad peak around δ­(^1^H) −4.4 ppm, attributed to B–H–B hydrogen atoms, increase in intensity to match that of the binap ligand. Consequently, they transition to a predominant species. Moreover, the signals spanning from −7.4 to −11.5 ppm, corresponding to Rh–H–B and Rh–H linkages, also experience an intensity upsurge compared to that of the phosphine ligand. However, their relative intensity in relation to the primary signal at −4.4 ppm diminishes (refer to Figure S8).

These NMR data suggest that the protonation of cluster **3** leads to the formation of a chemically nonrigid system, combining intramolecular fluxional behavior with intermolecular equilibria. In this context, we have previously reported that treating [1,1-(η^2^-dppe)-3-(NC_5_H_5_)-*closo*-1,2-RhSB_9_H_8_], the counterpart of **2** and **3**, with triflic acid results in a cationic cluster exhibiting a *nido*-structure in the solid state, conveniently formulated as [8,8-(η^2^-dppe)-9-(NC_5_H_5_)-*nido*-8,7-RhSB_9_H_9_]^+^. This 11-vertex dppe-ligated cation features a fluxional process in solution, mechanistically involving a shift and half-rotation of the {Rh­(dppe)} vertex above the six-membered face of the {SB_9_H_8_(NC_5_H_5_)} moiety. This rotation is coupled to the reverse shift of the B–H–B hydrogen atom situated on the pentagonal open face of the 11-vertex *nido*-cluster. Consequently, during the NMR experiment, the two enantiomeric forms of the *nido*-cluster interchange via this mechanism.^35^

At lower temperatures, the *nido*-cluster’s lifespan extends sufficiently to manifest as a distinct species, which coexists in equilibrium with its 11-vertex isomer *closo*-isomer. This *closo*-tautomer, observable only at reduced temperatures, exhibits a broad low-frequency peak approximately at δ­(^1^H) −7 ppm, indicative of an Rh–H–B linkage along the Rh1–B3 edge within the 11-vertex octadecahedral cage. The overall fluxional process enables the observation of the *closo*-isomer as a transitional intermediate between the *nido*-enantiomers.^35^

We postulate that the binap-ligated system **5** undergoes a similar fluxional process, with the {Rh­(binap)} moiety *pseudo*-rotating back and forth around the hexagonal face of the thiaborane fragment, as depicted in Scheme 2. Nonetheless, a detailed analysis of the NMR data reveals additional insights, as described below.

**Scheme 2:**
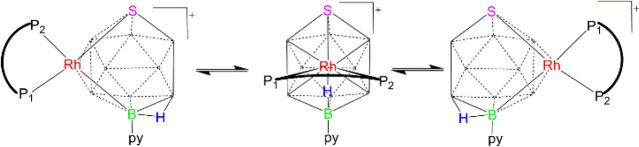
Proposed {Rh­(binap)}-Vertex *pseudo*-Rotation and Proton Tautomerism Mechanism for the Fast Exchange of *nido*-Diastereomers in [(Binap)­RhSB_9_H_9_(NC_5_H_5_)]^+^ (**5**)

Therefore, if we consider the *closo*-cation as the reference point, then the opening of its deltahedral structure would yield two *nido*-diastereomers (Scheme 2). In the event that these isomers possessed sufficient lifetime to manifest as distinct species, we would expect to find four doublets of doublets in the ^31^P–{^1^H} spectrum, indicative of two different phosphorus environments per diastereomer. However, both at room and low temperatures, only two major doublets of doublets are evident, implying that the vertex *pseudo*-rotation and proton tautomerism of the B–H–B hydrogen atom occur too rapidly to resolve both isomers within the time scale of the NMR experiment. Consequently, we witness the outcome of this rapid intramolecular fluxional process, which effectively renders the phosphorus nuclei of the chiral binap ligand equivalent in pairs (Scheme 2).

In addition, the VT NMR data also reveal the generation of a compound mixture containing Rh–H bonds. These hydridorhodathiaboranes exist in equilibrium with the nonrigid *nido*-species, which dominate as the major component, while their concentration rises with decreasing temperature. As a result, the ^31^P–{^1^H} MNR spectrum at 183 K displays newly emerged broad doublets, as explained earlier (see Figure 4).

This fluxional behavior of the cationic dppe- and the binap-clusters contrasts with the structural stability found in the dppbz counterpart **4**, where the proton remains along the Rh1–B3 edge, and the {Rh­(dppbz)} metal vertex does not migrate around the hexagonal boat-type thiaborane face. This difference in the cluster fluxionality aligns with the rigid, planar structure of the dppbz ligand, which results in higher energy barriers for intramolecular prototopic rearrangements. In contrast, the dppe and binap ligands exhibit a more flexible nature, leading to more dynamic metallathiaboranes.

The study of the reactivity of the cationic systems **4** and **5** with hydrogen, as outlined, offers a broader perspective that facilitates the comprehension of the mechanism underlying the formation of these hydride-ligated rhodathiaboranes.

### Hydrogen Reactivity

Previous studies have showcased the reactivity of both cationic, 11-vertex, chemically nonrigid [(η^2^-dppe)­RhSB_9_H_9_(NC_5_H_5_)]^+^ and bis-monodentate-ligated counterparts [(PR_3_)­RhSB_9_H_9_(NC_5_H_5_)]^+^, with dihydrogen.^30,32,41^ This reactivity causes heterolytic cleavage of the H–H bond, leading to subsequent addition to the cluster and the formation of hydride-ligated rhodathiaboranes. In contrast, the neutral parent clusters either exhibit no reaction with H_2_ or do so at slow rates.^34^ The reactivity observed in this family of 11-vertex cationic rhodathiaboranes, formed through simple protonation, represents a notable example of proton-assisted hydrogen activation.

Expanding on the reaction chemistry of cationic rhodathiaboranes, we investigated the reactions of systems **4** and **5** with hydrogen. These reactions were conducted on a small scale in quick-pressure valve NMR tubes, which were charged with either the dppbz- or binap-ligated clusters, **2** and **3**. The compounds were dissolved in deuterated dichloromethane and treated with stoichiometric amounts of triflic acid, leading to the *in situ* generation of the cationic species **4** and **5**. Subsequently, these reaction mixtures were subjected to an atmosphere of hydrogen and studied via multielement NMR spectroscopy, yielding the following results.

#### Dppbz-Ligated System

The ^11^B–{^1^H} NMR spectrum resulting from the reaction between compound **4** and H_2_ displays signals attributed to the parent polyhedral cation, alongside new signals appearing at δ­(^11^B) +16.4, +5.3, −11.8, −16.2, −19.1, −23.1, and −24.4 ppm. Conversely, the ^1^H–{^11^B} spectrum exhibits seven signals of varying intensities in the negative region. Notably, the peaks at δ­(^1^H) −3.30, −3.96, −5.75, −6.36 (br. multiplet assigned to **4**), and −6.50 ppm exhibit broadening in the conventional ^1^H spectrum, indicative of ^*n*^ *J*_B,H_ couplings (see Figures S9 and S10). In contrast, the broadening observed for the hydride-like resonance at −10.57 ppm (apparent quintet, 0.3 H, Rh–H) is subtler, maintaining the shape of a very broad *pseudo*-quintet. Meanwhile, the low-frequency multiplet at δ­(^1^H) −10.76 (td, 1 H, Rh–H) ppm does not demonstrate coupling to ^11^B nuclei (see Figure S11).

Based on these NMR data, we propose that the reaction of the cationic intermediate **4** introduces two hydrogen atoms into its polyhedral boron cage, resulting in two 11-vertex *nido*-hydridorhodathiaborane isomers, in a 1:0.3 relative intensity ratio. The observation that the lowest-frequency triplet of doublets remains well-resolved in the conventional ^1^H NMR spectrum, without coupling to boron nuclei, suggests that this hydride ligand is positioned *trans* to the sulfur vertex of the {SB_9_H_8_}-fragment. Furthermore, the coupling constant of 60 Hz for the doublet of doublets at δ­(^1^H) −6.50 ppm, in the ^1^H–{^11^B} spectrum, indicates strong ^2^ *J*_H,P_ coupling, consistent with a hydrogen atom situated *trans* to a phosphorus nucleus. This finding, coupled with the significant broadening of this peak in the ^1^H–{^11^B­(off)} and ^1^H–{^31^P} spectra (Figure S11), indicates the presence of an Rh–H–B bridging hydrogen atom. Additionally, the singlet observed at −3.33 ppm likely corresponds to hydrogen atom binding to a B–B edge.

Overall, our analysis suggests the formation of an 11-vertex *nido*-cluster featuring an {RhSB_3_}-pentagonal face that accommodates two bridging hydrogen atoms. The {RhH_2_(dppbz)}-to-{SB_9_H_8_(NC_5_H_5_)} configuration, corresponding to isomer **6a** as depicted in Scheme 3, is consistent with these findings.

**Scheme 3:**
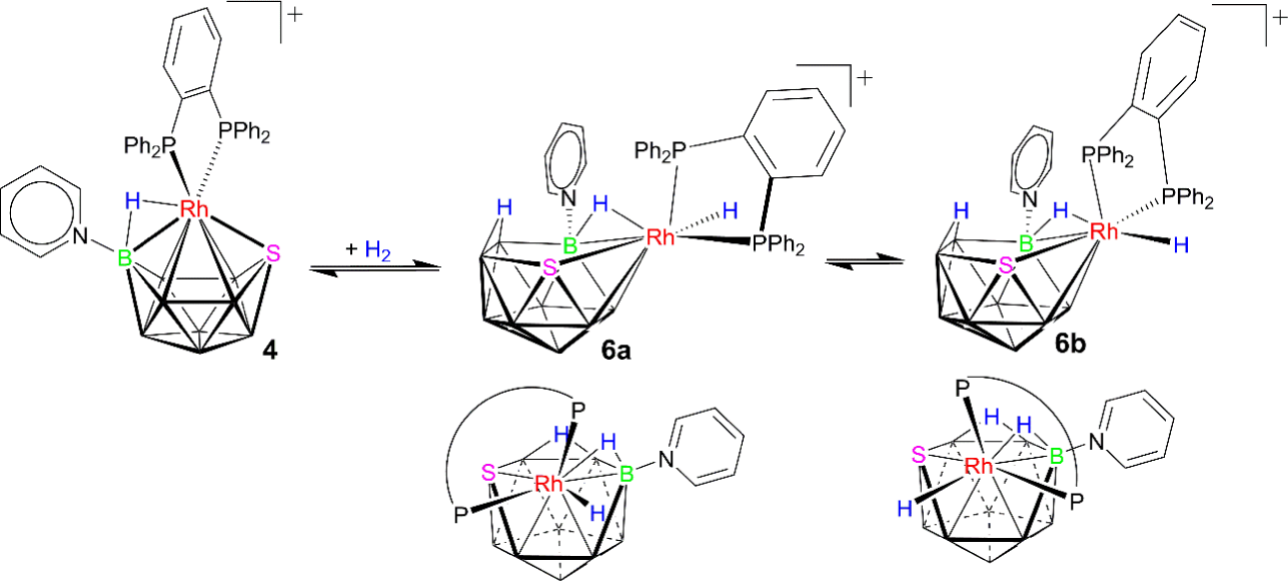
Reaction of Cationic [1,1-(dppbz)-3-(NC_5_H_5_)-*closo*-1,2-RhSB_9_H_9_] (**4**) with Hydrogen to Give Two Hydridorhodathiaborane Conformers, **6a** (Major) and **6b** (Minor)

Similarly, the spatial arrangement of the metal-to-thiaborane configuration of the minor isomer can be deduced by examining the three remaining low-frequency resonances. The broad *pseudo*-quintet at δ­(^1^H) −10.57 ppm, as commented above, displays a minor ^*n*^ *J*_B,H_ coupling in the ^1^H–{^11^B} spectrum, suggesting a potential *trans* position of the Rh–H bond to either a boron vertex or a B–B edge. Additionally, the broad multiplet at −5.75 ppm in the ^1^H–{^11^B} spectrum is indicative of the coupling of a hydride-like ligand with several nuclei, displaying similar coupling constants. Notably, this resonance distinctly couples with boron nuclei, leading us to attribute it to an Rh–H–B bridging hydrogen atom.

In addition, the [^1^H–^1^H–{^11^B}]–COSY spectrum of the reaction mixture **4** under H_2_ shows a cross-peak correlation between the signals at δ­(^1^H) −5.75 and −10.57 ppm. This indicates that these hydride-like resonances belong to the same rhodathiaborane (Figure S12).

Lastly, the third low-frequency resonance at −3.96 ppm is assigned to a B–H–B interaction. Consequently, the low-intensity isomer is characterized by the configuration of the metal vertex in relation to the {SB_9_H_8_(NC_5_H_5_)}-fragment, which is depicted as **6b** in Scheme 3.

It should be noted that the two isomers, **6a** and **6b**, are capable of *a priori* interconversion through a simple {Rh-dppbz}-to-{SB_9_H_8_NC_5_H_5_} rotation. In this context, the [^1^H–^1^H]–NOESY spectrum at room temperature shows off-diagonal peaks between the Rh–H–B proton signals at −6.50 ppm and the Rh–H lowest frequency peaks (see Figure S13).

Additionally, crossed-peak correlations are noted between the peak of free hydrogen at +4.50 ppm and all the hydride-like resonances between −6 and −11 ppm. These off-diagonal peaks have the same phase as the diagonal signals, indicating the chemical exchange of these hydrogen atoms during the NMR experiment’s time scale.

According to the presence of two new cationic *nido*-hydridorhodathiaboranes, the ^31^P–{^1^H} spectrum exhibits two sets of doublets with equal intensity. These signals are observed at δ­(^31^P) +66.2 and +59.2 ppm, along with another pair at +65.3 and +56.1 ppm. The relative intensity ratio between both pairs of signals is approximately 1:0.20, aligning closely with the proportion observed in the ^1^H NMR spectrum for the two species. The spectrum also shows the doublet at +56.6 ppm assigned to cationic **4** (Figure S14).

To this spectroscopic behavior, we should add the fact that the release of hydrogen from the NMR tube results in the regeneration of the parent cationic cluster **4**, demonstrating that, as illustrated in Scheme 3, all the species are in equilibrium in solution, undergoing reversible proton-assisted hydrogen transfer chemistry. To further validate the molecular structure of the newly characterized hydride-ligated clusters, attempts were made to grow single crystals within a quick pressure valve NMR tube. This was achieved by overlaying the CD_2_Cl_2_ solution of compound **4** with hexane while maintaining a hydrogen atmosphere. After several days, a crystal was isolated and subjected to X-ray diffraction analysis. The obtained data unveiled the formation of [1,1-(η^2^-dppbz)-3-(NC_5_H_5_)-6-(TfO)-*closo*-1,2-RhSB_9_H_7_)] (**7**).

#### Crystal Structure of 7

The molecular structure of this triflate-substituted derivative is illustrated in Figure 5, with the accompanying caption providing selected intramolecular distances and angles. The cluster presents an 11-vertex octadecahedral *closo*-configuration reminiscent of its unsubstituted dppbz counterpart **2**. Notably, the cluster exhibits a long Rh1–B4 distance of 2.468(5) Å, nearing the upper limit of 2.5 Å typically associated with bonding. Likewise, the binap derivative **3** also features an elongation in the Rh–B4/B5 links flanking the Rh1–S2 connection, a structural trait commonly observed in *closo* 11-vertex metal–boron polyhedral compounds. Should the elongation progress further, approaching or surpassing the 2.5 Å limit, the triangular face transitions into a *pseudo*-square, representing an intermediate state along the structural continuum from *closo* to *nido* configurations (see Schemes 1–3). The B6–O1 distance measures 1.514(5) Å, slightly exceeding the mean value of 1.469 Å observed across six triflate-ligated polyhedral boron-containing compounds deposited in the Cambridge Structural Database (CSD).^33^ Conversely, the S–O1 length at 1.520(3) Å closely approximates the mean value of 1.524 Å for this B–O–S bridging linkage observed in substituted decaborane and carborane clusters analyzed crystallographically.^45–50^

**Figure 5:**
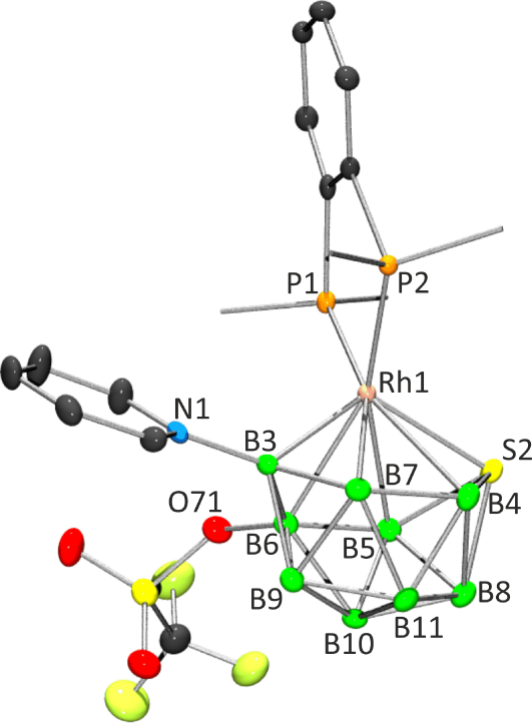
Single-crystal X-ray structures of compound **7**. Ellipsoids are shown at the 50% probability level. In the left view, only the *ipso*-carbon atoms on the phenyl rings of the dppbz ligand are included to aid clarity. Selected bond lengths (Å) and bond angles (deg): Rh1–P1 2.2807(10), Rh1–P2 2.2359(10), Rh1–S2 2.3824(10), Rh1–B3 2.104(4), Rh1–B4 2.468(5), Rh–B5 2.436(5), Rh1–B6 2.364(5), Rh–B7 2.365(4), N1–B3 1.532(5), S2–B4 1.937(5), S2–B5· 1.938(5), S2–B8·1.981(5), B4–B8 1.907(7), B5–B8 1.917(7) (longest), B3–B6·1.696(6) (shortest), B3–B7 1.702(6), B6–O1 1.514(5), S–O1 1.520(3), S–O2 1.423(4), S–O3 1.423(4), S–C 1.854(5), C–F (mean) 1.312(6); P1–Rh1–P2 85.51(4), P1–Rh1–S2 109.73(4), P2–Rh1–S2 115.78(4), P1–Rh1–B3 116.38(12), P2–Rh1–B3 105.25(12), S2–Rh1–B3 119.36(12), Rh1–B3–N1 131.1(3).

The introduction of the CF_3_–SO_3_ group breaks the symmetry present in the parent cluster **2**, and the dppbz ligand tilts slightly in derivative **7** to mitigate steric hindrance from the triflate substituent. Similarly, the pyridine ligand exhibits a minor rotation around the B3–N bond to avoid the TfO group (Figure 5).

The characterization of triflate-substituted 11-vertex rhodathiaborane **7** opens an interesting perspective on the reaction chemistry of dppbz and binap chelates. It demonstrates that, in 11-vertex metallathiaborane pyridine adducts, electrophilic substitutions can occur through protonation and dehydrogenation processes.^51–53^

#### Binap-Ligated System

The reaction involving the protonated binap-ligated system **5** and hydrogen also yields a mixture of species, reflected in the ^11^B–{^1^H} NMR spectrum, showcasing approximately 18 peaks within the range of δ­(^11^B) +16.0 and −29.5 ppm (Figure S15). Both chelating compounds exhibit a consecutive low-frequency shift of the ^11^B resonances compared to the neutral *closo*-clusters **2** and **3**, extending through the protonated species **4** and **5** to the final hydrogen reaction mixtures, exemplified by the proposed isomeric hydridorhodathiaboranes, **6a** and **6b**.

The NMR data of these hydrogen/cationic rhodathiaborane reaction mixtures resembles those spectra observed for bis-phosphine-ligated pyridine adducts, characterized by a general formulation [8,8,8-(H)­(PR_3_)_2_-9-(NC_5_H_5_)-*nido*-8,7-RhSB_9_H_10_]^+^ cations, where PR_3_ represents ligands such as PPh_3_, PMePh_2_, PPh_3_ and PMe_2_Ph, or PMe_3_ and PPh_3_.^44^

Of particular significance is the ^1^H–{^11^B} NMR spectrum of the binap-ligated reaction mixture, which exhibits numerous resonances in the negative region spanning from −0.35 to −11.38 ppm (see Figure 6). Remarkably, the low-frequency signals at −9.28 and −9.41 ppm exhibit slight overlap, presenting shapes akin to a *pseudo*-quintet and *pseudo*-quartet, respectively. These multiplets resolve into two doublets in the ^1^H–{^31^P} spectrum. However, upon closer inspection, beneath the doublet centered at −9.26 ppm lies a resonance that eludes precise resolution. This point is revealed in the ^1^H–{^31^P} spectrum at 223 K, which shows a broad *pseudo*-triplet at −9.17 ppm, to the left of the two doublets of different relative intensity (refer to Figure S16).

**Figure 6:**
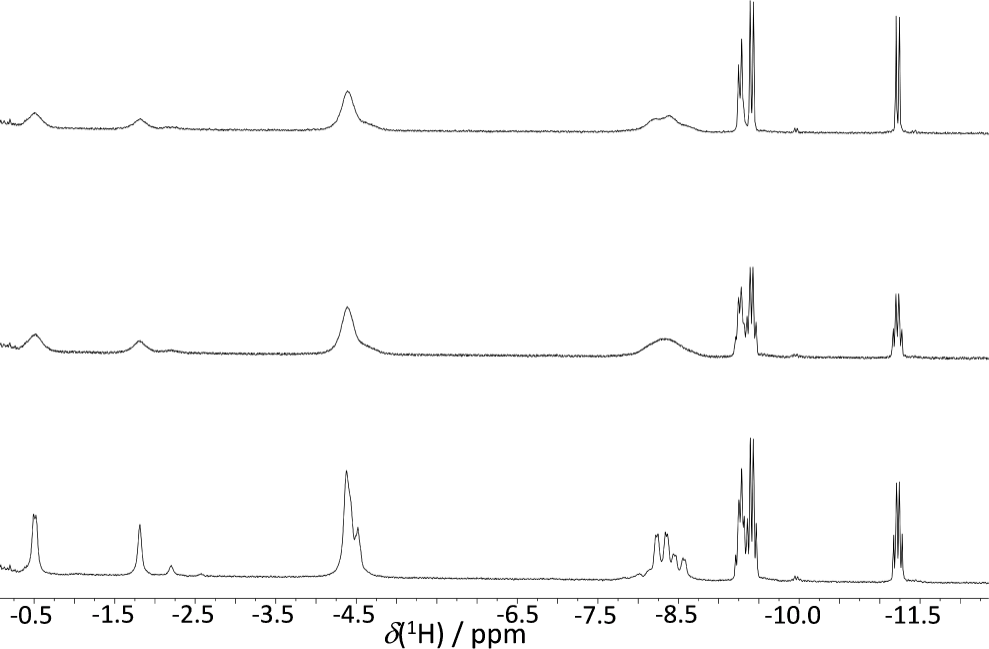
Room temperature ^1^H–{^11^B} (bottom), ^1^H–{^11^B­(off)} (middle), and ^1^H–{^31^P} (top) NMR spectra for the reaction mixture upon the treatment of **3** with TfOH under an atmosphere of H_2_(g).

Additionally, the ^1^H–{^11^B} spectrum displays an apparent quartet at −11.22 ppm that changes into a doublet under ^31^P decoupling. These low-frequency resonances retain their characteristic shapes in the conventional ^1^H NMR spectrum, confidently indicating their assignment to hydride ligands directly bound to rhodium centers in different chemical environments (see Figure 6).

Given the presence of at least four distinct Rh–H signals of varying relative intensities, these observations provide valuable insights into the structural complexity of the binap-ligated system. Additionally, at higher frequencies, the ^1^H–{^11^B} NMR spectrum displays two doublets of doublets of different intensity at −8.29 and −8.51 ppm, characterized by a coupling of approximately 60 Hz, indicative of strong ^2^ *J*_H,P_ coupling. These spectroscopic data resemble those of the dppbz-ligated system, for which the doublet of doublets resonance was assigned to an Rh–H–B hydrogen atom *trans* to a phosphorus group of the chelating dppbz ligand, forming the structure illustrated in figure as **6a**. In the case of the binap-system, there are two doublets of doublets of different intensity that should correspond to two diastereoisomers.

To clarify this point, we refer to the two isomeric *nido*-structures depicted in Scheme 2 as references for the formal formation of the minor and major isomers revealed by ^1^H NMR. The addition of a terminal hydrogen atom, *trans* to the sulfur vertex, and a bridging hydrogen atom along the Rh8–B9 edge, *trans* to one of the phosphorus centers, would afford the two isomers, which also bear a B–H–B hydrogen atom on the pentagonal open-face of the 11-vertex *nido*-clusters. The resonances of these bridging protons are assigned to the overlapping broad peaks at −4.37 and −4.42 ppm. Therefore, we propose the formation of binap-ligated isomers **8a** and **8b**, with the metal-to-thiaborane configuration depicted in Scheme 4.

It should be noted that the different intensities between the two rhodathiaboranes demonstrate that, in the reaction of hydrogen with the cationic system **5**, the formation of one diastereoisomer is more favorable than the other. Moreover, this diastereoselectivity could play a role in the enantiomeric excess of potential catalytic processes such as the hydrogenation of unsaturated organic molecules.

The third Rh–H hydride resonance, found around δ_H_ −9.33 ppm (see the ^1^H–{^31^P} spectrum at 223 K in Figure S16), exhibits a crossed peak correlation in the [^1^H–^1^H–{^11^B}]–COSY spectrum with the signal at δ_H_ −4.51 ppm (refer to Figure S17). This peak, which overlaps with the two B–H–B proton resonances assigned to isomers **8a** and **8b**, exhibits the pattern of a broad *pseudo*-triplet arising from the coupling with the ^103^Rh nucleus and the Rh–H hydride ligand. These considerations, along with the presence of a resonance at δ_H_ −1.80 ppm, lead us to propose a third hydridorhodathiaborane, **8c**, with an {RhH­(binap)}-to-{SB_9_H_8_(NC_5_H_5_)} configuration that resembles that of cluster **6b**, as illustrated in Scheme 3. Conformer **8c** should have its diasteroisomer counterpart, arising from the chiral nature of the binap ligand; however, there are no further signals in the ^1^H–{^11^B} NMR spectrum that could be assigned to the {RhH­(binap)}-to-{SB_9_H_8_(NC_5_H_5_)} configuration, diastereomeric of **8c** (Scheme 4).

**Scheme 4:**
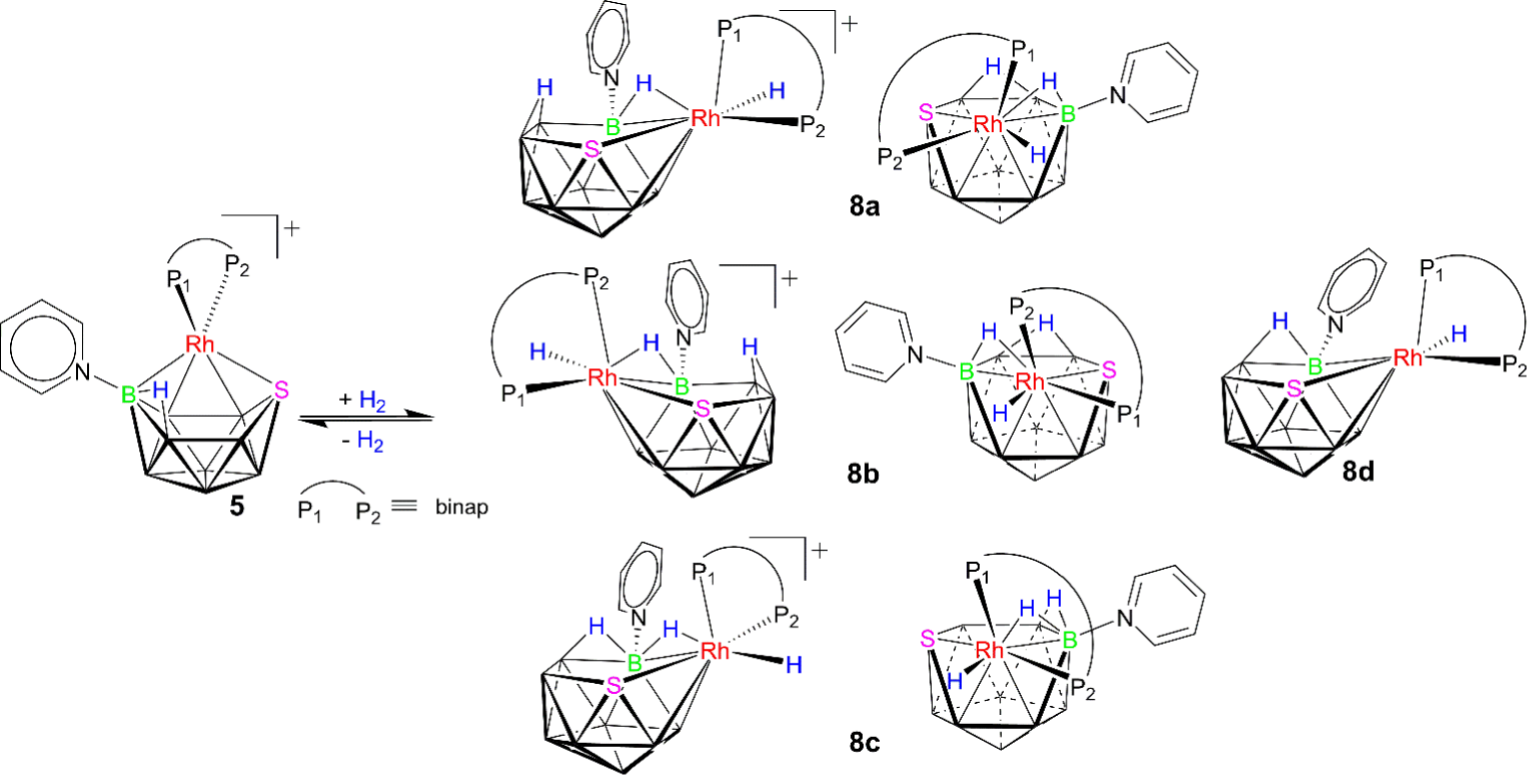
Reaction of Cationic [(binap)­(NC_5_H_5_)­RhSB_9_H_9_]^+^ (**5**) with Hydrogen to Give a Mixture of Hydridorhodathiaborane Conformers **8a**, **8b**, **8c**, and **8d**

In this context, the fourth hydride resonance found at the lowest-frequency (δ_H_ −11.22 ppm) could be related to the signal at −0.51 ppm, which, in the conventional proton spectrum, exhibits strong coupling to ^11^B nuclei. The chemical shifts of these resonances differ considerably from those of the other hydridorhodathiaboranes, described as isomers **8a**, **8b**, and **8c**, suggesting that this new species in the mixture features a different chemical formulation. Assuming that we can assign an Rh–H hydride ligand and a B–H–B bridging hydrogen along the pentagonal face of an 11-vertex *nido*-cluster, the fourth hydrorhodathiaborane could be proposed to be a neutral cluster with the formulation, [(binap)-*nido*-RhSB_9_H_9_(NC_5_H_5_)] (**8d**), which would be directly analogous to the parent compound **1**.

The [^1^H–^1^H]–NOESY spectrum of **5** under an atmosphere of H_2_ does not show off-diagonal peaks between the Rh–H–B and the Rh–H low-frequency resonances, nor between these signals and the peak corresponding to free hydrogen (see Figure S18). While this result does not conclusively rule out an H^+^/H^–^/H_2_ chemical exchange in the reaction mixture of **5** with hydrogen—such exchange has been demonstrated in the dppbz reaction system (refer to Figure S13)—it suggests that this process occurs more rapidly in the binap system. This observation is consistent with the chiral and flexible structure of binap, which leads to more dynamic metallathiaboranes.

Further evidence was obtained through deuterium labeling experiments, where the cationic dppbz- and binap-ligated clusters **4** and **5** were exposed to deuterium gas in an NMR tube. As expected, the ^1^H NMR spectra, in CD_2_Cl_2_, exhibited low-intensity residual signals in the negative region, corresponding to the B–H–B, B–H–Rh, and Rh–H proton resonances. In addition, it was possible to observe at δ­(^1^H) +4.57 ppm, the 1:1:1 relative intensity triplet, ^1^ *J*(^1^H–^2^D) = 43 Hz, that corresponds to the H–D molecule (Figures S19 and S21).

The ^2^D NMR spectrum for the reaction mixture of the cationic dpbbz-ligated rhodathiaborane **4** with D_2_(g), recorded in neat CH_2_Cl_2_, shows resonances of different relative intensities at δ­(^2^D) −3.4, −4.1, −5.8, −6.6, and −10.7 ppm, assigned to B–D–B, Rh–D–B, and Rh–D nuclei, respectively (Figure S20). These data confirm the formation of isomers **6a** and **6b**.

Under similar conditions, the cationic binap-ligated rhodathiaborane **5** exhibits resonances at δ­(^2^D) −4.5, −8.4, and −9.4 ppm (see Figure S22), indicating the formation of a single deuterated isomer. This contrasts with the mixture of isomers (**8a**, **8b**, **8c**, and **8d**) produced when reacting with H_2_. The difference in selectivity can be attributed to kinetic isotope effects (KIE), where the higher activation energies for breaking and forming D–D, B–D–B, Rh–D–B, and Rh–D bonds slow down the reaction compared to their hydrogen analogs, leading to distinct isomer equilibria.

The reduced structural flexibility of the dppbz derivatives, compared to that of the binap-ligated counterparts, results in slower reaction rates, allowing the formation of both isomers under D_2_ or H_2_ atmospheres. In contrast, the binap-ligated system, being more structurally labile, undergoes faster isomerization, shifting the equilibria toward a single conformer with D_2_, and producing different isomers under H_2_.

As described above, the ^1^H–{^11^B} spectrum of reaction mixture **5**, formed upon the treatment of the 11-vertex *closo*-rhodathiaborane **3** with triflic acid, presents multiple signals of small intensity within the δ­(^1^H) −1.5 to −11.5 ppm range. Comparing these data with those described for the reaction of mixture **5** with hydrogen (Figure 7) clearly reveals that simply treating the neutral binap-ligated cluster **3** with the strong acid results in the formation of hydridorhodathiaboranes **8a**, **8b**, **8c**, and **8d** with different relative intensity ratios. Interestingly, the spectrum of reaction mixture **5**, prior to hydrogen exposure, exhibits a fifth hydride resonance at δ­(^1^H) −9.96 ppm, a new broad doublet at −7.87 ppm, and an overlapping peak at −4.28 ppm.

**Figure 7:**
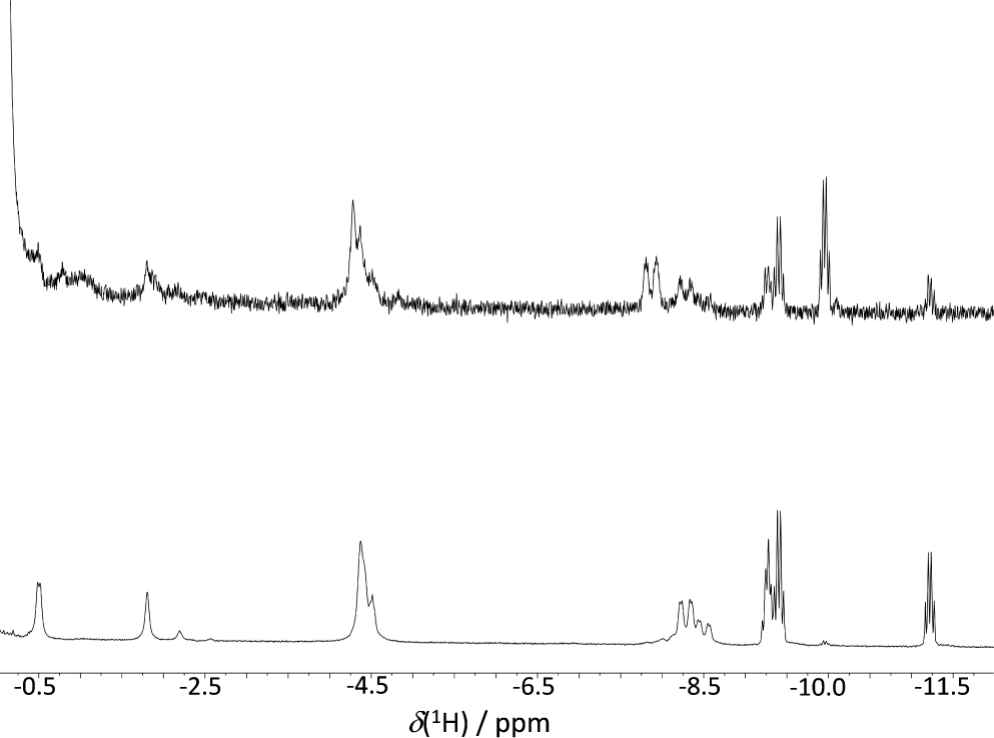
Room temperature ^1^H–{^11^B} spectra in CD_2_Cl_2_: **3** and TfOH (top); reaction mixture under an atmosphere of H_2_(g) (bottom).

These three resonances resemble the spectroscopic data assigned to the Rh–H, Rh–H–B, and B–H–B hydrogen atoms in isomers **8a** and **8b**, suggesting that in the reaction mixture of the protonated cluster **3**, another hydridorhodathiaborane with a different {RhH­(binap)}-to-{SB_9_H_8_(NC_5_H_5_)} configuration is formed.

It is important to note that these low-frequency resonances in the protonated sample (before hydrogen exposure, Figure 7) exhibit low intensity compared to B–H and binap hydrogen resonances found in the positive region of the spectrum. Additionally, as indicated above, the intensity of the resonances in the negative region of the ^1^H spectrum increases at low temperatures, indicating that the proposed hydridorhodathiaboranes are in equilibrium (see Figure S8).

The chemical behavior of cationic cluster **5** demonstrates the generation of H_2_ from cluster **3** upon protonation, a phenomenon not observed in the dppbz and dppe rhodathiaborane analogues. This finding emphasizes the tunability of the reactivity of polyhedral boron-based metal clusters through postsynthetic modifications, as highlighted in the Introduction section, using bidentate phosphines such as dppe (previous work, ref 35), dppbz, and binap.

The chiral binap ligand combines the steric effects of the twisted naphthyl group with a flexible electronic environment, resulting in interactions with the metal that differ significantly from those of the sterically rigid dppbz or the electronically less flexible dppe. These differences manifest in the distinct reactivity patterns observed in the corresponding 11-vertex rhodathiaboranes.

## Conclusions

This work presents the postsynthetic modification of the 11-vertex *nido*-rhodathiaborane **1** through reactions with the bidentate phosphines dppbz and binap, yielding the corresponding chelates, **2** and **3**. These compounds result from the substitution of PPh_3_ ligands at the metal center and concomitant hydrogen loss, leading to a *nido*-to-*closo* structural transformation. Protonation of the dppbz- and binap-ligated *closo*-rhodathiaboranes with triflic acid produces cationic species that are nonrigid in solution, undergoing intramolecular fluxional processes involving the metal-to-thiaborane linkage and proton tautomerism between different Rh–B and B–B edges. Moreover, the reaction of the binap-ligated system with a strong acid indicates the *in situ* evolution of hydrogen, which reacts rapidly with cationic intermediates to form a mixture of hydridorhodathiaboranes. The protons transform into hydride groups, increasing the structural and chemical lability of the metal–thiaborane ligand linkage and facilitating the coordination of H_2_ and its subsequent heterolytic cleavage on the cluster via cooperative metal–thiaborane ligand mechanisms. These results demonstrate that the polyhedral clusters act as electron reservoirs capable of converting protons and electrons into hydrogen. We believe this redox chemistry can open new and potentially useful reaction pathways.

## Experimental Section

### General Procedures

Reactions were carried out under an argon atmosphere using standard Schlenk-line techniques. Solvents were obtained dry from a Solvent Purification System of Innovative Technology Inc. The 11-vertex rhodathiaborane **1** was prepared according to the literature methods.^34^ NMR spectra were recorded on Bruker Avance 300-MHz, AV 400-MHz, and AV 500-MHz spectrometers, using ^13^C–APT, ^31^P–{^1^H}, ^11^B, ^11^B–{^1^H}, ^1^H, ^1^H–{^11^B}, and ^1^ H–{^11^B­(selective)} 1D techniques. In addition, COSY, NOESY, HSQC, and HMBC ^1^H–X (X = ^1^H, ^13^C, and ^31^P) 2D-correlation spectra were obtained using standard procedures.

Residual solvent protons were used as a reference (δ, ppm, CD_2_Cl_2_, +5.33). ^11^B chemical shifts are quoted relative to [BF_3_(OEt)_2_)] and ^31^P chemical shifts are quoted relative to 85% aqueous H_3_PO_4_. Infrared spectra were recorded on a PerkinElmer Spectrum-100 (ATR mode) FT-IR spectrometer. Carbon, hydrogen, and nitrogen analyses were performed using a PerkinElmer 240 B microanalyzer. High-resolution mass spectra were measured on a Micro Tof-Q Bruker Daltonics spectrometer.

X-ray diffraction data were collected on a Bruker D8 Venture diffractometer using graphite-monochromated Mo Kα radiation (λ = 0.71073 Å). Diffracted intensities were integrated and corrected for absorption effects using the multiscan method.^54–56^ Both programs are included in the APEX4 package. All the structures were solved by direct methods with SHELXS^57^ and refined by full-matrix least-squares on *F* ^2^ with SHELXL.^58^ Hydrogen atoms were located from difference Fourier maps and refined isotropically.

Single-crystals of **2** suitable for X-ray analysis were obtained from the reaction mixture, which was maintained at reflux temperature in dichloromethane for 10 days. Alternatively, the crystals of the binap derivative were grown in a 5 mm NMR tube in a fridge at 4 °C by slow diffusion of hexane into a 1,2-dichloroethane solution of compound **3**.

Structural data for [1,1-(η^2^-dppbz)-3-(NC_5_H_5_)-*closo*-1,2-RhSB_9_H_8_] (**2**): formula C_35_H_37_B_9_NP_2_RhS, MW = 765.85, orange block, orthorhombic *Pnma*, *a* = 19.1486(10) Å, *b* = 19.5599(11) Å, *c* = 9.6812(5) Å, *V* = 3626.0(3) Å^3^, *Z* = 4, *T* = 100(2) K, *D*_calcd_ = 1.403 g cm^–3^, μ = 0.645 mm^–1^, absorption correction factors min. 0.887, max. 0.950, 184073 reflections, 4656 unique (*R*_int_ = 0.039), 4547 observed, *R*_1_ = 0.0272 [*I* > 2σ­(*I*)], w*R*_2_(*F* ^2^) = 0.0591 (all data), GOF = 1.159. CCDC 2364150.

Structural data for [1,1-(η^2^-binap)-3-(NC_5_H_5_)-*closo*-1,2-RhSB_9_H_8_] (**3**): formula C_112_H_120_B_18_Cl_8_N_2_P_4_Rh_2_S_2_, MW = 2366.09, red prism, triclinic *P*1, *a* = 10.8340(5) Å, *b* = 11.3922(6) Å, *c* = 22.9877(12) Å, α = 79.647(2), β = 83.514(2), γ = 89.247(2)°, *V* = 2773.1(2) Å^3^, *Z* = 1, *T* = 100(2) K, *D*_calcd_ = 1.417 g cm^–3^, μ = 0.636 mm^–1^, absorption correction factors min. 0.881, max. 0.963, 149142 reflections, 27 508 unique (*R*_int_ = 0.042), 26990 observed, *R*_1_ = 0.0512 [*I* > 2σ­(*I*)], w*R*_2_(*F* ^2^) = 0.1377 (all data), GOF = 1.116. CCDC 2364149.

Structural data for [1,1-(η^2^-dpbbz)-3-(NC_5_H_5_)-4-(TfO)-*closo*-1,2-RhSB_9_H_8_] (**7**): formula C_37_H_38_B_9_Cl_2_F_3_NO_3_P_2_RhS_2_, MW = 998.84, yellow plate, monoclinic *P*21/*c*, *a* = 22.4040(12) Å, *b* = 9.8270(5) Å, *c* = 19.6606(10) Å, β = 97.317(2)°, *V* = 4293.3(4) Å^3^, *Z* = 4, *T* = 100(2) K, *D*_calcd_ = 1.545 g cm^–3^, μ = 0.746 mm^–1^, absorption correction factors min. 0.880, max. 0.978, 141741 reflections, 10 678 unique (*R*_int_ = 0.11), 8195 observed, *R*_1_ = 0.0577 [*I* > 2σ­(*I*)], w*R*_2_(*F* ^2^) = 0.1475 (all data), GOF = 1.047. CCDC 2364148.

### Synthesis of [1,1-(η^2^-dppbz)-3-(NC_5_H_5_)-*closo*-1,2-RhSB_9_H_8_] (**2**)

[8,8,8-(PPh_3_)_2_(H)-9-(NC_5_H_5_)-*nido*-8,7-RhSB_9_H_9_] (**1**) (28.9 mg, 0.0342 mmol), 1,2-bis­(diphenylphosphine)­benzene (dppbz), C_6_H_4_[P­(C_6_H_5_)_2_]_2_ (15.3 mg, 0.0342 mmol), and a magnetic follower were placed in a round-bottom flask with a Young connection. The flask was then evacuated and pressurized with argon, and then 5 mL of dry dichloromethane was added. The system was stirred at +60 °C for 10 days. In the reaction flask, the formation of red crystals and an orange-red solution was observed. The crystals were collected on a fritted disk to get 10.1 mg of pure compound **2** (0.0132 mmol, 39%). Elemental analysis: found: C 55.13%, H 4.94%, N 1.65%, S 3.61%. Calc. for C_35_B_9_H_37_NP_2_RhS (MW = 765.89): C 54.89%, H 4.87%, N 1.83%, S 4.19%. ^11^B NMR (96 MHz, CD_2_Cl_2_, 298 K, BF_3_(OEt)_2_): δ +54.4 (1 B, s, B3–py), +27.0 (1 B, d, ^1^ *J*_BH_ = 135 Hz, B9–H), +0.1 (2 B, br, B4,5–H), −12.3 (1 B, br, B8–H), −23.6 (1 B, ^1^ *J*_BH_ = 114 Hz, B6,7–H), −32.0 (2 B, d, ^1^ *J*_BH_ = 136 Hz, BH). ^1^H–{^11^B} NMR (500 MHz, CD_2_Cl_2_, 298 K, Me_4_Si): δ +7.83 (4 H, m, *H*_o_-C_6_H_5_), +7.65 (1 H, t, 8.7 Hz, *H*_*p*_-NC_5_H_5_), +7.57 (2 H, d, 5.9 Hz, *H*_*m*_-NC_5_H_5_), +7.43 (6 H, m, C_6_H_5_, C_6_H_4_), +7.37 (4 H, m, *H*_*m*_-C_6_H_5_), +7.31 (2 H, *pseudo*-t,), +7.20 (4 H, *pseudo*-t, 7.6 Hz, *H*_*p*_-C_6_H_5_), +7.02 (4 H, m,), +6.81 (2 H, overlapping d’s, 7 Hz, *H*_*m*_-C_6_H_4_), +4.03 (1 H, br s, *H*_*t*_–B9), +2.52 (1 H, br s, *H*_*t*_–B8), +1.88 (2 H, br s, *H*_*t*_–B4,5), +0.12 (2 H, br s, *H*_*t*_–B6,7), −0.14 (2 H, br s, *H*_*t*_–B10,11). ^31^P–{^1^H} NMR (202 MHz, CD_2_Cl_2_, 298 K, H_3_PO_4_): δ +68.4 (2 P, d, ^1^ *J*_PH_ = 153 Hz, Ph_2_ *P*-C_6_H_4_-*P*Ph_2_). HRMS (μ-TOF): C_35_H_36_B_9_NP_2_SRhNa [M – H + Na]^+^: calcd, 789.1967, found, 789.1934.

### Synthesis of [1,1-(η^2^-binap)-3-(NC_5_H_5_)-*closo*-1,2-RhSB_9_H_8_] (**3**)

Compound **1** (98.5 mg, 0.1643 mmol), (*R*)-(+)-2,2′-bis­(diphenylphosphino)-1,1′-binaphthyl (binap), [(C_6_H_5_)_2_PC_10_H_6_]_2_ (72.5 mg, 0.1643 mmol), and a magnetic follower were placed in a round-bottom flask with a Young connection. The flask was then evacuated and pressurized with argon, and then 8 mL of dry dichloromethane was added. The system was stirred at +60 °C for 10 days. In the reaction flask, the formation of red crystals and an orange-red solution was observed. The initial orange-red solution turned to deep-red. The solution was transferred to a Schlenk tube, the solvent reduced in volume, and the concentrated solution was over layered with hexane, to be finally kept in the fridge at +4 °C for 2 days. After this procedure, the formation of red crystalline needles was observed, which were isolated and dried under vacuum to give 48.7 mg of pure compound **3** (5.76 × 10^–5^ mmol, 35%). Elemental analysis: found: C 59.49%, H 4.81%, N 1.50%, S 3.23. Calc. for C_49_H_45_B_9_NP_2_RhSNa (MW= 966.2533): C, 56.85; H, 5.11; N, 1.18; S, 2.71. ^11^B-{^1^H} NMR (96 MHz, CD_2_Cl_2_, 298 K, BF_3_(OEt)_2_): δ +54.0 (1 B, s, B3-py), +25.9 (1 B, d,^1^ *J*_BH_ = 131 Hz, B9), −0.2 (1 B, br, B4), −1.3 (1 B, br, B5), −15.5 (1 B, br, B8), −22.6 (1 BH, br d, ^1^ *J*_BH_ = 92 Hz, B6), −27.5 (1 BH, d, ^1^ *J*_BH_ = 146 Hz, B6), −29.7 (1 BH, d, ^1^ *J*_BH_= 133 Hz, B10), −30.7 (2 BH, d, ^1^ *J*_BH_ = 126 Hz, B11). ^1^H–{^11^B} NMR (400 MHz, CD_2_Cl_2_, 298 K, Me_4_Si): δ +9.13 (2 H, d, ^3^ *J*_H,H_ = 5.6 Hz; *ortho-H*-NC_5_H_5_), +8.29 (1 H, t, ^3^ *J*_H,H_ = 7.8 Hz; *para-H*-NC_5_H_5_), +8.19 (1 H, *pseudo*-t, *J* = 8.7 Hz; becomes a d upon ^31^P decoupling, *J* = 8.8 Hz, *ortho-H*-binap), +7.89 (1 H, apparent d, *J* = 8.5 Hz, binap), +7.72 (2 H, *pseudo*-t, *J* = 7.4 Hz, *para-H*-binap), +7.65 (2 H, m, *ortho-H*-binap), +7.62 (2 H, m that becomes a d upon ^31^P decoupling, *J* = 9.0 Hz, *ortho-H*-binap), +7.41 (1 H, *pseudo*-d, *J* = 7.40 Hz, binap), +7.32 to +7.18 (10 H, m, binap), +7.09 (2 H, *pseudo*-t, becomes a d upon ^31^P decopling, *J* = 9.3 Hz, *ortho-H*-binap), +7.03 (2 H, br *pseudo*-t, becomes a d upon ^31^P decopling, *J* = 7.3 Hz, binap), +6.87 (1 H, *pseudo*-t, *J* = 7.7 Hz, *para-H*-binap), +6.72 (1 H, *pseudo*-t, *J* = 7.5 Hz, *para-H* binap), +6.52 (4 H, m, binap), +6.45 (1 H, *pseudo*-t, *J* = 7.7 Hz, binap), +6.25 (2 H, *pseudo*-t, *J* = 7.1 Hz, *ortho-H*-binap), +6.10 (1 H, d, *J* = 9.3 Hz, binap), +3.97 (s, 1H, *H*_*t*_–B9), +2.21 (s, 1H, *H*_*t*_–B8), +2.09 (s, 1H, *H*_*t*_–B4), +1.07 (s, 1H, *H*_*t*_–B5), −0.86 (s, 1H, *H*_*t*_–B6), +0.15 (s, 1H, *H*_*t*_–B7), −0.11 (s, 1H, *H*_*t*_–B10), −0.24 (s, 1H, *H*_*t*_–B11). ATP–^13^C: δ +148.1 (*ortho-C*-NC_5_H_5_), +141.5 (*para-C*-NC_5_H_5_), +128 (*ortho-C-Ph*-binap), +126.4 (*metha-C*-NC_5_H_5_), +135.1 (*ortho-C-naphthyl*-binap), +135.5, +135.1, +134.7, from +130.0 to +126.4 (multiple signals). ^31^P–{^1^H} NMR (121.5 MHz, CD_2_Cl_2_, 298 K, H_3_PO_4_): second-order spectrum that exhibits the formation of a pseudotriplet of doublets, centered at δ +34.4; analysis of the resonance, using the computer program gNMR, yielded the following chemical shifts and coupling constants: δ +34.9 (^2^ *J*_PP_ = +33 Hz, ^1^ *J*_RhP_ = 144 Hz), +33.9 (^2^ *J*_PP_ = +33 Hz, ^1^ *J*_RhP_ = 150 Hz). HRMS (μ-TOF): C_49_H_45_B_9_NP_2_SRhNa [M + Na]^+^: calcd, 966.2678, found, 966.2553.

### [1,1-(η^2^-dppbz)-3-(NC_5_H_5_)-*closo*-1,2-RhSB_9_H_9_]­[TfO] (**4**) and [1,1-(η^2^-binap)-3-(NC_5_H_5_)-*closo*-1,2-RhSB_9_H_9_]­[TfO] (**5**)

Two quick-pressure valve NMR tubes were loaded with each of the 11-vertex rhodathiaboranes, **2** and **3**, using 7.9 mg (0.0103 mmol) and 10.0 mg (0.0106 mmol), respectively. Then, we added 0.4 mL of CD_2_Cl_2_ under an atmosphere of argon. The compounds were partially dissolved to give saturated orange-red (compound **2**) and bright red (compound **3**) solutions, in equilibrium with the solids. To each NMR tube, we added stoichiometric amounts of triflic acid: 0.9 μL (0.0103 mmol) to compound **2** and 1.0 μL (0.0106 mmol) to **3**. Upon the addition of the acid, the solids dissolved completely to afford a pale-yellow solution, in the case of the dppbz derivative **2**, and a red solution in the case of the reaction with **3**.

Both reactions were studied by NMR spectroscopy using a combination of 1D and 2D experiments as indicated above, in the General Procedures section. We report selected 1D NMR data for the cationic rhodathiaboranes.

### Compound 4

IR (solid, cm^–1^): 3201 (br s) ν­(OH); 2490 ν­(br s, BH); 2159 ν­(Rh–H), 2033 ν­(Rh–H),) 1432 δ­(s, C–H), 1191, 1027 δ­(s, C–H). HRMS (μ-TOF): C_35_H_38_B_9_NP_2_SRh [M]^+^: calc. 768.2205, [M – H]^+^, found, 767.2203. ^11^B NMR (162 MHz, CD_2_Cl_2_, 298 K, BF_3_(OEt)_2_): δ +55.8 (1B, s, B3–NC_5_H_5_), +30.5 (1B, d, ^1^ *J*_BH_ = 112 Hz, B9–H), +11.6 (2B, br, B4,5–H), −11.3 (1B, br, B8–H), −20.0 (2B, d, ^1^ *J*_BH_ = 117 Hz, B6,7–H), −22.4 (2B, ^1^ *J*_BH_ = 140 Hz, B10,11–H). ^1^H–{^11^B} NMR (500 MHz, CD_2_Cl_2_, 298 K, Me_4_Si): δ +9.71 (br s, excess of TfOH), +8.36 (1H, *pseudo*-t, ^3^ *J*_HH_ = 7.6 Hz, *para*-*H*-NC_5_H_5_), +8.17 (2H, d, ^3^ *J*_HH_ = 5.3 Hz, *ortho-H*-NC_5_H_5_), +7.68 (4 H, m, C_6_ *H*_5_, C_6_ *H*_4_), +7.61 (4 H, m, C_6_ *H*_5_, C_6_ *H*_4_), +7.54 (6 H, m, C_6_ *H*_5_, C_6_ *H*_4_), +7.46 (4 H, m, C_6_ *H*_5_, C_6_ *H*_4_), +7.41 (4 H, m, C_6_ *H*_5_, C_6_ *H*_4_), +7.26 (4 H, m, *ortho-H*-C_6_ *H*_5_), +4.66 (1H, br s, *H*_*t*_–B9), +2.79 (2H, br s, *H*_*t*_–B4,5), +2.35 (1 H, br s, *H*_*t*_–B8), +0.85 (2 H, br s, *H*_*t*_–B6,7), +0.62 (2 H, br s, *H*_*t*_–B10,11), −6.32 (1H, br dt, 14 Hz, B3–H–Rh1; in the ^1^H–{^31^P} spectrum becomes a br. doublet, ^2^ *J*_PH_ = 22 Hz). ^13^C–{^1^H}-ATP: δ +145.6 (CH, *ortho*-*C*-NC_6_H_5_), +144.6 (CH, *para*-*C*-NC_6_H_5_), +133.4 (CH, *meta*-*C*-NC_6_H_5_), +132.5 (CH, *ortho*-*C*-C_6_H_5_), +132.4 (CH, *ortho*-*C*-C_6_H_5_), +132.2 (CH, *ortho*-*C*-C_6_H_5_), +129.5 (CH, *meta*-*C*-C_6_H_5_), +129.4 (CH, *meta*-*C*-C_6_H_5_), +127.3 (CH, *meta*-C_6_H_4_). ^31^P–{^1^H} NMR (162 MHz, CD_2_Cl_2_, 298 K, H_3_PO_4_): δ +56.6 (2P, d, ^2^ *J*_RhP_ = 105 Hz). [^1^H–^1^H]-NOESY (500 MHz, CD_2_Cl_2_, 300 K): (δ_f2_, δ_f1_) (+8.36, +7.68), (+7.68, +8.17), (−6.32, +8.17), (−6.32, +7.26).

There are signals of very small intensity in the ^1^H NMR spectrum at: δ −3.31 (B-*H*-B) and −14.99 (multiplet that becomes a doublet upon ^31^P decoupling, ^1^ *J*_RhH_ = 17 Hz, Rh–*H*).

### Compound 5

IR (solid, cm^–1^): 3200 ν­(br, OH); 2521 ν­(br s, BH); 2158 ν­(Rh–H), 1976 ν­(Rh–H),) 1435 δ­(s, C–H), 1192,1027 δ­(s, C–H). HRMS (μ-TOF): C_49_H_46_B_9_NP_2_RhS [M]^+^ calc., 944.2859, [M]^+^, found, 944.2736. ^11^B NMR (96 MHz, CD_2_Cl_2_, 298 K, BF_3_(OEt)_2_): δ +25.9 (1B, br. s, B9–NC_5_H_5_), +16.4 (1B, br., B3–H), +8.4 (1B, br. s, B11–H), +3.8 (2B, ^1^ *J*_B,H_ = 127 Hz, B6–H), −2.7 (1B, br s, B4–H), −5.6 (1B, br. s, B5–H), −18.6 (1B, br d, ^1^ *J*_B,H_ = 137 Hz, B1H), −20.9 (1B, br, B10H), −23.1 (1B, br, B2H). ^1^H–{^11^B} NMR (500 MHz, CD_2_Cl_2_, 298 K, Me_4_Si): δ +8.51 (2 H, d, ^3^ *J*_H,H_ = 5.81 Hz, *ortho-H-*NC_5_H_5_), +8.36 (1 H, t, *J* = 9.18; in the ^1^H–-{^31^P} spectrum becomes a doublet, *J* = 9.1 Hz, *ortho-H-*C_6_H_5_P-binap), +8.23 (1 H, 1 H, t, ^3^ *J*_H,H_ = 9.18, *para-H-*NC_5_H_5_), +7.97 (1 H, d, *J* = 9.3 Hz), +7.90 (1 H, t, *J* = 9.3 Hz; in the ^1^H–{^31^P} spectrum becomes a doublet, *J* = 9.1 Hz, *ortho-H-*C_6_H_5_P-binap), +7.81 to +7.54 (14 H. m, binap), +7.33 (1 H, t, *J* = 8.2 Hz), +7.28 (1 H, t, *J* = 6.9 Hz), +6.91 (1 H, t, *J* = 8.2 Hz), +6.81 (3 H, m; in the ^1^H–{^31^P} spectrum becomes a doublet, *J* = 7.2 Hz, *ortho-H-*binap), +6.72 (3 H, m), +6.56 (1 H, m), +6.48 (2 H, m), +6.42 (2 H, m), +6.25 (1 H, d, *J* = 9.0 Hz), +5.89 (1 H, d, *J* = 9.0 Hz), +4.11 (br s, 1H; BH), +3.35 (br s, 1H; BH)), +2.80 (br s, 1H; BH), +2.53 (br s, 1H; BH), +2.01 (br s, 1H; BH), +1.74 (br s, 1H; BH), +1.60 (br s, 1H; BH), +1.50 (br s, 1H; BH), +0.64 (br s, 1H; BH), −0.08 (br s, 1H; BH), −4.28 (low intensity s, B–H–B), −7.86 (low intensity d, *J* = 66.9 Hz, Rh–H–B), −8.28 (low intensity d, *J* = 64.4 Hz, Rh–H–B), −9.27 (low intensity m, Rh–H; d upon ^31^P decoupling ^1^ *J*_RhH_ = 20 Hz), −9.41 (low intensity *pseudo*-q, ^1^ *J*_RhH_ = 18.6 Hz, Rh–H; d upon ^31^P decoupling ^1^ *J*_RhH_ = 21 Hz), −9.96 (low intensity *pseudo*-q, ^1^ *J*_RhH_ = 18.6 Hz, Rh–H; d upon ^31^P decoupling ^1^ *J*_RhH_ = 19 Hz), −11.22 (low intensity *pseudo*-q, ^1^ *J*_RhH_ = 18.0 Hz, Rh–H; d upon ^31^P decoupling ^1^ *J*_RhH_ = 20 Hz). ^31^P–{^1^H} NMR (202 MHz, CD_2_Cl_2_, 298 K, H_3_PO_4_): δ +35.6 (dd, ^1^ *J*_Rh,P_ = 142 Hz, ^2^ *J*_P,P_ = 41 Hz), +15.7 (br dd, ^1^ *J*_Rh,P_ = 121 Hz, ^2^ *J*_P,P_ = 41 Hz). ^31^P–{^1^H} NMR (202 MHz, CD_2_Cl_2_, 183 K, H_3_PO_4_): δ +45.8 (0.02 P, br d, small intensity, ^1^ *J*_Rh,P_ = 111 Hz), +38.4 (0.12 P, br dd, small intensity, ^1^ *J*_Rh,P_ = 108 Hz), +36.5 (1 P, dd, ^1^ *J*_Rh,P_ = 151 Hz, ^2^ *J*_P,P_ = 46 Hz), +31.8 (0.25 P. br d, ^1^ *J*_Rh,P_ = 103 Hz), +31.5 (0.12 P. br d, overlaid with the previous peak), +26.7 (0.01 P, br d, ^1^ *J*_Rh,P_ = 110 Hz), +20.5 (0.25 P. br d, ^1^ *J*_Rh,P_ = 103 Hz), +15.1 (1 P, dd, ^1^ *J*_RhP_ = 122.9, ^2^ *J*_PP_ = 46 Hz).

Alternatively, compounds **4** and **5** were prepared in Schlenk tubes under an atmosphere of argon. Initially, 10 mg (0.0131 mmol) of the starting dppbz- and binap-ligated compounds, **2** and **3**, were placed into separate Schlenk tubes, which were evacuated and filled with argon three times. Each rhodathiaborane was then dissolved in 3 mL of deoxygenated dichloromethane. Under a flow of argon, the corresponding stoichiometric amounts of triflic acid (1.18 and 0.94 μL) were introduced into each tube. The reaction occurred instantaneously. The solvent was then evaporated to dryness under a vacuum, yielding a pale yellow solid. The solid was then isolated and weighed under air, resulting in final yields of 7.6 mg (62.9%) for compound **4** and 9.3 mg (80%) for compound **5**.

### [1,1,1-(H)­(η^2^-dppbz)-3-(NC_5_H_5_)-1,2-RhSB_9_H_10_]­[TfO] (6) and [1,1,1-(H)­(η^2^-binap)-3-(NC_5_H_5_)-1,2-RhSB_9_H_10_]­[TfO] (8)

The two quick-pressure valve NMR tubes above, which contained cationic rhodathiaboranes **4** and **5**, after treatment with triflic acid, were exposed to an atmosphere of dihydrogen of 10 bar.

Both reactions were studied by MNR spectroscopy using a combination of 1D and 2D (see the Supporting Information). For each reaction, the spectroscopic data demonstrated the formation of mixtures of hydridorhodathiaborane isomers, named systems **6** and **8**. The NMR data of these species are gathered.

### System 6

^11^B NMR (162 MHz, CD_2_Cl_2_, 298 K, BF_3_(OEt)_2_): δ +16.6 (1B, d, ^1^ *J*_BH_ = 110 Hz), +10.6 (1B), +5.2 (2B, br, B4,5–H), +3.3 (1B, br, B8–H), −8.8 (2B, d, ^1^ *J*_BH_ = 117 Hz, B6,7–H), −11.9 (1B, ^1^ *J*_BH_ = 111 Hz), −16.1 (1B, ^1^ *J*_BH_ = 140 Hz), −19.4 (1B, br), −22.7 (1B, ^1^ *J*_BH_ = 144 Hz), −24.6 (1B, ^1^ *J*_BH_ = 150 Hz). Some of these signals overlap with the resonances of compound **4**. ^1^H–{^11^B} NMR (500 MHz, CD_2_Cl_2_, 298 K, Me_4_Si): δ +9.71 (br s, excess of TfOH), +8.61 (2H, ^3^ *J*_HH_ = 5.5 Hz, *ortho*-*H*-NC_5_H_5_), +8.36 (0.55 H, t, ^3^ *J*_HH_ = 7.6 Hz, *para-H*-NC_5_H_5_, compound **4**), +8.24 (1 H, t, ^3^ *J*_HH_ = 8.1 Hz, *para-H*-NC_5_H_5_), +8.18 (1 H, d, ^3^ *J*_HH_ = 6.6 Hz, *ortho*-*H*-NC_5_H_5_), +8.04 (2H, m, ^3^ *J*_HH_ = 8.0 Hz, *para-H*-NC_5_H_5_), +7.93 (2 H, m, ^3^ *J*_HH_ = 8.0 Hz, *ortho-H*-C_6_H_5_), +7.79 (br s, *ortho-H*-C_6_H_5_), (+7.64, m, C_6_ *H*_5_, C_6_ *H*_4_), (+7.54, m, *metha-H*-NC_5_H_5_), +7.50 to +6.76 (overlapping multiplets, C_6_ *H*_5_, C_6_ *H*_4_), +4.61­(free H_2_), +3.78 (BH), +3.70 (BH), +1.62 (BH), +1.50 (BH), −3.33 (B–*H*–B), −3.95 (B–*H*–B), −5.70 (Rh–*H*–B), −6.50 (dd, ^2^ *J*_PH_ = 57.9 Hz, ^1^ *J*_RhH_ = 16.9 Hz, Rh–*H*–B), −10.57 (p, *J* = 14.0 Hz, 1H; in the ^1^H–{^31^P} spectrum becomes a br. *pseudo*-triplet, *J* ≈ 15 Hz), −10.75 (dd, *J* = 22.8 Hz, *J* = 13.5 Hz, 1H; in the ^1^H–{^31^P} spectrum becomes a doublet, ^2^ *J*_PH_ = 23.1 Hz), −15.03 (m of small intensity, *J* = 14.5 Hz, *J* ≈ 12.3 Hz; in the ^1^H–{^31^P} spectrum becomes a br. doublet, ^2^ *J*_PH_ = 17 Hz). ^31^P–{^1^H} NMR (162 MHz, CD_2_Cl_2_, 298 K, H_3_PO_4_): δ +66.2 (1P, dd, ^1^ *J*_RhP_ = 119 Hz, ^1^ *J*_PP_ = 20 Hz), +65.6 (1P, d, ^1^ *J*_RhP_ = 126 Hz), +59.2 (1P, dd, ^1^ *J*_RhP_ = 98 Hz, ^1^ *J*_PP_ = 20 Hz), +56.6 (1P, d, ^1^ *J*_RhP_ = 105 Hz), +51.8 (d, ^1^ *J*_RhP_ = 95 Hz). [^1^H–^31^P]-HMBC (500 MHz, CD_2_Cl_2_, 300 K): (δ_f2_, δ_f1_) (+59.2, −10.75), (+66.2, +7.93), (+66.2, +7.64), (+66.2, +7.53), (+66.2, +7.17). [^1^H–^1^H]-NOESY (500 MHz, CD_2_Cl_2_, 300 K): (δ_f2_, δ_f1_) (+8.61, −10.75), (+8.04, −10.75), (+8.04, −10.75), (+7.93, −10.57), (+7.79, −10.75), (+7.07, −10.57), (+7.07, −10.75); the following cross peaks are due to chemical exchange: (+4.61, −5.70), (+4.61, −6.50), (+4.61, −10.57), (+4.61, −10.75), (−3.33, −3.95), (−10.57, −3.33), (−10.75, −3.95), (−10.75, −3.33), (−10.75, −3.95).

### System 8

^11^B–{^1^H} NMR (160 MHz, CD_2_Cl_2_, 298 K, BF_3_(OEt)_2_): δ +14.0 (1 B, br, B–H), +12.7 (1 B, br, B–H), +5.5 (2B, br, B–H), +3.2 (1 B, br, B–H), −4.5 (1 B, br, B–H), −8.8 (1 B, B–H), −10.0 (1 B, d, ^1^ *J*_BH_ = 159 Hz, B–H), −11.3 (1 B, br), −14.7 (1 B,), −16.7 (1 B), −16.7 (1 B), −20.6 (1 B), −22.5 (1 B), −23.3 (1 B). −25.4 (1 B). −28.4 (1 B d, ^1^ *J*_BH_ = 156 Hz, B–H). ^1^H–{^11^B} NMR (500 MHz, CD_2_Cl_2_, 298 K, Me_4_Si): δ +8.80 (br, small intensity), +8.68 (br, small intensity), +8.39 (1 H, t, ^3^ *J*_HH_ = 8.2 Hz, *para-H*-NC_5_H_5_), +8.32 (1 H, t, ^3^ *J*_HH_ = 7.4 Hz, *para-H*-NC_5_H_5_), +8.28 (1 H, d, ^3^ *J*_HH_ = 5.0 Hz, *ortho*-*H*-NC_5_H_5_), +8.20 (1H, br), +8.09 (2 H, m), +7.98 (m), +7.89 (br d, 8.2 Hz), +7.81 (overlaying d, 9.2 Hz), +7.75 to +7.17 (overlaying multiplets, binap), +7.11 (t, ^3^ *J*_HH_ = 9.1 Hz), +6.91 (m), +6.80 (m), +6.73 (br s), +6.66 to +6.52 (overlaying signals), +6.44 (br t, 7.5 Hz), +6.38 (d, 9.3 Hz), +6.32 (d, 8.6 Hz), +6.29 (d, 8.6 Hz), +6.10 (d, 10.0 Hz), +6.07 (d, 8.6 Hz), +6.32 (d, 8.6 Hz), +4.61­(excess of free H_2_), +4.15 (BH), +3.89 (BH), +3.75 (BH), +3.28 (BH), +3.21 (BH), +3.11 (BH), +2.65 (BH), +2.49 (BH), +2.36 (BH), +2.07 (BH), +1.97 (BH), +1.72 (BH), +1.15 (BH), −0.5 (br d, 16.8 Hz, BH), −1.81 (B–*H*–B), −2.20 (small intensity peak, B–H–B), −4.38 (B–H–B), −4.43 (B–H–B), −4.52 (br t, 14 Hz, B–H–B), −8.29 (dd, ^2^ *J*_PH_ = 60.7 Hz, ^1^ *J*_RhH_ = 11.5 Hz, Rh–*H*–B), −8.51 (dd, ^2^ *J*_PH_ = 57.0 Hz, ^1^ *J*_RhH_ = 13.3 Hz, Rh–*H*–B), −9.26 (*pseudo*-q, *J* = 20.1 Hz, 1H; in the ^1^H–{^31^P} spectrum becomes a d, *J* ≈ 19.4 Hz), −9.41 (*pseudo*-q, *J* = 18.2 Hz, 1H; in the ^1^H–{^31^P} spectrum becomes a d, ^1^ *J*_RhH_ = 20.6 Hz), −9.96 (low intensity *pseudo*-q, *J* = 17.7 Hz, 1H; in the ^1^H–{^31^P} spectrum becomes a d, ^1^ *J*_RhH_ = 16.6 Hz), −11.23 (*pseudo*-q, *J* = 17.7 Hz, 1H; in the ^1^H–{^31^P} spectrum becomes a d, ^1^ *J*_RhH_ = 20.4 Hz. ^31^P–{^1^H} NMR (202 MHz, CD_2_Cl_2_, 298 K, H_3_PO_4_): δ +38.58 (overlaying second-order signals, *J* = 124.0, 81.8, 45.8 Hz), +32.67 (second-order *pseudo*-td, *J* = 100.2, 36.1 Hz), +31.54 (dd, *J* = 100.8, 30.5 Hz), ca. +31.00 (overlaid with the previous peak, br d). [^1^H–^31^P]-HMBC (500 MHz, CD_2_Cl_2_, 300 K): (δ_f2_, δ_f1_) (+59.2, −10.75), (+66.2, +7.93), (+66.2, +7.64), (+66.2, +7.53), (+66.2, +7.17). [^1^H–^1^H]-NOESY (500 MHz, CD_2_Cl_2_, 300 K): (δ_f2_, δ_f1_) (+8.61, −10.75), (+8.04, −10.75), (+8.04, −10.75), (+7.93, −10.57), (+7.93, −10.75), (+7.79, −10.57), (+7.79, −10.75), (+7.07, −10.57), (+7.07, −10.75); the following cross peaks are due to chemical exchange: (+4.61, −5.70), (+4.61, −6.50), (+4.61, −10.57), (+4.61, −10.75), (−3.33, −3.95), (−10.57, −3.33), (−10.75, −3.95), (−10.75, −3.33), (−10.75, −3.95).

## Supplementary Material



## Notes

1D and 2D NMR spectra for all compounds; IR spectra for cationic **4** and **5** (PDF)
