# Supplementary material for: Protonation of Dppbz- and Binap-Ligated Rhodathiaboranes Yielding Hydron, Hydride, and Hydrogen Exchange
Source: Inorg Chem. 2024 Oct 8;63(42):19593–606. doi: 10.1021/acs.inorgchem.4c02652 (PMC12616688; doi:10.1021/acs.inorgchem.4c02652)
Supplement: Supplementary file 1 [file ic4c02652_si_001.pdf]

Protonation of dppbz- and binap-ligated rhodathiaboranes yielding hydron, hydride and hydrogen exchange

*Javier Vidondo, Laura Urdániz, Pablo J. Sanz Miguel, Ricardo Rodríguez and Ramón Macías**

Departamento de Química Inorgánica, Instituto de Síntesis Química y Catálisis Homogénea (ISQCH), Universidad de Zaragoza-CSIC, 50009 Zaragoza, Spain;

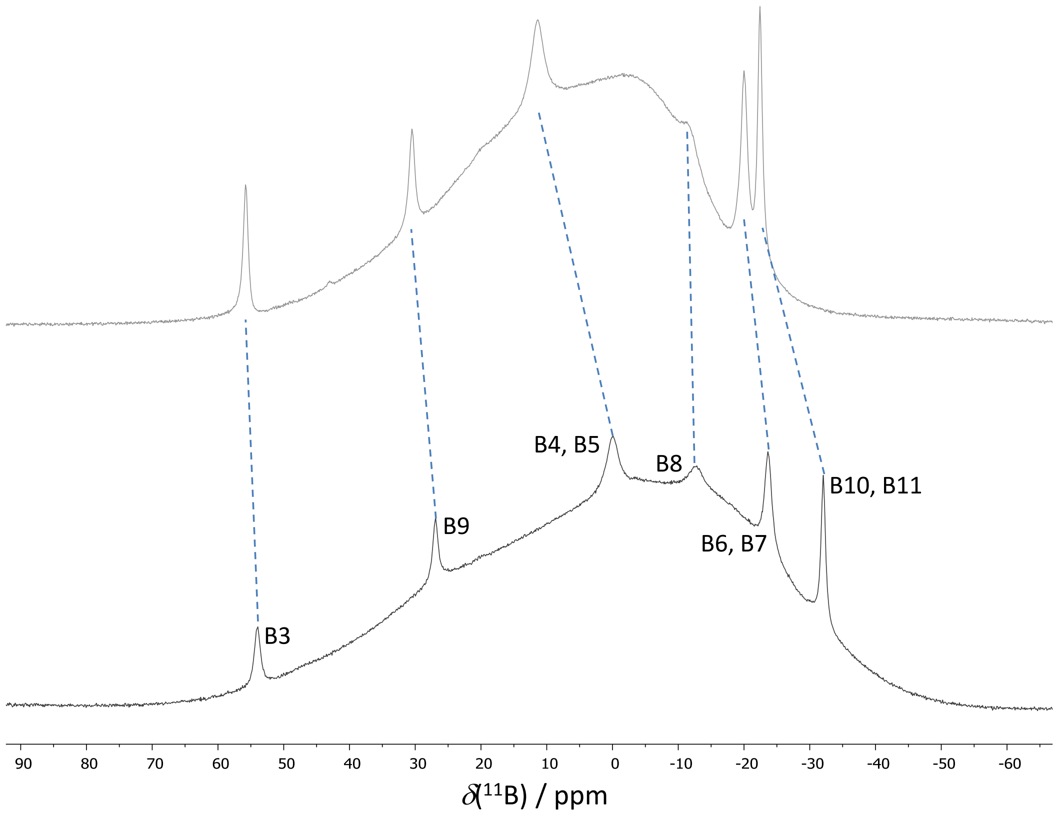

Figure S1 Room temperature $^{11}B–\{^{1}H\}$ NMR spectra for compound **2** (bottom) and for its protonated derivative **4** (top).

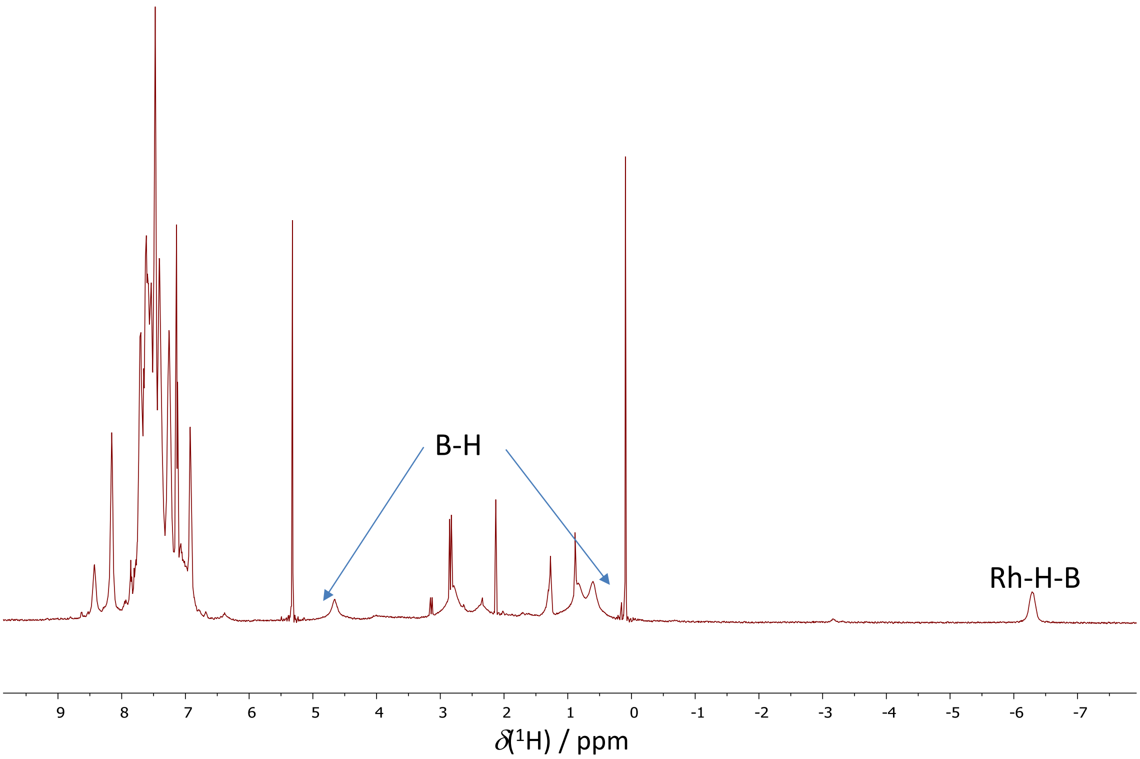

Figure S2 Room temperature ^{1}H–{^{11}B(BB)} NMR spectrum for compound **4**.

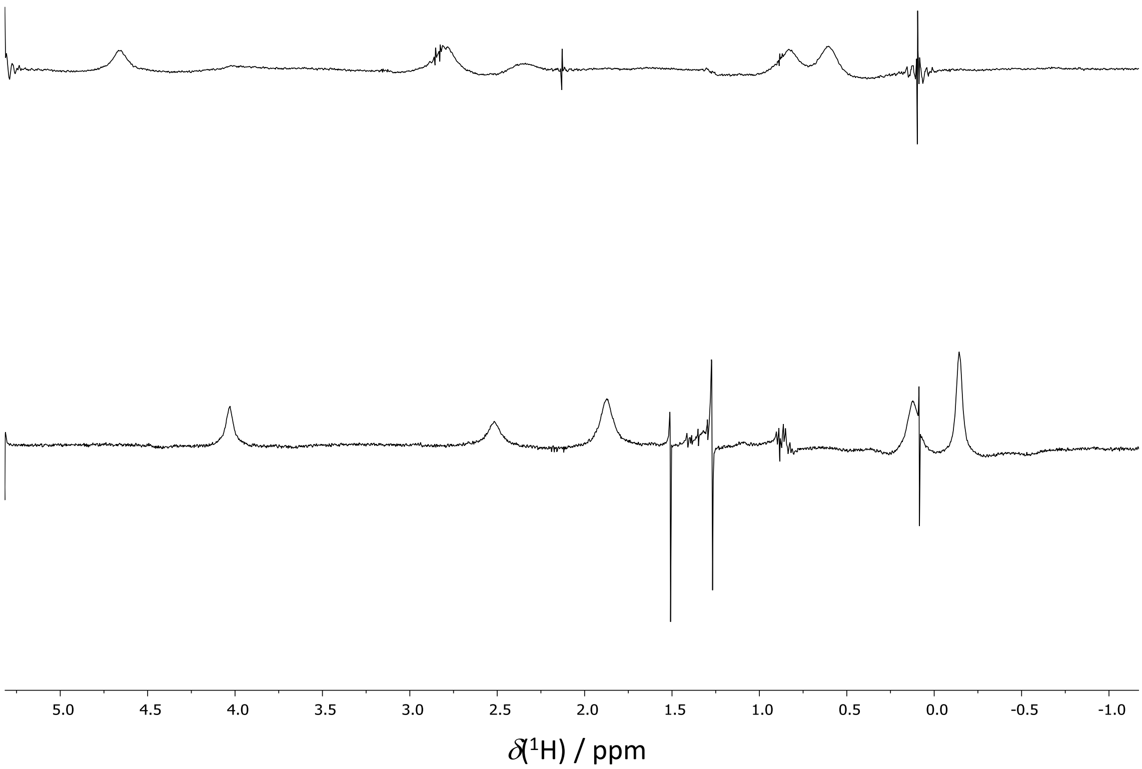

Figure S3 Room temperature ^{1}H–{^{11}B(BB)} – ^{1}H–{^{11}B(off)} NMR spectra for compound **2** (bottom) and for its protonated derivative **4** (top).

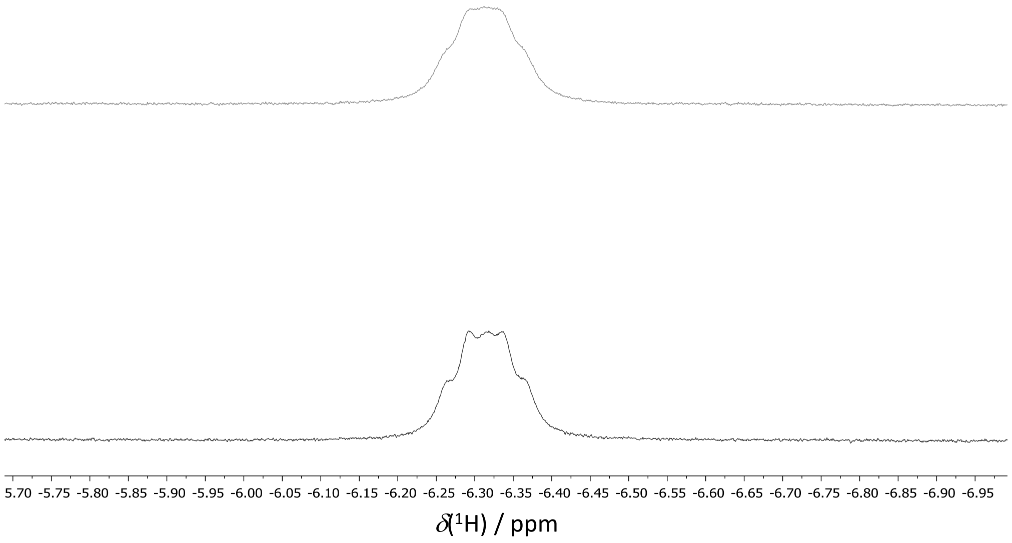

Figure S4 Room temperature ^{1}H–{^{11}B(BB)} (bottom) ^{1}H–{^{11}B(off)} (top) NMR spectra for protonated derivative **4**.

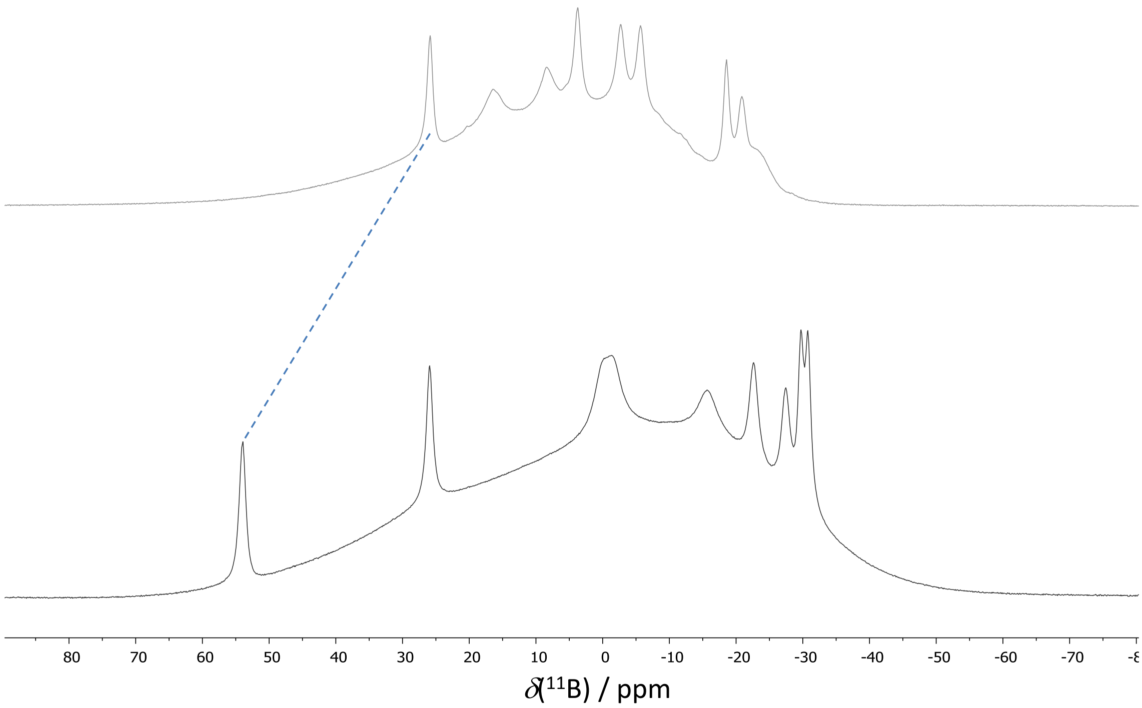

Figure S5 ^{11}B–{^{1}H} NMR spectra for compound **3** (bottom) and for its protonated derivative **5** (top).

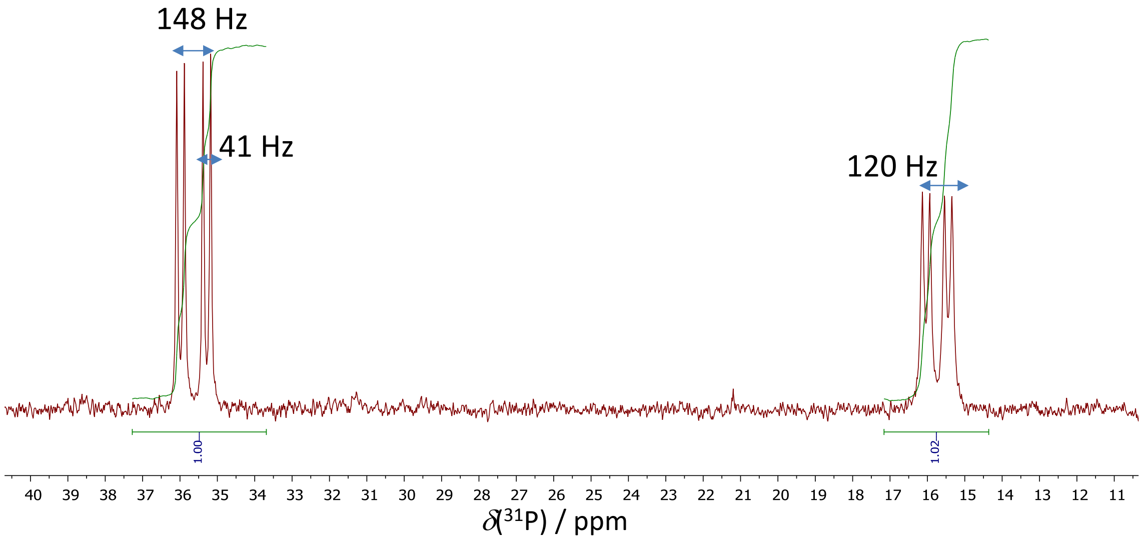

Figure S6 ^{31}P–{^{1}H} NMR spectra for compound **5**.

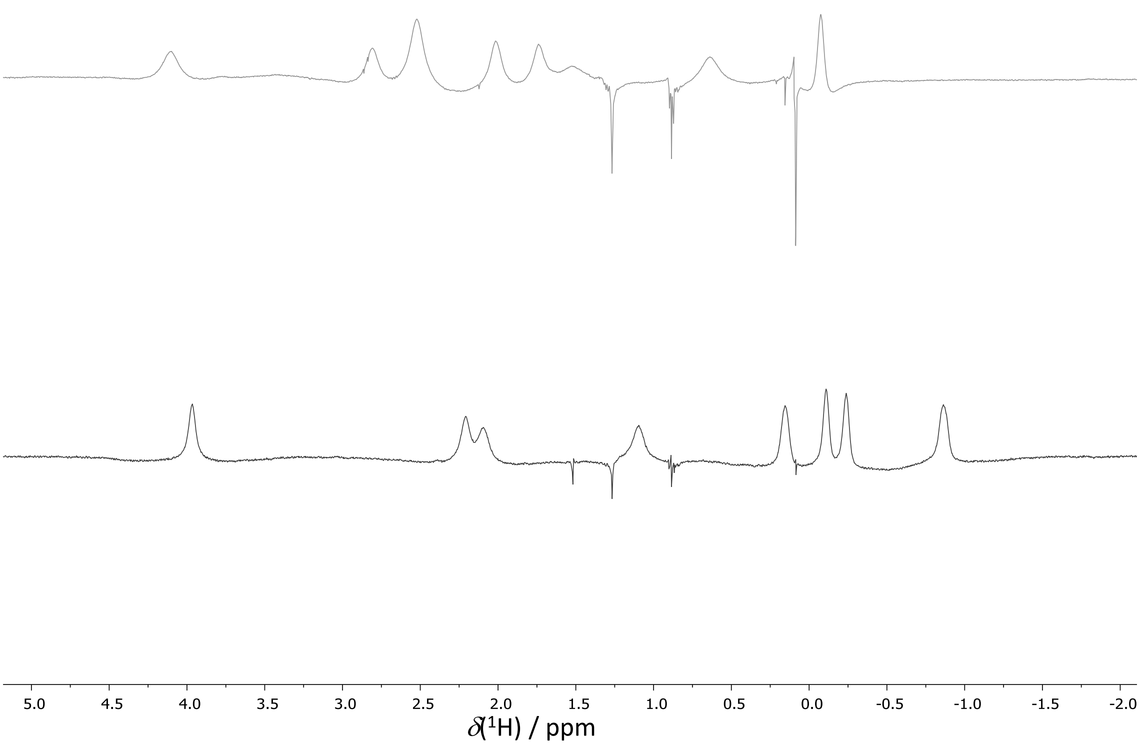

Figure S7 Room temperature $^{1}H–\{^{11}B(BB)\}$ – $^{1}H–\{^{11}B(off)\}$ NMR spectra for compound **3** (bottom) and for its protonated derivative **5** (top).

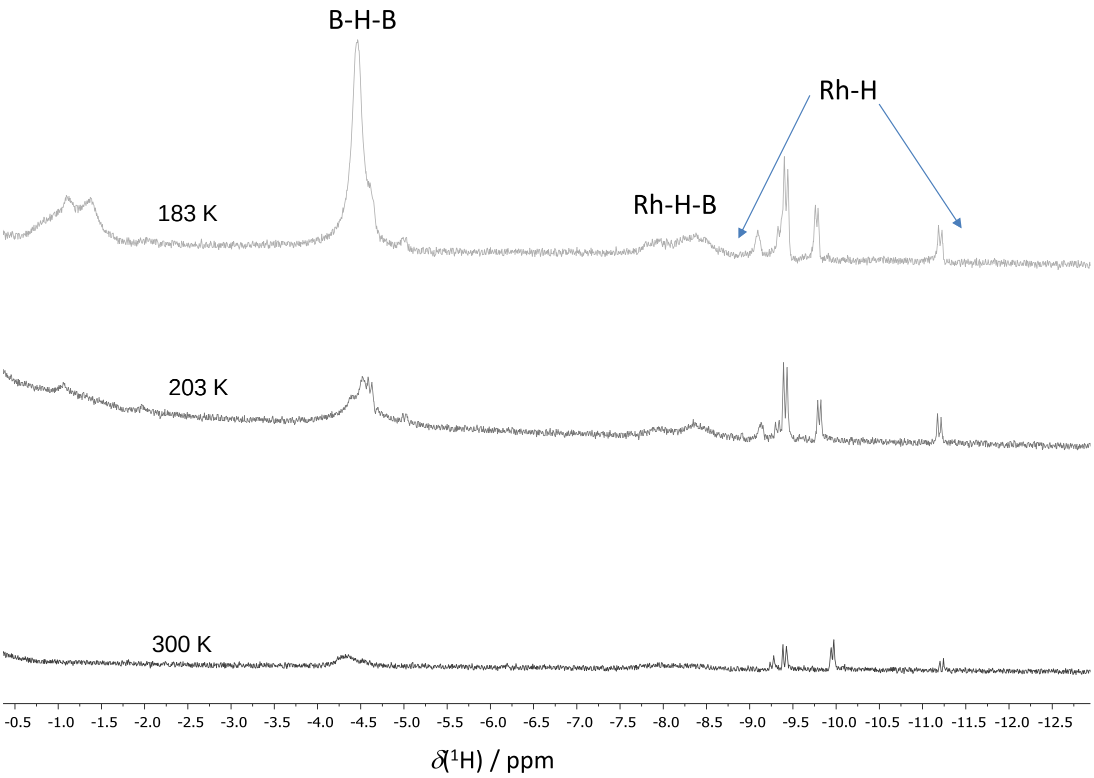

Figure S8 Room temperature $^{1}H–\{^{11}B\}$ NMR spectra for the triflic acid reaction system **5** at different temperatures in CD_2Cl_2.

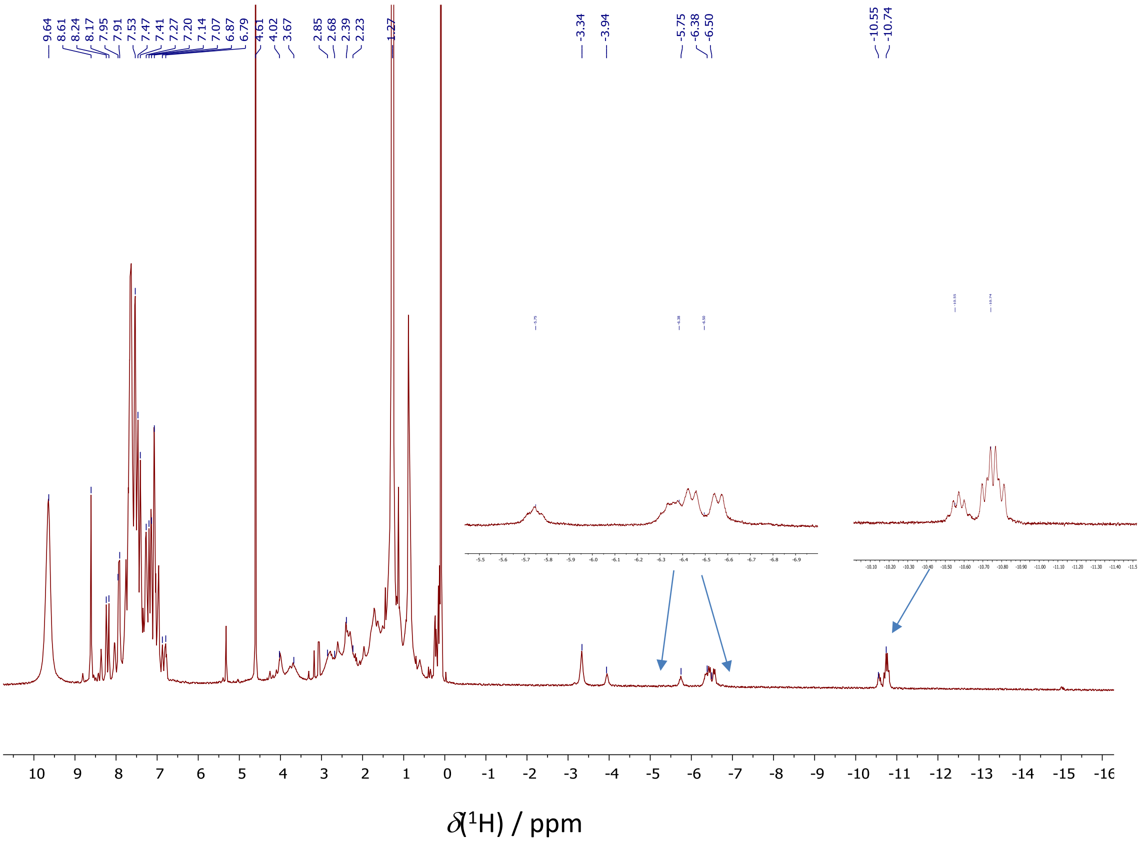

Figure S9 Room temperature $^{1}H–\{^{11}B\}$ NMR spectrum for the triflic acid reaction system **4** under an atmosphere of H_2, in CD_2Cl_2.

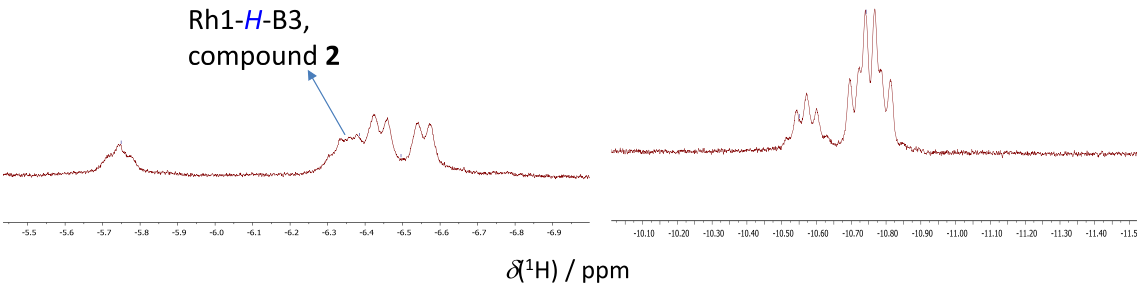

Figure S10 $^{1}H–\{^{11}B\}$ NMR spectrum, in the negative region, for the reaction mixture of **4** with H_2.

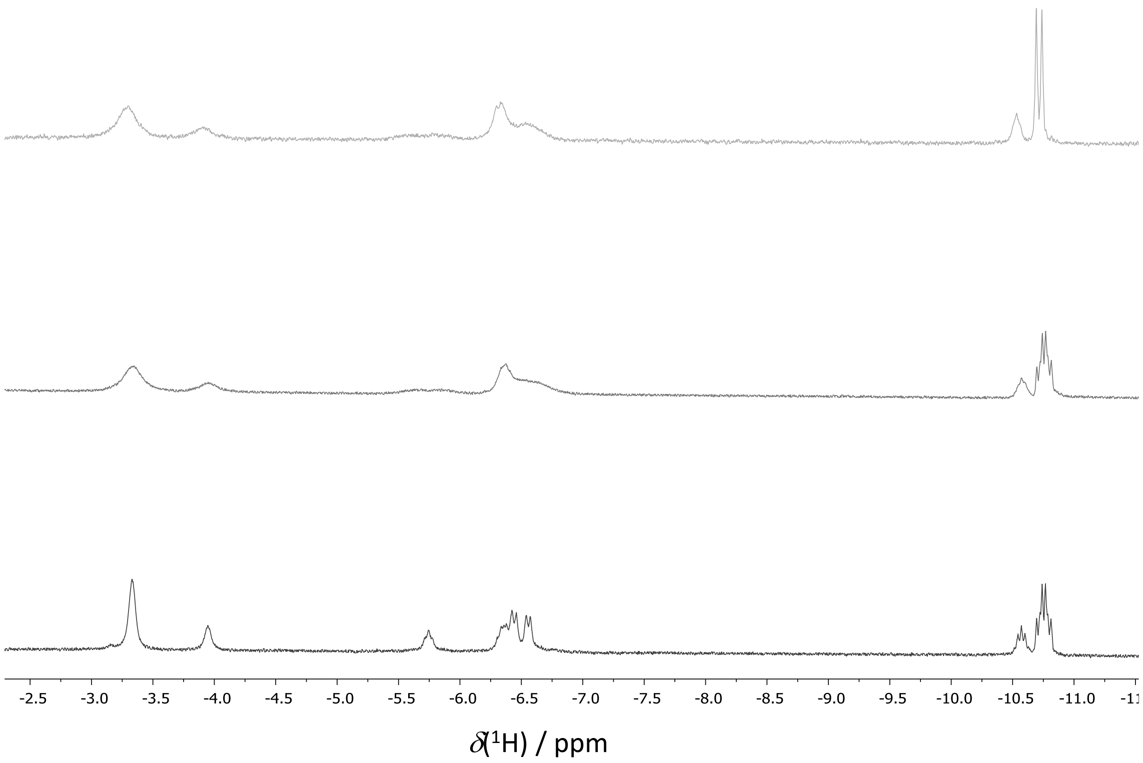

Figure S11 Negative region of the room temperature $^{1}H–\{^{11}B\}$ (bottom), $^{1}H–\{^{11}B(off)\}$ (middle) and $^{31}P–\{^{1}H\}$ (bottom) NMR spectra for the triflic acid reaction system **4** under an atmosphere of H_2, in CD_2Cl_2.

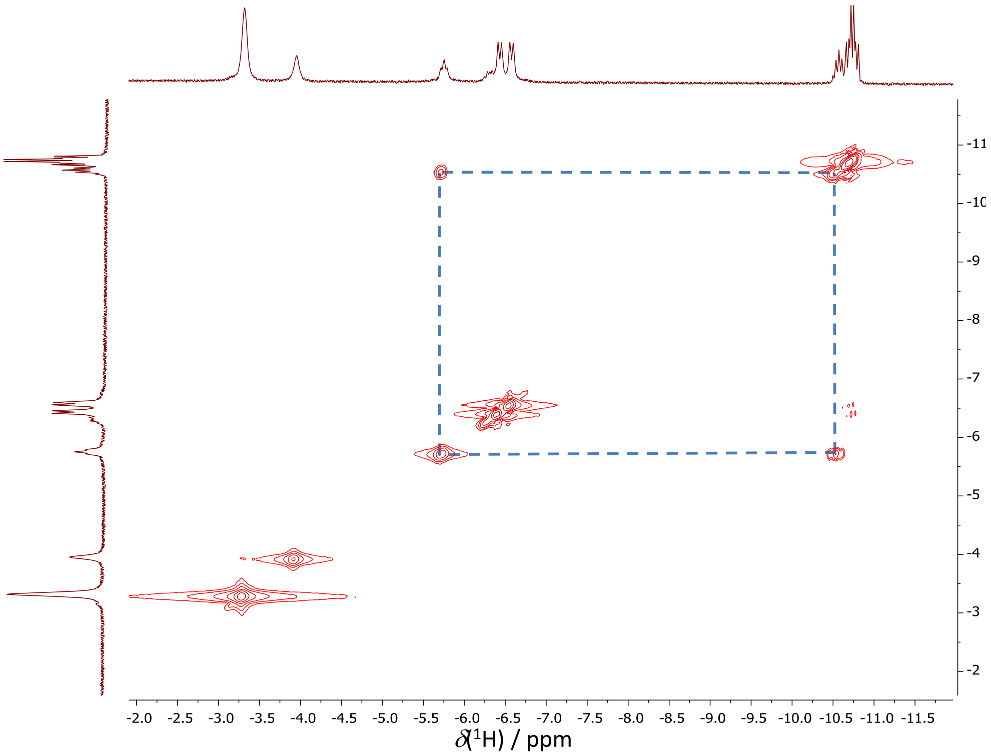

Figure S12 [^{1}H–^{1}H–{^{11}B}]–COSY spectrum of **4** under an atmosphere of H_2, in the negative region at room temperature in CD_2Cl_2.

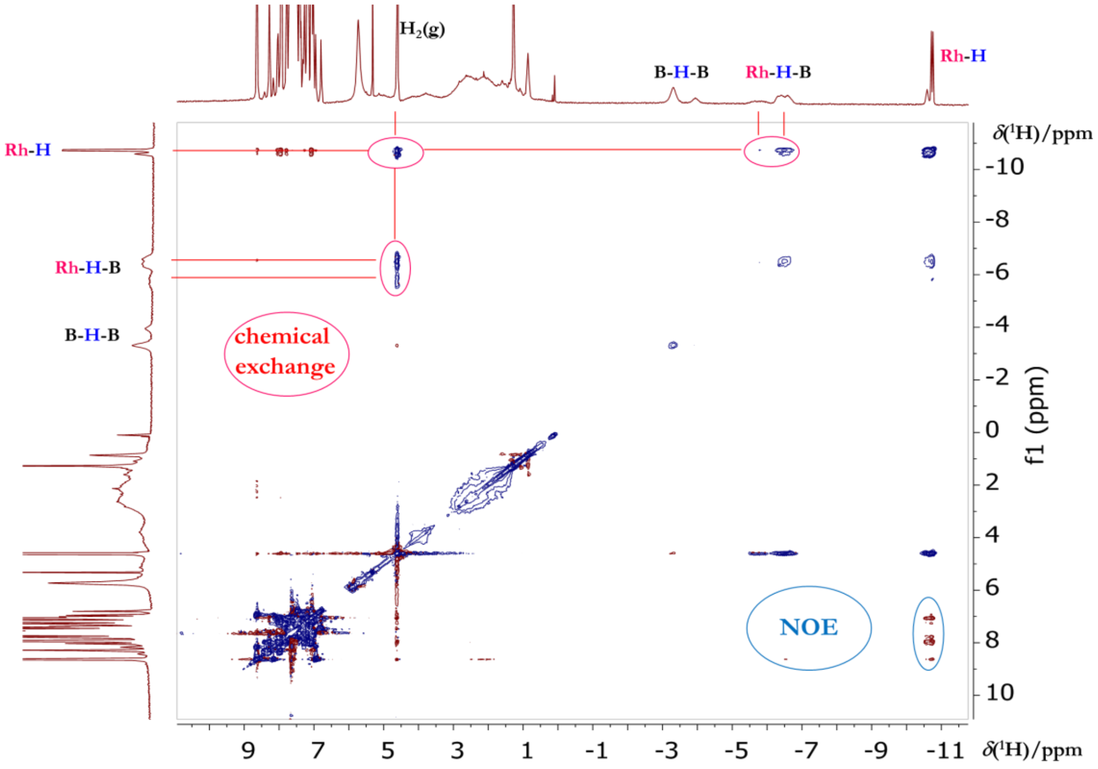

Figure S13 [^{1}H–^{1}H]–NOESY spectrum of **4** under an atmosphere of H_2 at room temperature in CD_2Cl_2.

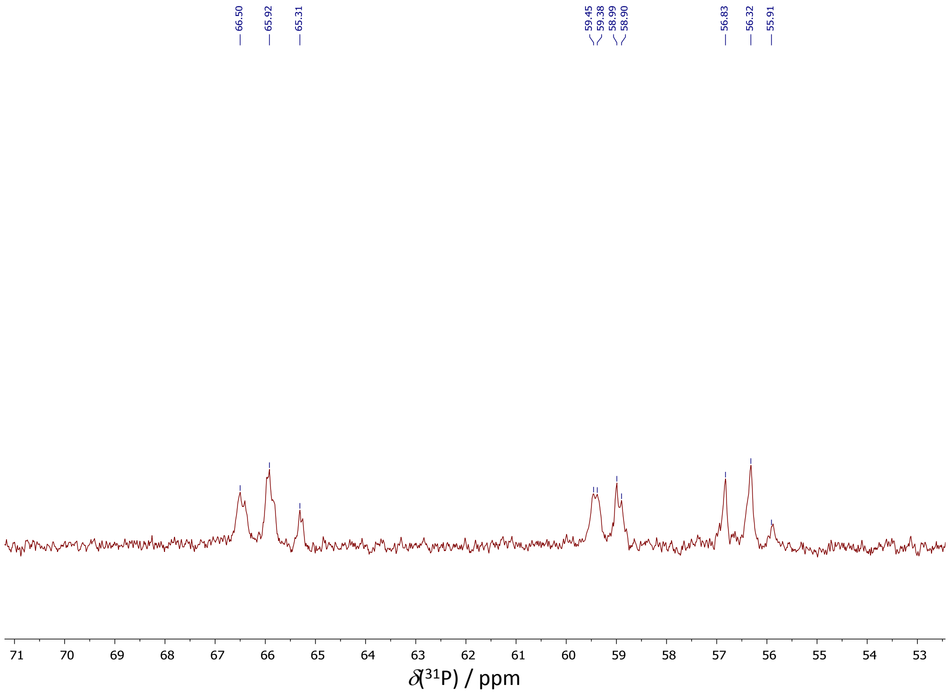

Figure S14 ^{31}P–{^{1}H} NMR spectra for compound **4** under an atmosphere of H_2 at room temperature in CD_2Cl_2.

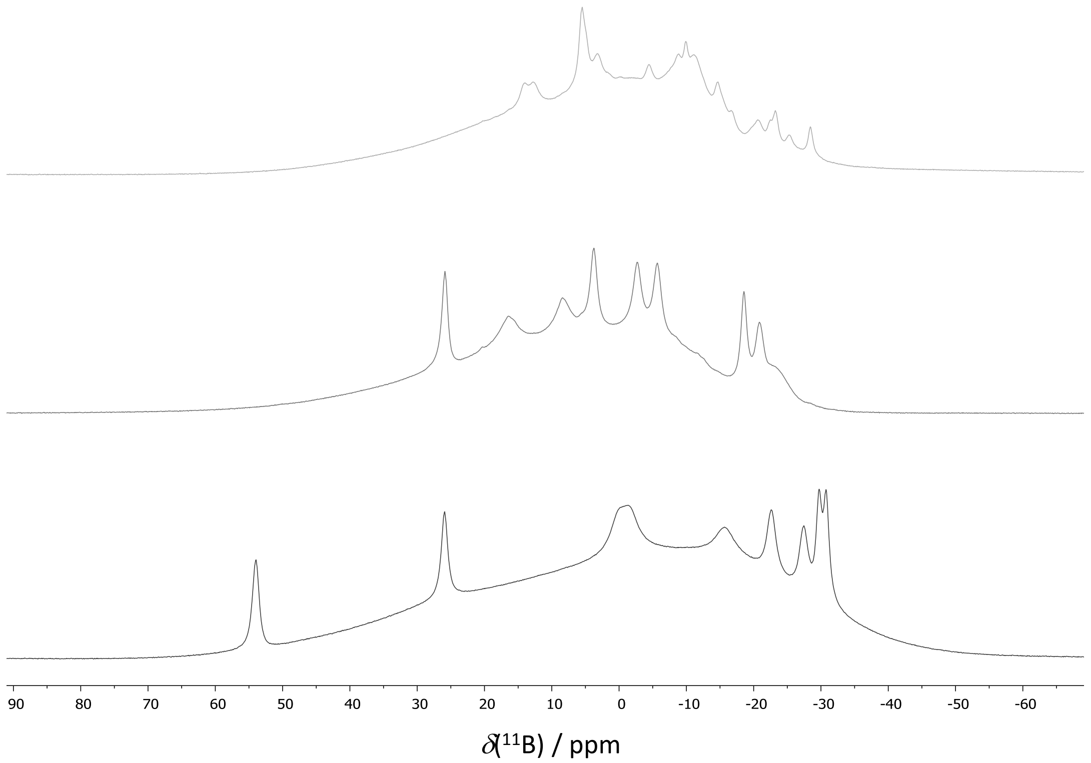

Figure S15 ^{11}B–{^{1}H} NMR spectra of neutral **3** (bottom), cationic system **5** (middle), and **5** under an atmosphere of H_2 (top) at room temperature in CD_2Cl_2.

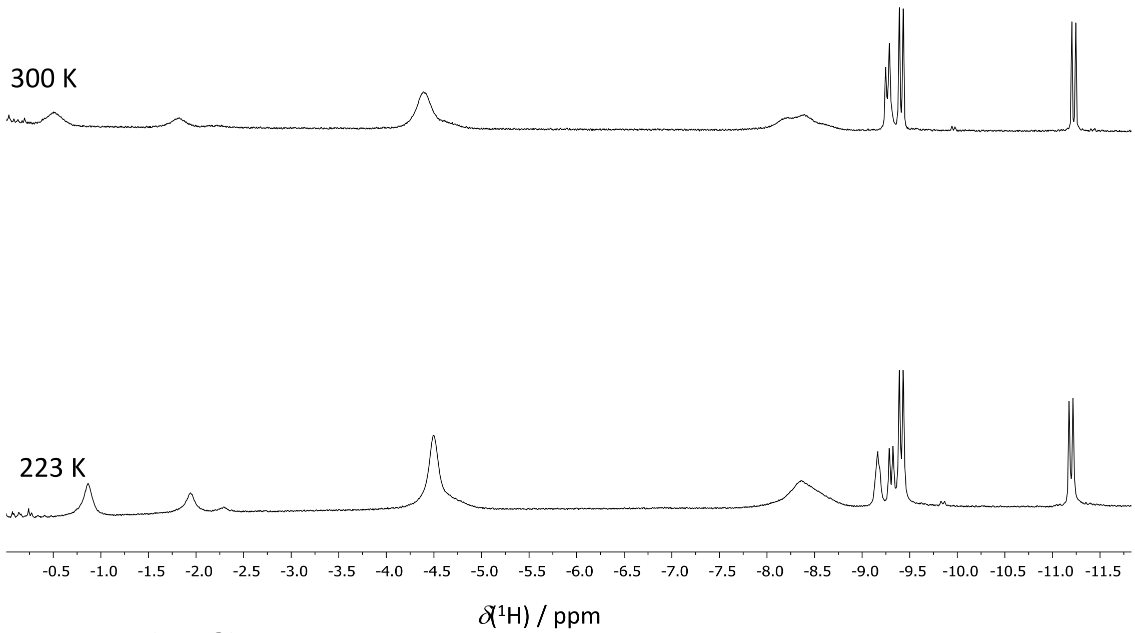

Figure S16 ^{1}H–{^{31}P} spectra at different temperatures for a sample of **5** under an atmosphere of H_2 in CD_2Cl_2.

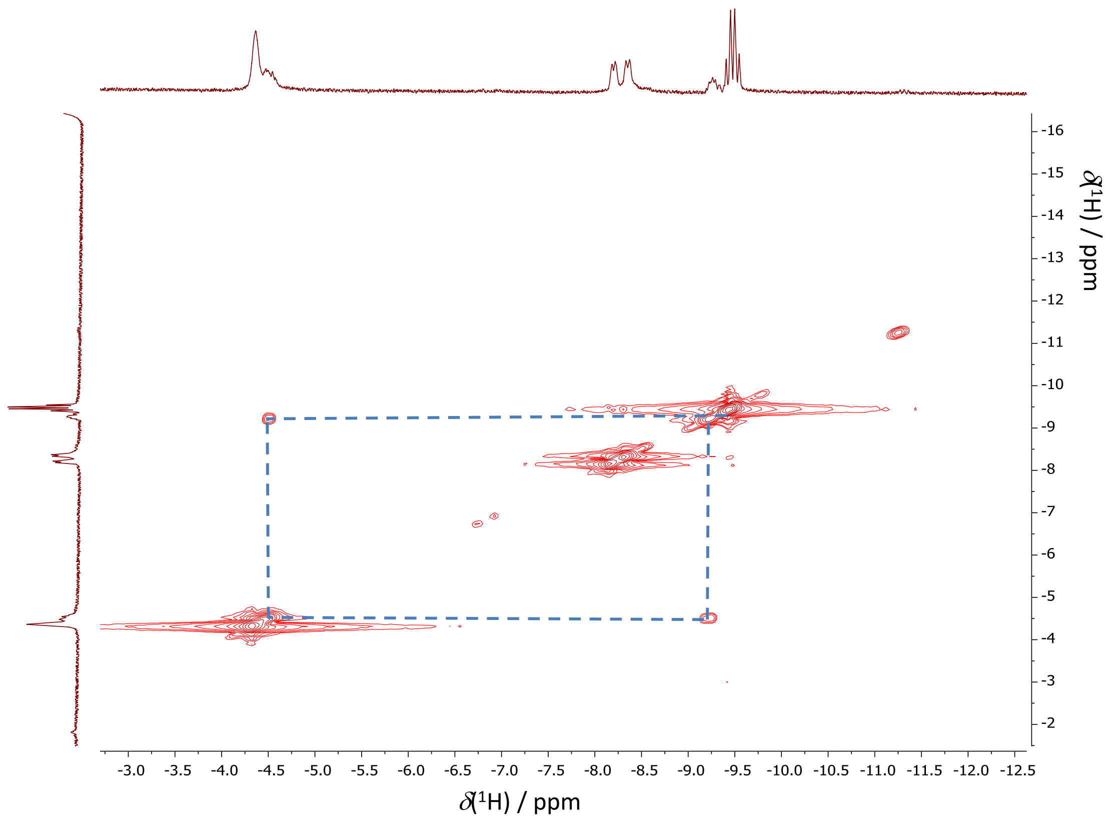

Figure S17 [^{1}H–^{1}H–{^{11}B}]–COSY spectrum for a sample of **5** under an atmosphere of H_2 in CD_2Cl_2.

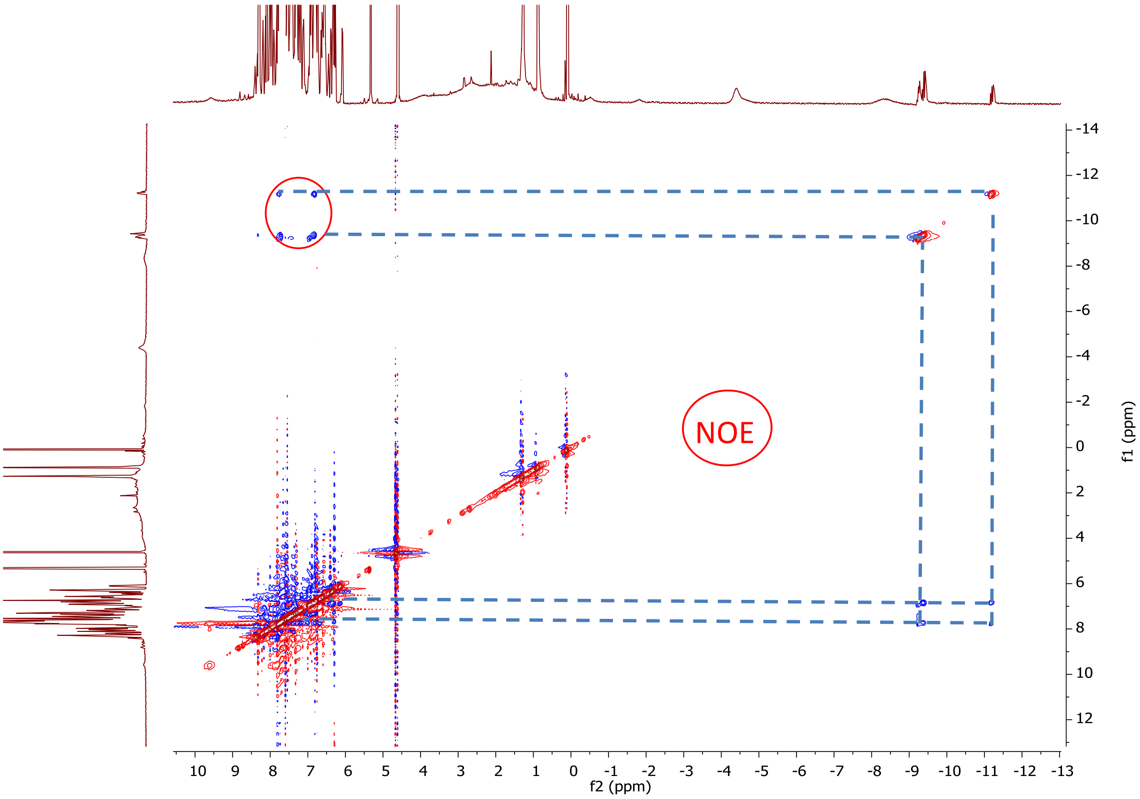

Figure S18 [^{1}H-^{1}H]-NOESY, 400 MHz, CD_2Cl_2, 300 K, for system **8**.

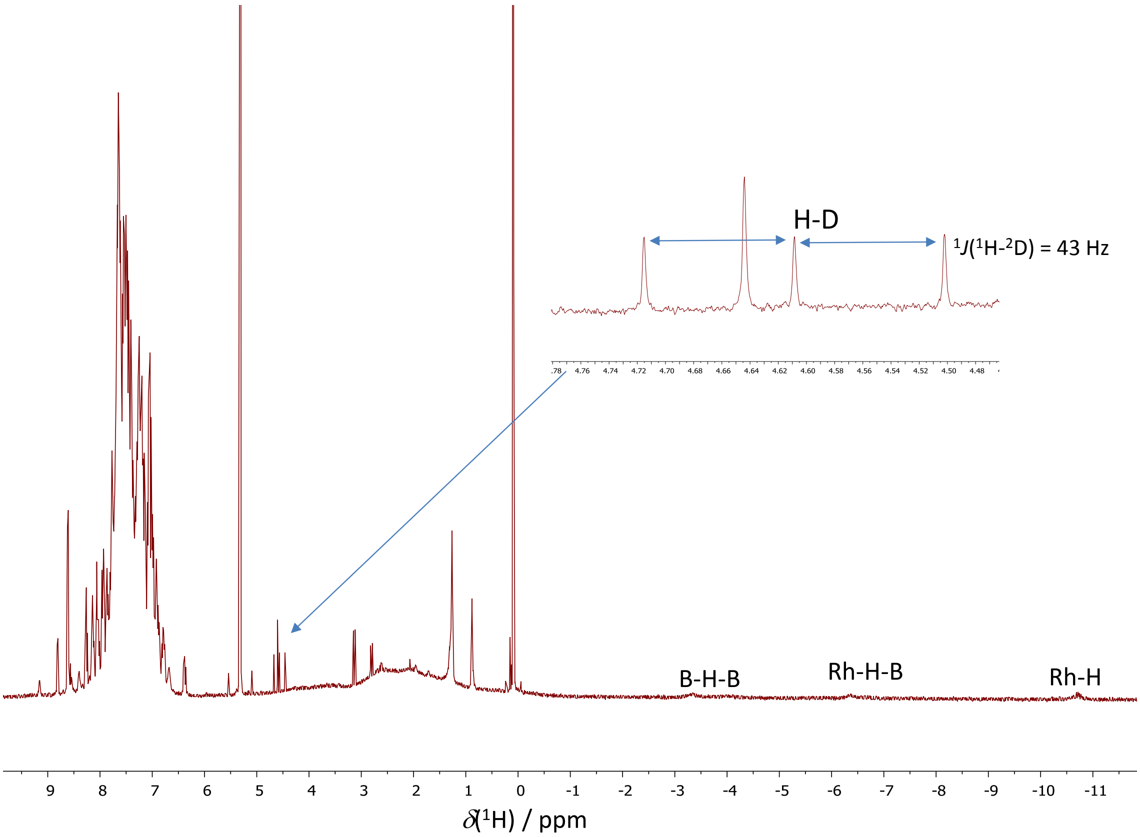

Figure S19^{1}H NMR spectra for compound **4** under an atmosphere of D_2 at room temperature in CD_2Cl_2.

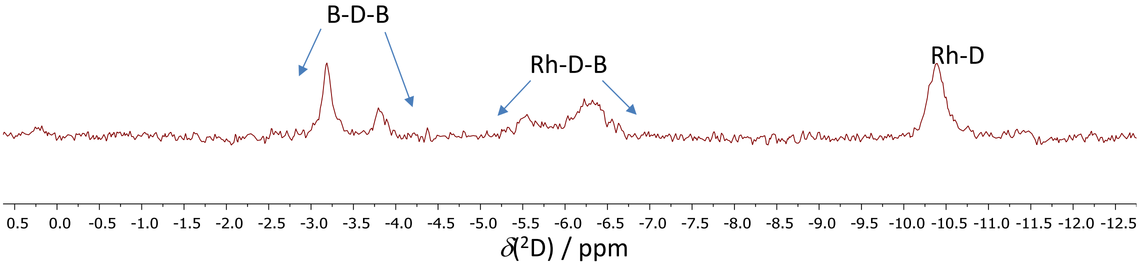

Figure S20 ^{2}D NMR spectra for compound **4** under an atmosphere of D_2 at room temperature in CH_2Cl_2.

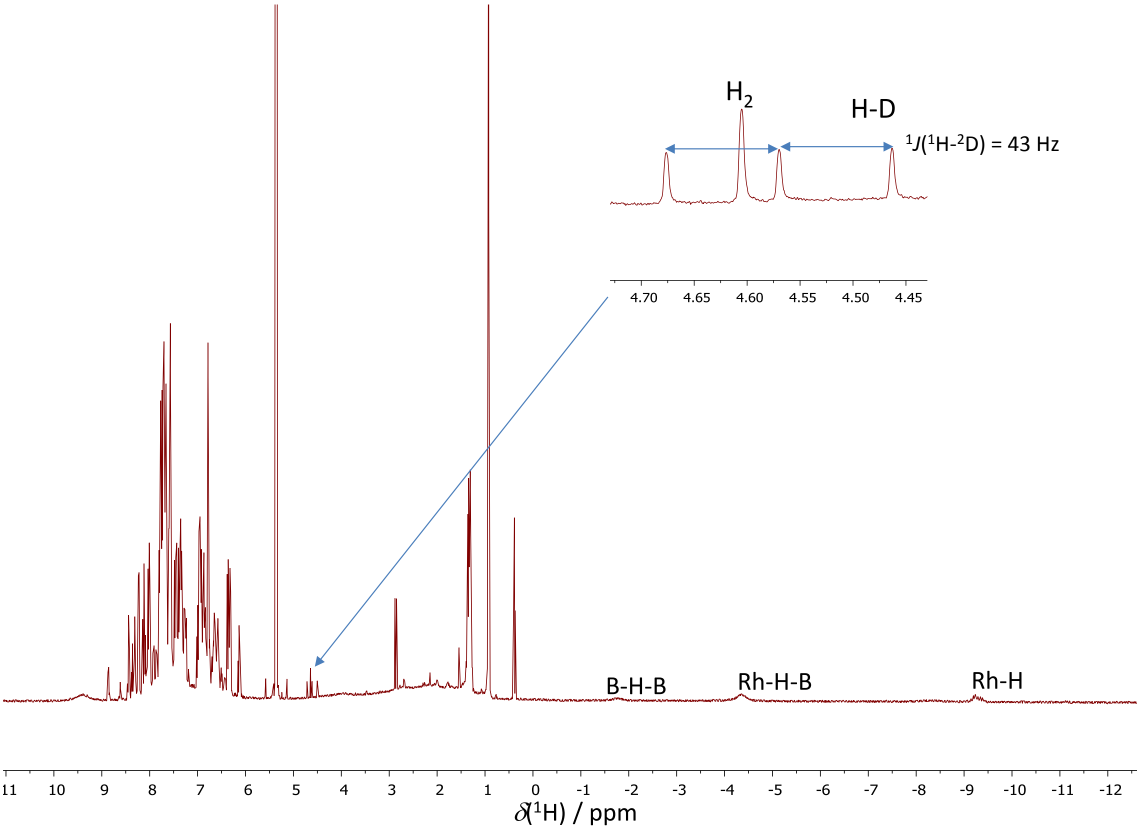

Figure S21 ^{1}H NMR spectra for compound **5** under an atmosphere of D_2 at room temperature in CD_2Cl_2.

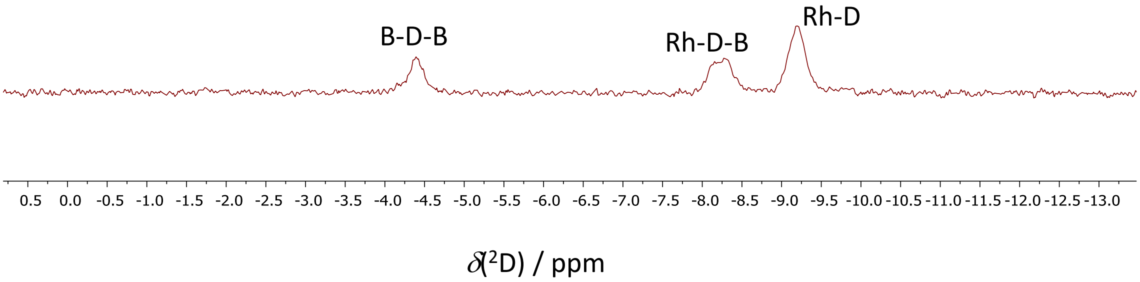

Figure S22 ^{2}D NMR spectra for compound **5** under an atmosphere of D_2 at room temperature in CH_2Cl_2.

.

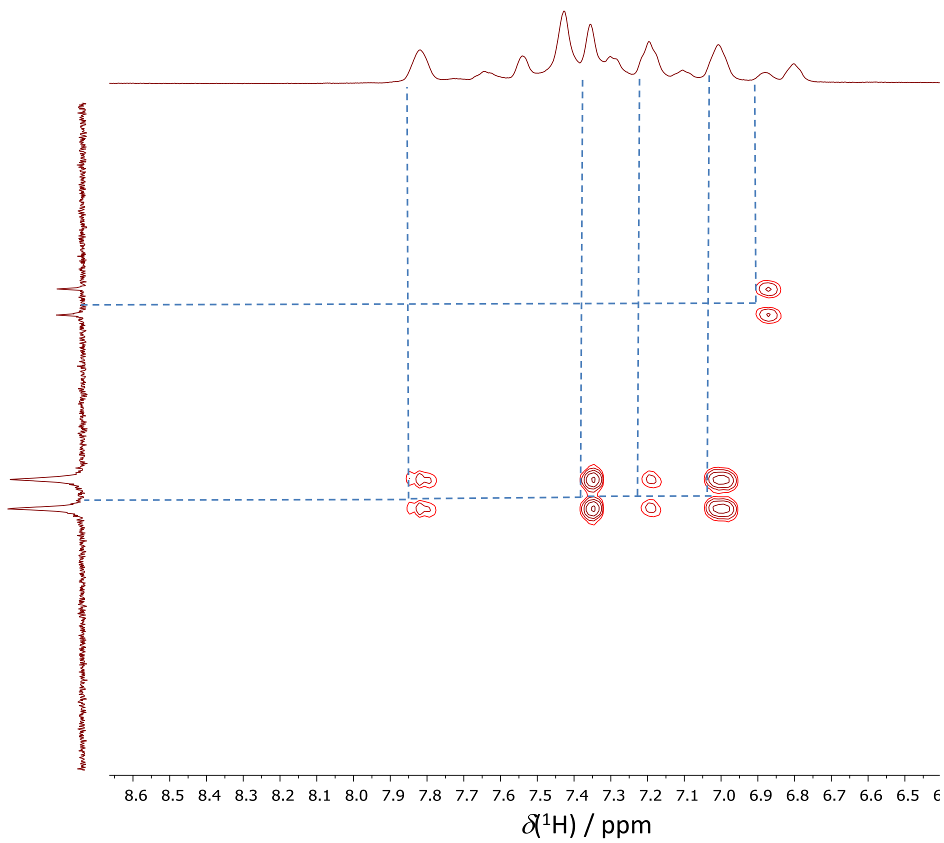

Figure S23 [^{1}H-^{31}P]-HMBC, 202 MHz, CD_2Cl_2, 300 K for compound **2**.

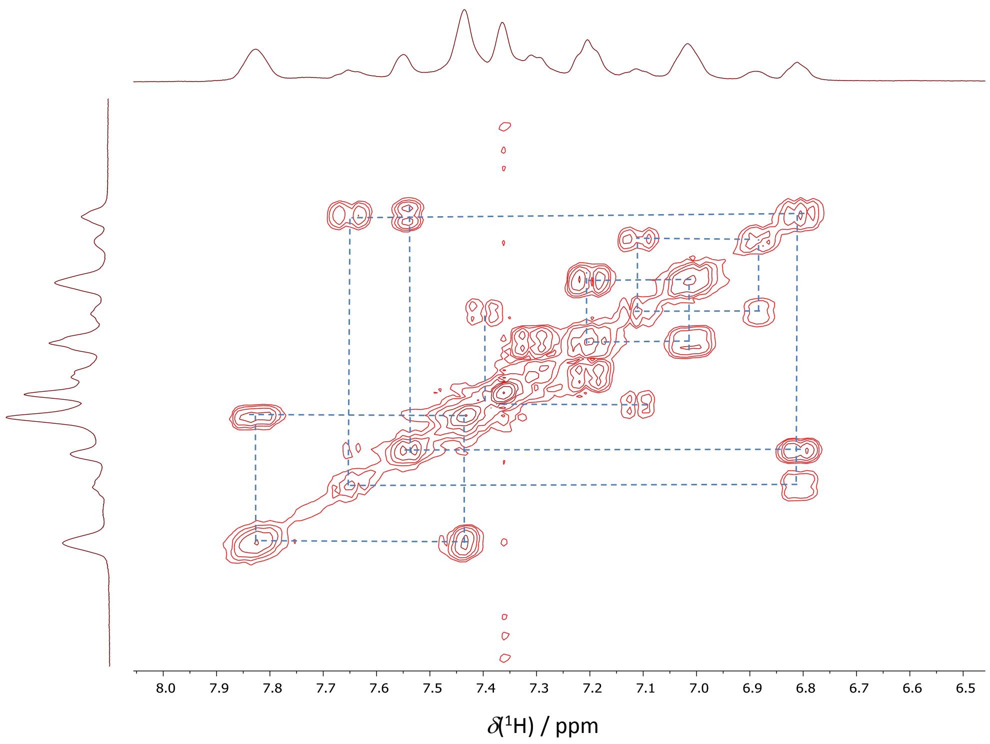

Figure S24 [1H-1H]-COSY, 400 MHz, CD_2Cl_2, 300 K, for **2**.

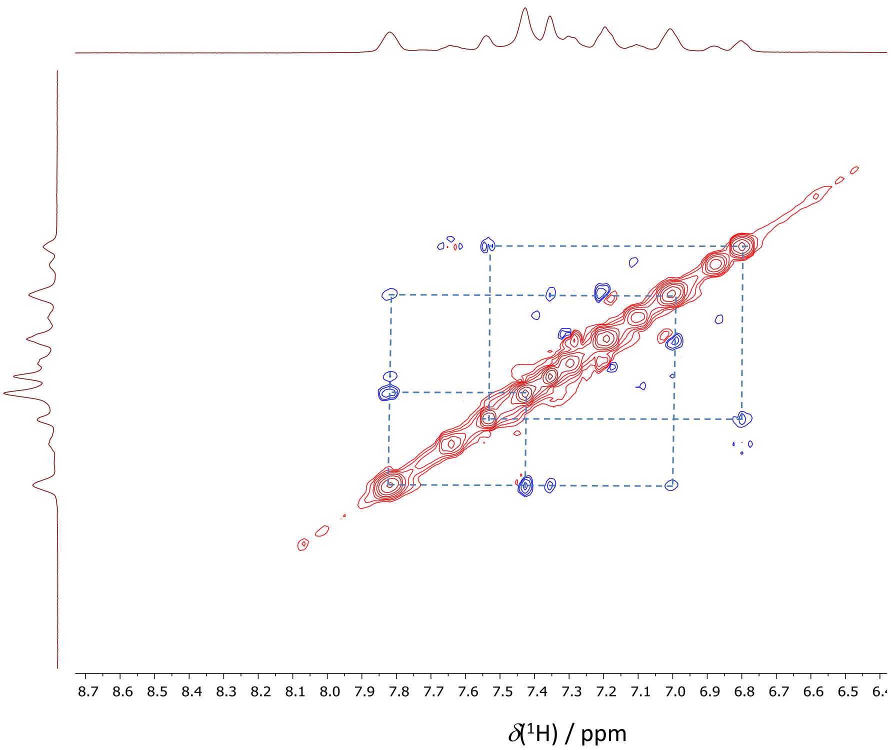

Figure S25 [1H-1H]-NOESY, 400 MHz, CD_2Cl_2, 300 K, for **2**.

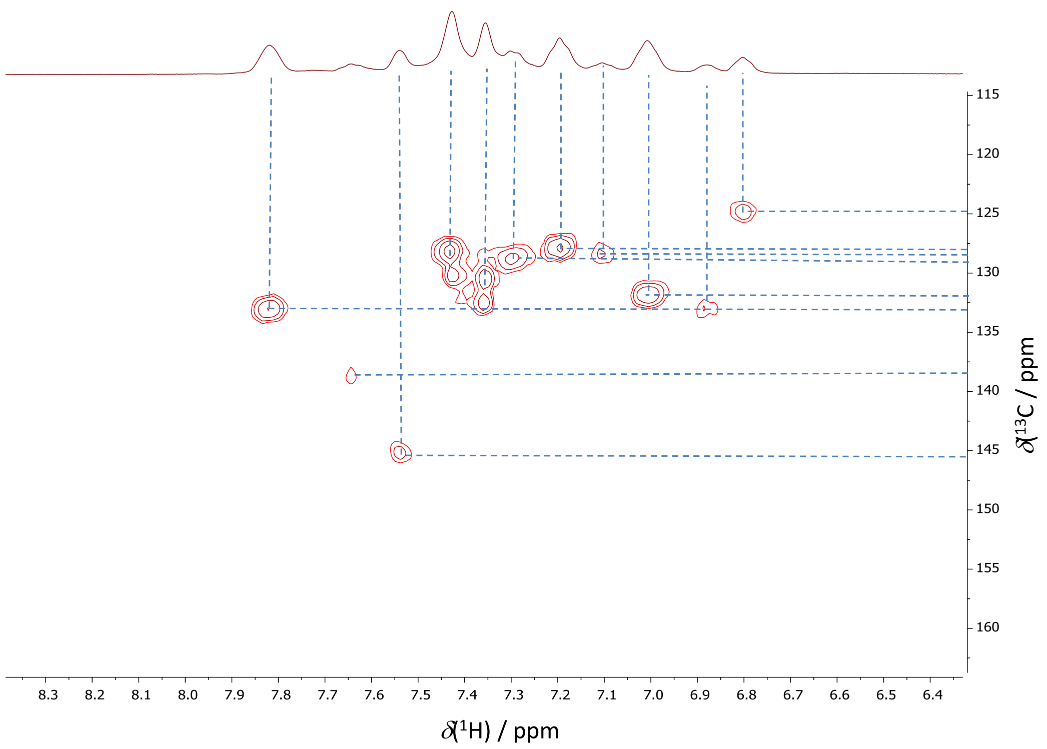

Figure S26 [^{1}H-^{13}C]-HSQCH, 100 MHz, CD_2Cl_2, 300 K, for **2**.

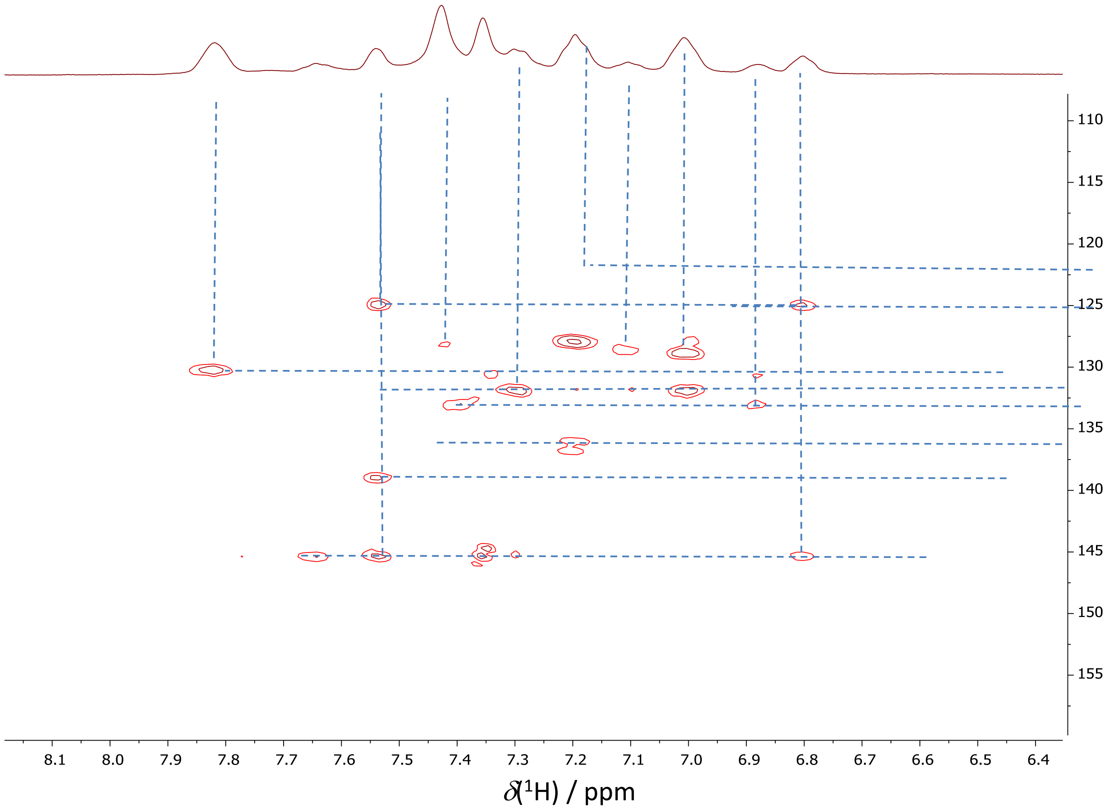

Figure S27 [^{1}H-^{13}C]-HMBC, 100 MHz, CD_2Cl_2, 300 K, for **2**.

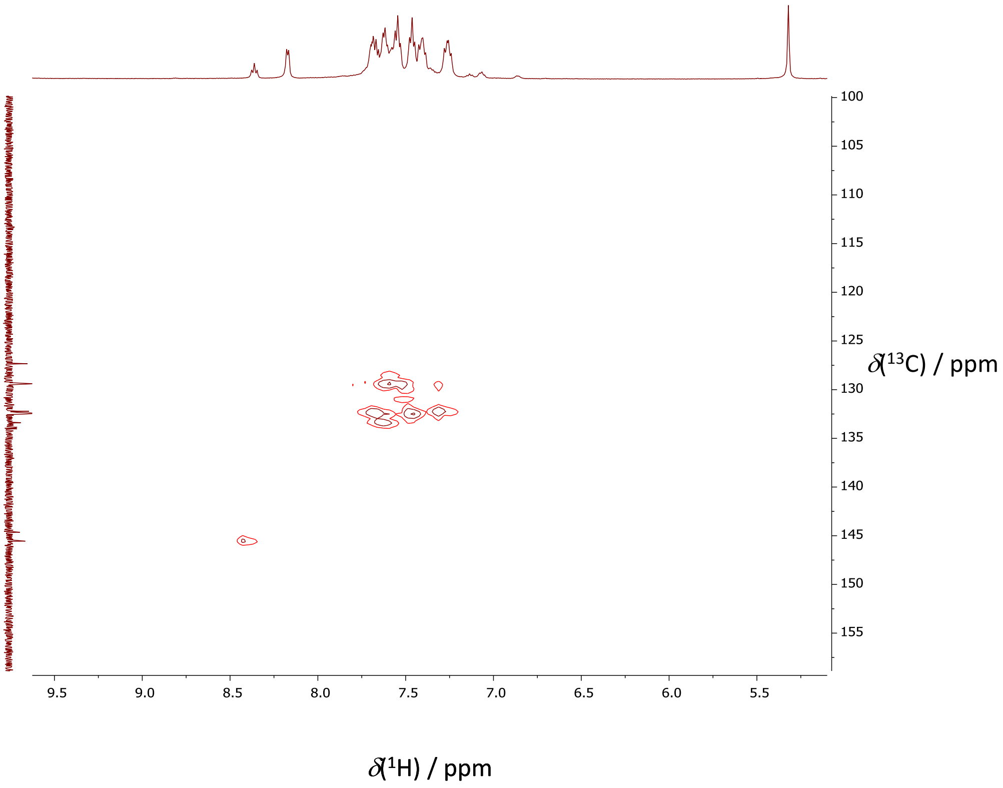

Figure S28 [^{1}H-^{13}C]-HMBC, 100 MHz, CD_2Cl_2, 300 K, for **4**.

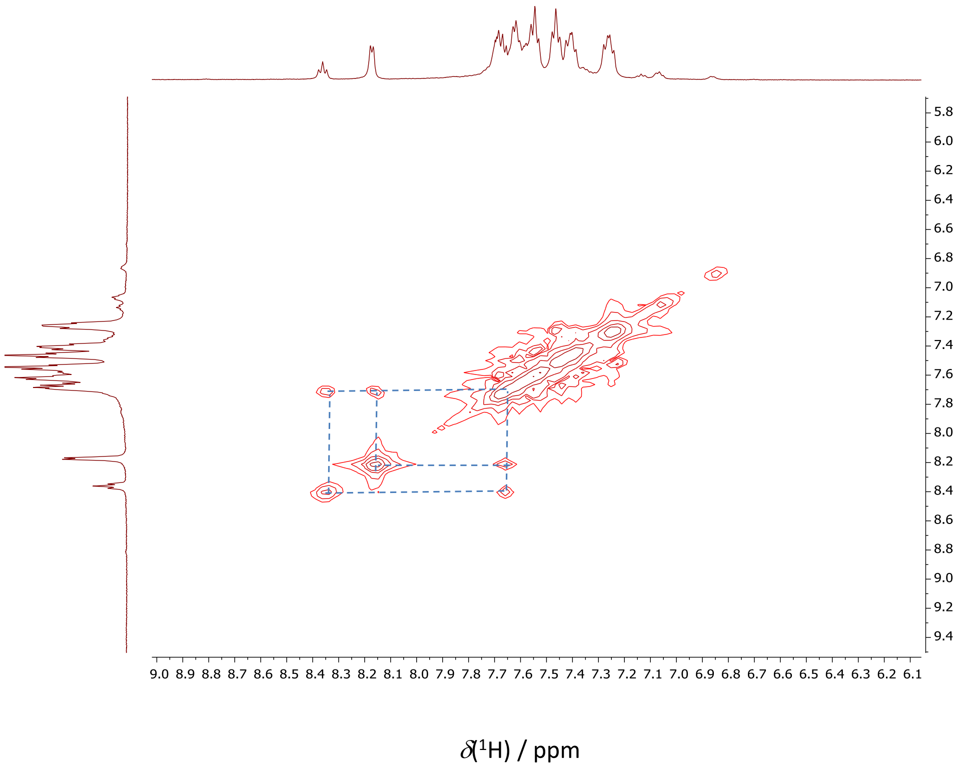

Figure S29 [^{1}H-^{1}H]-COSY, 400 MHz, CD_2Cl_2, 300 K, for **4**.

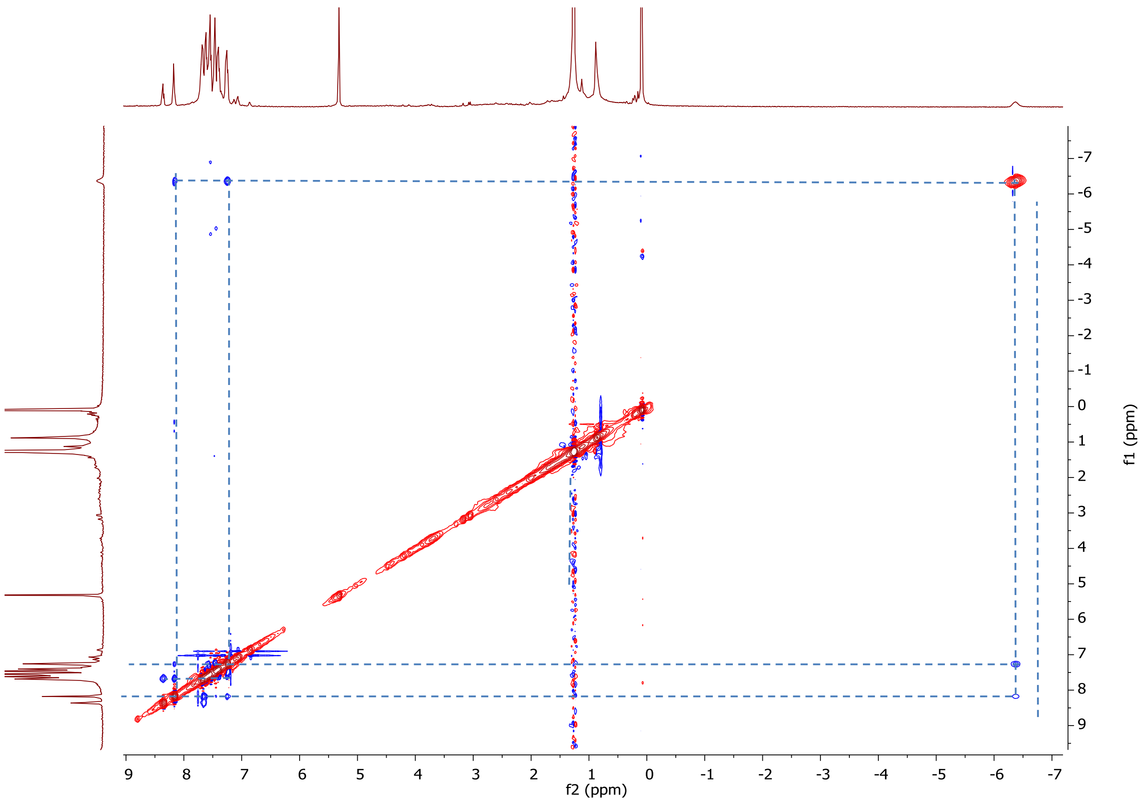

Figure S30 [^{1}H-^{1}H]-NOESY, 400 MHz, CD_2Cl_2, 300 K, for **4**.

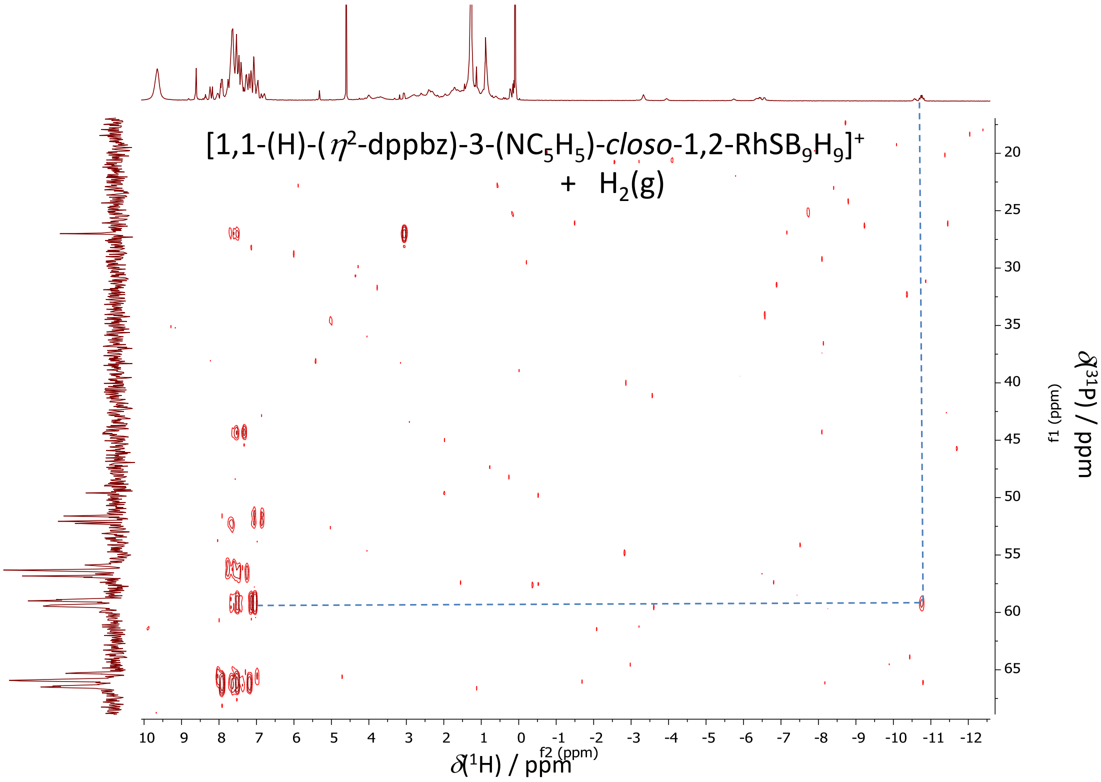

Figure S31 [^{1}H-^{31}P]-HMBC, 400 MHz, CD_2Cl_2, 300 K, for **6**.

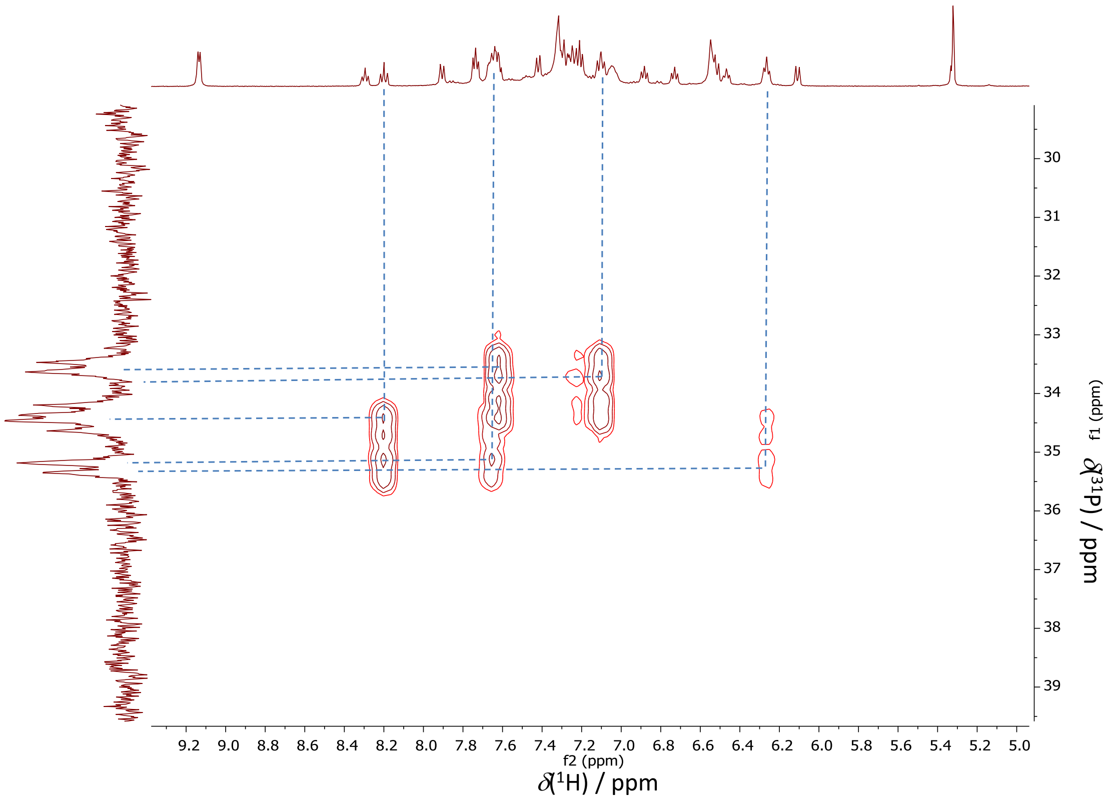

Figure S32 [^{1}H-^{31}P]-HMBC, 400 MHz, CD_2Cl_2, 300 K, for **3**.

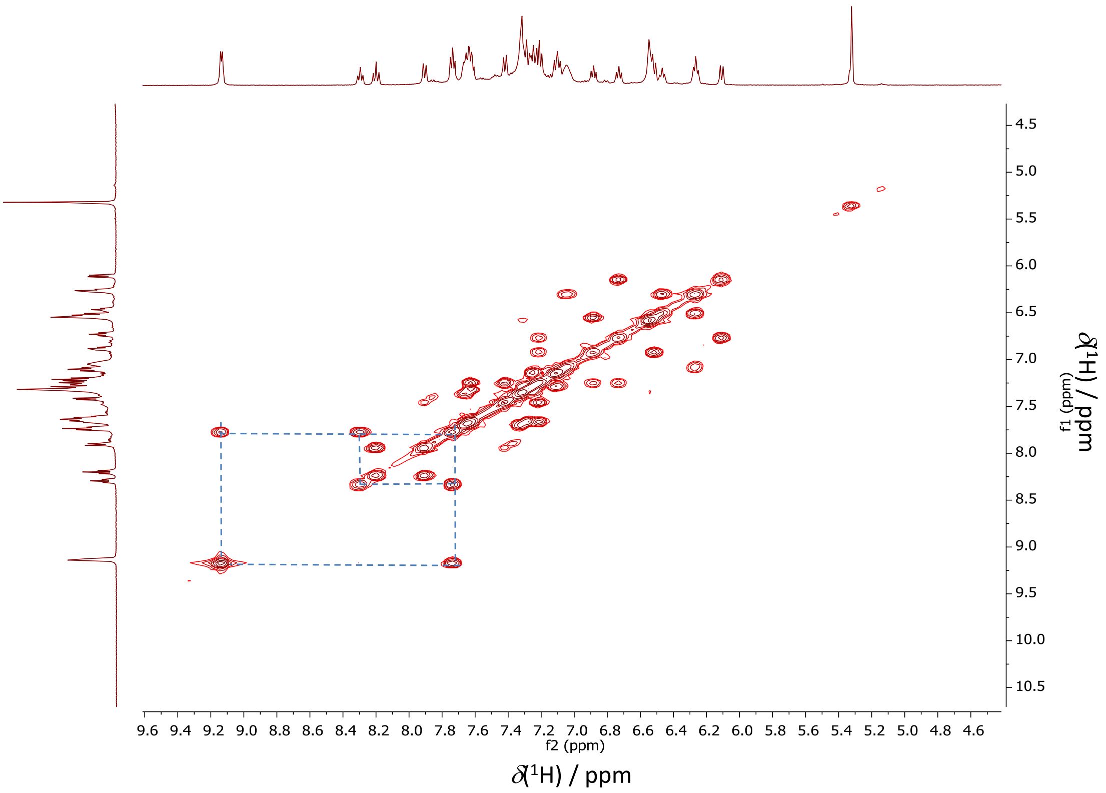

Figure S33 [^{1}H-^{1}H]-COSY, 400 MHz, CD_2Cl_2, 300 K, for **3**.

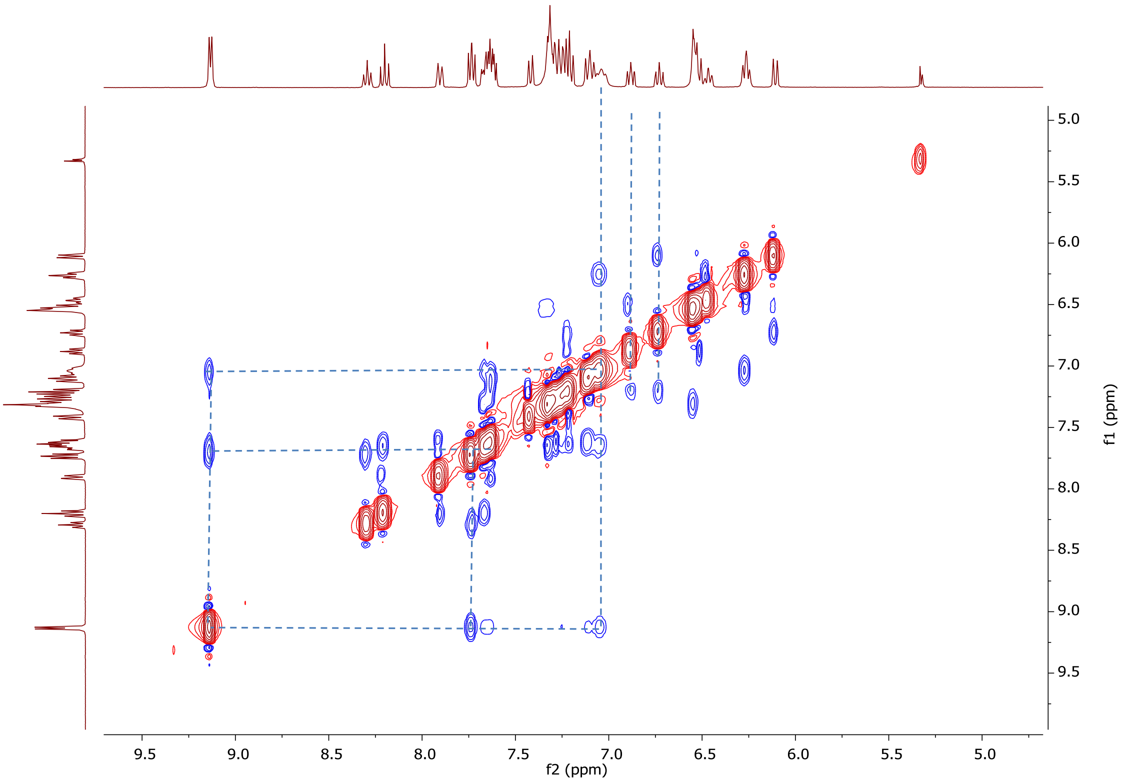

Figure S34 [^{1}H-^{1}H]-NOESY, 400 MHz, CD_2Cl_2, 300 K, for **3**.

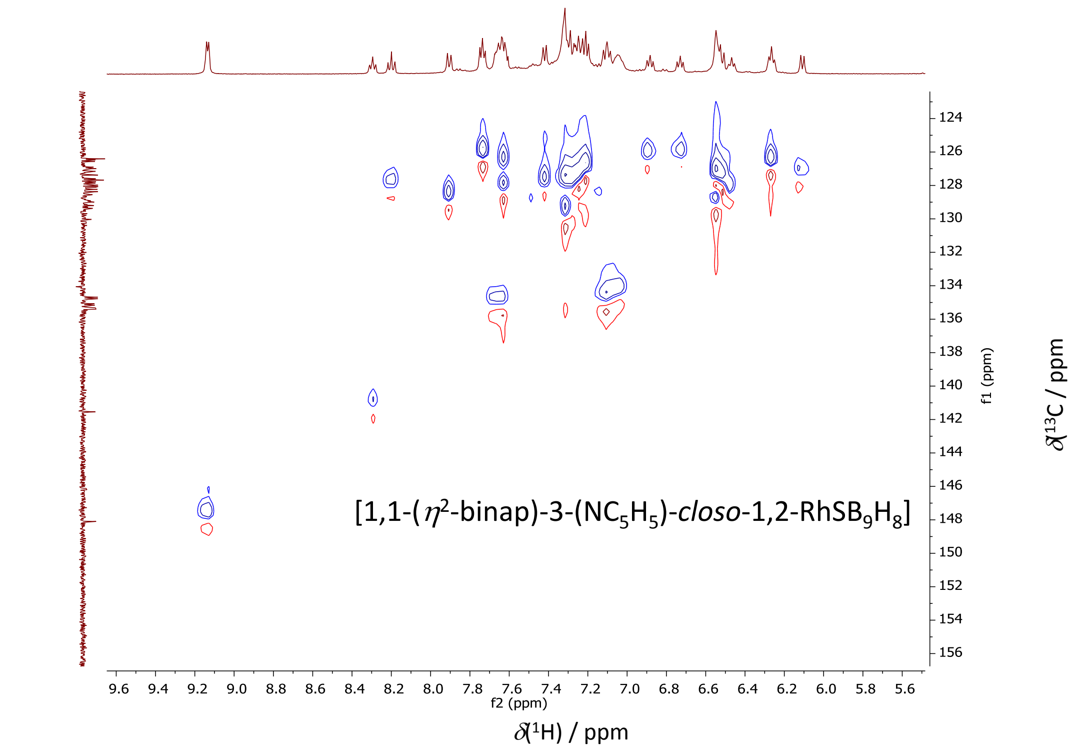

Figure S35 [^{1}H-^{13}C]-HSQC, 400 MHz-100 MHz, CD_2Cl_2, 300 K, for **3**.

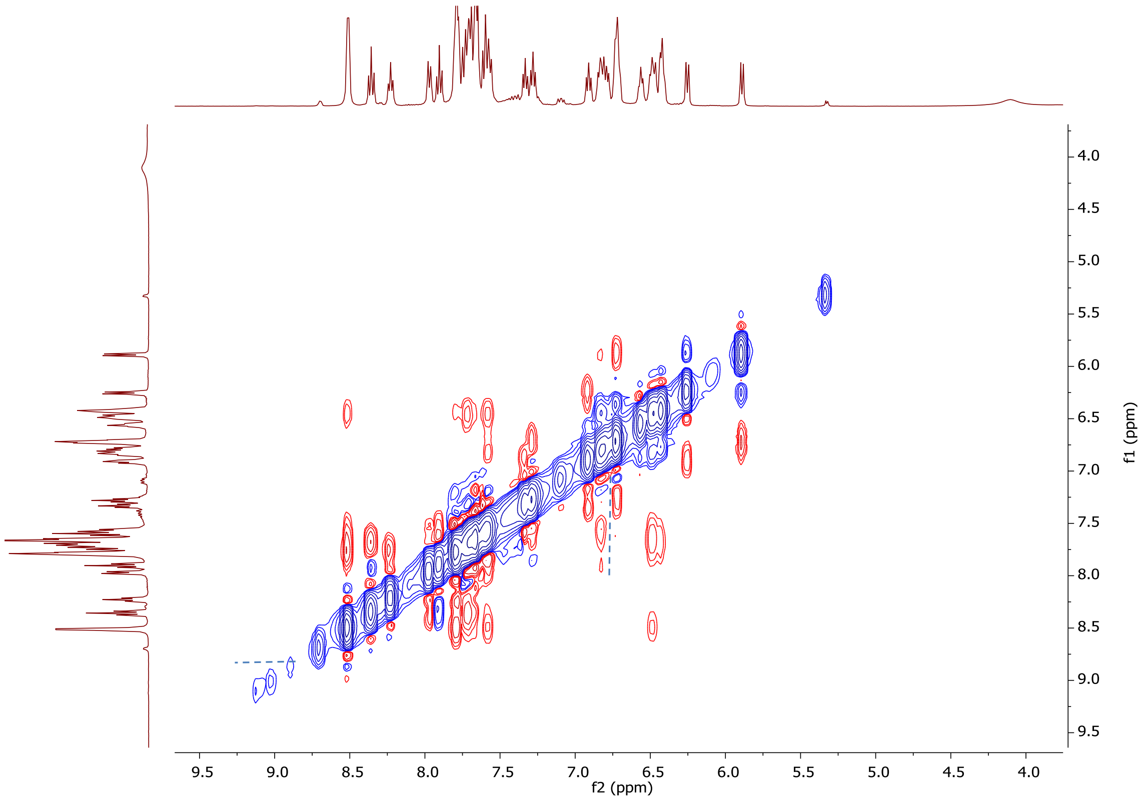

Figure S36 [^{1}H-^{1}H]-NOESY, 400 MHz, CD_2Cl_2, 300 K, for **5**.

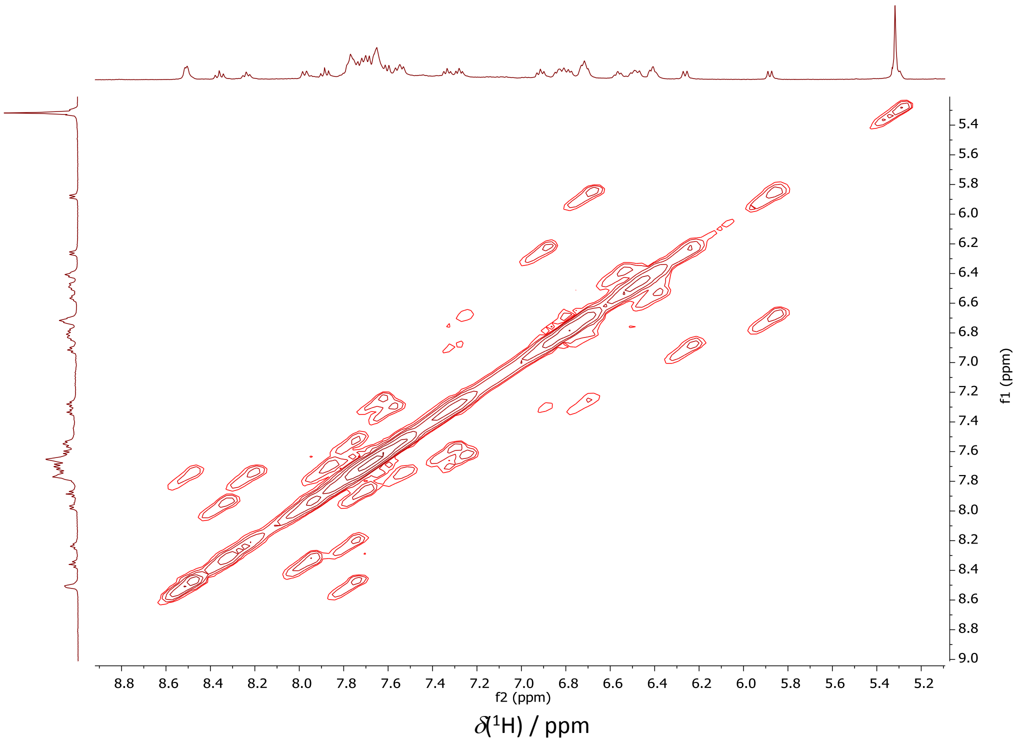

Figure S37 [^{1}H-^{1}H]-COSY, 400 MHz, CD_2Cl_2, 300 K, for **5**.

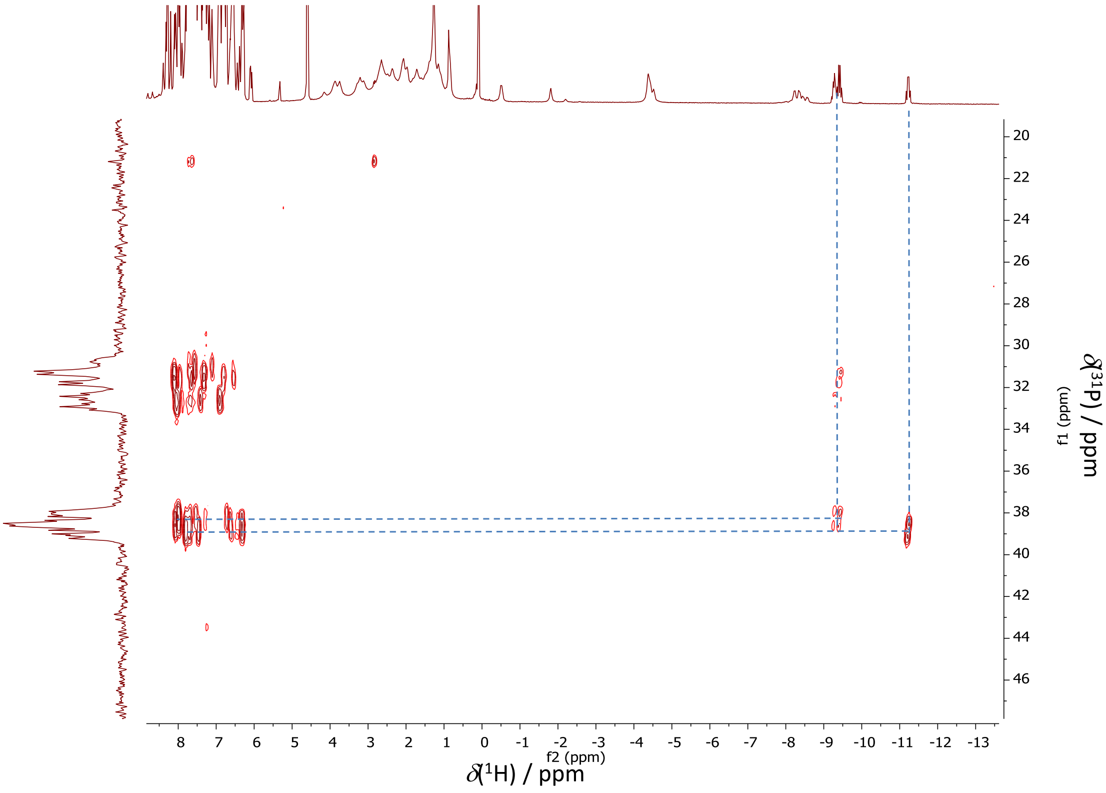

Figure S38 [^{1}H-^{31}P]-HMBC, 400 MHz, CD_2Cl_2, 300 K, for system **8**.

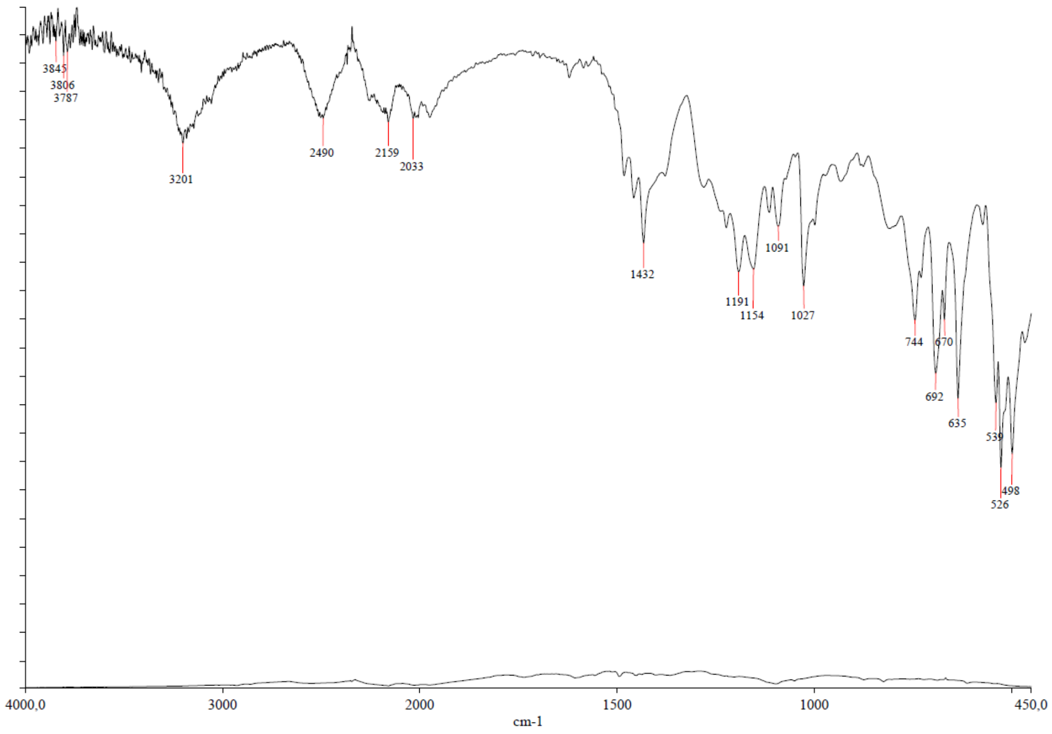

Figure S39 IR spectrum of compound **4**.

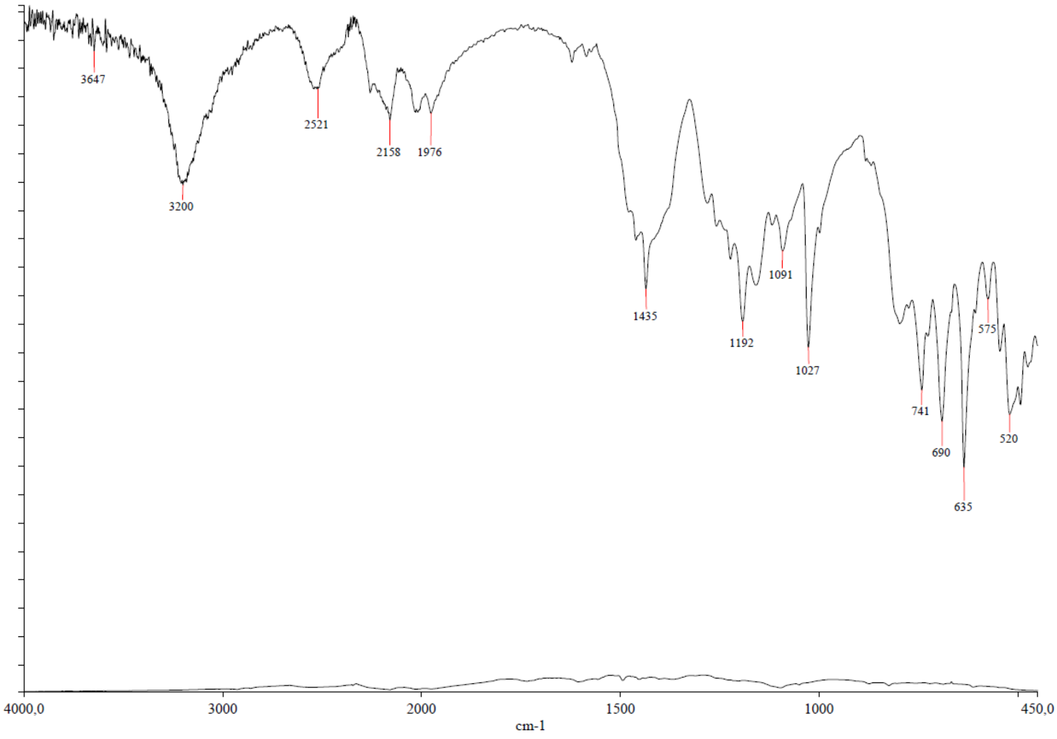

Figure S40 IR spectrum of compound **5**.
